# An interpretable multi‐task whole‐slide histopathology AI model for non‐small cell lung cancer: Cross‐cohort generalisation, spatial attention–transcriptomic integration, and molecular–immune profiling

**DOI:** 10.1002/ctm2.70744

**Published:** 2026-07-23

**Authors:** Renyi Lu, Anqi Lin, Aimin Jiang, Yuying Feng, Xiuhui Fang, Junyi Shen, Yifeng Bai, Shengkun Peng, Jian Zhang, Quan Cheng, Suyin Feng, Qinglin Li, Peng Luo

**Affiliations:** ^1^ Department of Oncology Zhujiang Hospital, The First School of Clinical Medicine, Southern Medical University; Donghai County People's Hospital (Affiliated Kangda College of Nanjing Medical University) Lianyungang China; ^2^ Department of Urology Changhai Hospital, Naval Medical University (Second Military Medical University) Shanghai China; ^3^ School of Stomatology Southern Medical University Guangzhou Guangdong China; ^4^ Department of Oncology Sichuan Provincial People's Hospital, University of Electronic Science and Technology of China Chengdu Sichuan China; ^5^ Department of Radiology Sichuan Provincial People's Hospital, University of Electronic Science and Technology of China Chengdu Sichuan China; ^6^ Department of Oncology, Zhujiang Hospital Southern Medical University Guangzhou Guangdong China; ^7^ Department of Neurosurgery Xiangya Hospital, Central South University Changsha Hunan China; ^8^ National Clinical Research Center for Geriatric Disorders Xiangya Hospital, Central South University Changsha Hunan China; ^9^ Cardio‐Cerebral Vascular Disease Prevention and Treatment Innovation Center Donghai County People's Hospital (Affiliated Kangda College of Nanjing Medical University) Lianyungang Jiangsu China; ^10^ Shantou University Medical College Shantou China; ^11^ Department of Microbiology, State Key Laboratory of Emerging Infectious Diseases Carol Yu Centre for Infection, School of Clinical Medicine, Li Ka Shing Faculty of Medicine, The University of Hong Kong Hong Kong China

**Keywords:** discrete‐time survival prediction, non–small cell lung cancer, spatial‐feature topology, spatial transcriptomics, tumour immune microenvironment, weakly supervised multi‐task MIL, whole‐slide imaging

## Abstract

**Background:**

Tumour‐node‐metastasis staging does not fully explain prognostic heterogeneity in non‐small cell lung cancer. We evaluated whether haematoxylin‐and‐eosin whole‐slide images could estimate histological subtype, pathological stage probabilities, survival risk and spatially grounded biological associations.

**Methods:**

SparseAGE‐MTL, a weakly supervised multi‐task multiple‐instance learning model with a shared projection‐topology encoder and endpoint‐specific heads, was trained and benchmarked in 954 The Cancer Genome Atlas cases using seven pathology feature spaces and 18 comparator models. External evaluation used 948 tissue‐microarray and 324 whole‐slide cases. Attention maps were co‐registered with 10x Visium spatial transcriptomics and integrated with bulk transcriptomics, immune‐infiltration estimates and ESTIMATE scores. Analyses included paired model comparisons, false‐discovery‐rate correction, Cox models, calibration assessment and decision curve analysis.

**Results:**

In the CONCH feature space, SparseAGE‐MTL achieved 93.73% accuracy, 98.19% area under the receiver‐operating‐characteristic curve and 93.08% F1‐score for adenocarcinoma/squamous cell carcinoma classification in internal benchmarking; external area‐under‐the‐curve values were approximately .91 and .82. Stage estimation had lower discrimination, with external overall area under the curve approximately .70 and cohort‐dependent calibration. Risk‐score‐defined groups differed in overall survival in both histological subtypes and showed similar external trends. High‐attention regions were enriched at tumour–stroma or tumour–immune interfaces and were associated with B‐cell, fibroblast, C1QC, COL1A1, epithelial–mesenchymal transition, metastasis and hypoxia signals. Higher risk cases showed malignant pathway activation, lower immune/stromal scores, higher tumour purity and subtype‐specific immune/stromal differences. Adding the risk score to the clinical model increased external pooled concordance index from approximately .620 to .672.

**Conclusions:**

In retrospective cohorts, SparseAGE‐MTL generated subtype‐classification, stage‐probability and survival‐risk outputs from routine pathology images. Subtype classification had higher numerical performance than stage estimation. Survival‐risk and attention outputs were associated with outcome and spatial/transcriptomic features, but prospective, treatment‐annotated validation is required before clinical use.

**Key points:**

- SparseAGE‐MTL is a weakly supervised multi‐task MIL framework that jointly performs NSCLC subtype classification, stage prediction, and survival risk estimation from routine H&E slides using only slide-level labels.
- The model achieves stable competitive performance across seven feature spaces and 18 comparators, with external validation demonstrating robust generalization to independent WSI and TMA cohorts.
- Attention hotspots co‐localize with tumor‐stroma/immune interfaces and spatial transcriptomic signatures of EMT, hypoxia, and C1QC/COL1A1 enrichment, providing biologically grounded interpretability.
- High‐risk groups exhibit activated malignant pathways, lower immune/stromal scores, higher tumor purity, and incremental prognostic value beyond clinical variables.

## Graphical abstract

SparseAGE‐MTL analyses routine haematoxylin and eosin pathology images from multicentre non‐small cell lung cancer cohorts to generate subtype‐classification, stage‐probability and survival‐risk outputs. Task‐specific attention maps and risk scores were integrated with spatial and bulk transcriptomics, linking high‐attention regions and high‐risk cases to tumour–stroma/immune interfaces, hypoxia/stress programmes, immune/stromal differences, and survival. The framework provides retrospective morphology‐molecular interpretation and requires prospective, treatment‐annotated validation before clinical use.

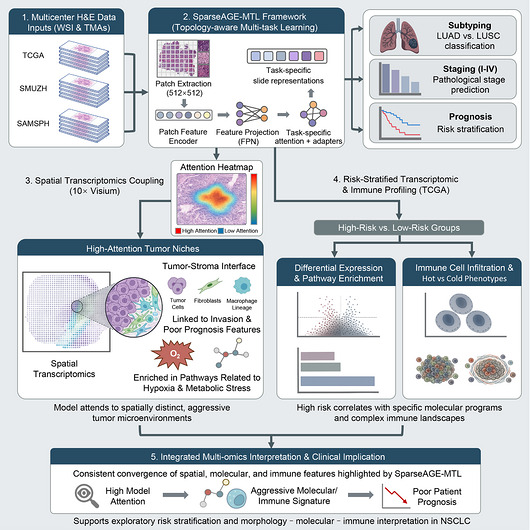

## 1 INTRODUCTION

Lung cancer is one of the malignancies with the highest incidence and mortality worldwide, and non‐small cell lung cancer (NSCLC) accounts for approximately 80%–85% of all lung cancer cases.^1^ Although diagnostic and therapeutic strategies for NSCLC have continued to improve, the overall 5‐year survival rate remains approximately 26.4%.^2^ For early‐stage resectable NSCLC, the TNM staging system remains the primary basis for determining surgical strategy and adjuvant treatment strategy.^3^ However, clinical evidence has shown that patients with the same TNM stage and similar treatments can still have marked differences in disease‐free survival (DFS) and overall survival (OS).^4^ In addition, the two major histological subtypes of NSCLC, lung adenocarcinoma (LUAD) and lung squamous cell carcinoma (LUSC), differ systematically in driver mutation profiles, metabolic pathways and immune status; these differences and the associated microenvironmental heterogeneity cannot be fully captured by TNM staging alone.^5^ Therefore, constructing a whole‐slide image (WSI)‐derived risk score from routine haematoxylin‐and‐eosin (H&E) WSIs may provide supplementary risk information for postoperative risk stratification, adjuvant therapy discussion, and surveillance planning. If this risk score can be further linked to spatially resolved molecular programmes, such as spatial transcriptomic signatures, and tumour immune microenvironment (TIME) phenotypes in an interpretable manner, morphology‐level risk phenotypes can be aligned with local immune‐stromal niches, hypoxia‐associated regions, tumour–stroma interfaces, and other spatial structures, thereby supporting the generation of subsequent treatment‐related biomarker hypotheses.

In recent years, NSCLC prediction models have expanded from conventional clinicopathological variables to radiomics, ctDNA, transcriptomics, proteomics, histopathology and other modalities,^6^, ^7^, ^8^, ^9^ providing diverse information sources for patient risk assessment. However, existing studies usually focus on a single data type or a single clinical task, such as subtype classification, mutation prediction or survival prediction. For the interrelated histological subtype, pathological stage and survival risk in NSCLC, systematic evaluation within a unified WSI‐based framework remains limited. Meanwhile, pathology image models in real‐world applications must address domain shifts arising from different institutions, slide‐preparation workflows and slide formats; in particular, when WSIs and tissue microarrays (TMAs) coexist, cross‐centre and cross‐slide‐type performance requires further validation.^10^, ^11^ Moreover, although attention heatmaps are widely used to display model‐attended regions, quantitative evidence remains insufficient regarding whether high‐attention regions correspond to specific molecular pathways, spatial transcriptomic states, or TIME phenotypes. Therefore, beyond existing NSCLC prediction models, there remains a need to establish a routine H&E WSI‐based analytical framework that jointly evaluates related but non‐equivalent clinical endpoints, including histological subtype, pathological stage and survival risk, tests generalisation in external cohorts, and clarifies the relationship between WSI‐derived risk scores and tumour molecular status and immune microenvironment.

TIME is closely associated with NSCLC progression, prognosis and immunotherapy response.^12^, ^13^ Based on immune cell infiltration, immune activation, immune exclusion and related features, TIME is often described as two relative states: hot tumour and cold tumour. The former is usually characterised by higher immune‐cell infiltration and immune activation, whereas the latter is more often accompanied by immune exclusion, low immune infiltration, or an immunosuppressive microenvironment and is associated with differences in immune checkpoint blockade response.^14^, ^15^, ^16^ However, LUAD and LUSC differ in driver alterations, metabolic programmes and immune contexts, and TIME characteristics may also vary across prognostic risk levels. Existing studies more commonly describe TIME from the perspective of transcriptomics, immunohistochemistry, or individual immune markers, whereas whether routine H&E WSI‐based morphology‐derived risk stratification reflects corresponding transcriptomic‐immune microenvironment features remains insufficiently investigated. Linking WSI‐derived risk scores to immune infiltration, stromal remodelling, and TIME‐related molecular patterns may therefore help explain the prognostic information captured by pathology image models from a biological‐context perspective.

Spatial transcriptomics provides a spatial analytical approach to these questions. This technology measures spatially resolved gene expression while preserving tissue architecture, thereby characterising cell‐type composition, functional states, and ligand–receptor interaction networks at local spatial scales.^17^ It can therefore be used to assess whether the high‐attention regions of pathology AI correspond to specific tumour niches, such as tumour‐dense regions, tumour–stroma/immune interfaces, hypoxia/stress‐related regions, or immune‐excluded regions. Studies have begun to explore the correspondence between deep learning attention maps and spatial transcriptomics,^18^ but systematic spatial interpretation of WSI‐derived risk scores, molecular pathways, and TIME phenotypes remains limited in NSCLC.

Against this background, we developed SparseAGE‐MTL, a weakly supervised joint multi‐task multiple‐instance learning (MIL) framework for NSCLC H&E WSIs, to evaluate NSCLC subtype classification, LUAD/LUSC pathological stage prediction, and survival risk estimation within a unified WSI‐based architecture. First, in the TCGA cohort, we systematically compared SparseAGE‐MTL, the non‐MTL comparator SparseAGE‐MIL, single‐task configurations, and representative MIL frameworks across feature spaces and endpoint‐level metrics, and analysed the impact of the Spatial‐Feature Topology Aggregator on patch‐level representations, *K* sensitivity, Top‐*K* topology spatial footprint, and attention concentration. We then selected the default feature space by integrating benchmark results and topology diagnostics and evaluated cross‐centre and cross‐slide‐type generalisation in two independent external cohorts, covering both WSIs and TMAs. Next, we co‐registered task‐specific attention heatmaps generated by SparseAGE‐MTL with 10x Visium spatial transcriptomics to analyse the biological context of high‐attention regions in terms of cell‐type composition, functional states and signalling pathways. Finally, using TCGA‐LUAD and TCGA‐LUSC bulk transcriptomics and external follow‐up data, we compared differential expression, pathway enrichment, multi‐algorithm immune infiltration, ESTIMATE scores and clinical utility between model‐derived high‐risk and low‐risk groups to evaluate the relationship between WSI‐derived risk score, molecular pathways, TIME phenotypes and clinical prognostic information.

## 2 METHODS

### 2.1 Participants and acquisition of H&E‐stained histopathology images

**FIGURE 1:**
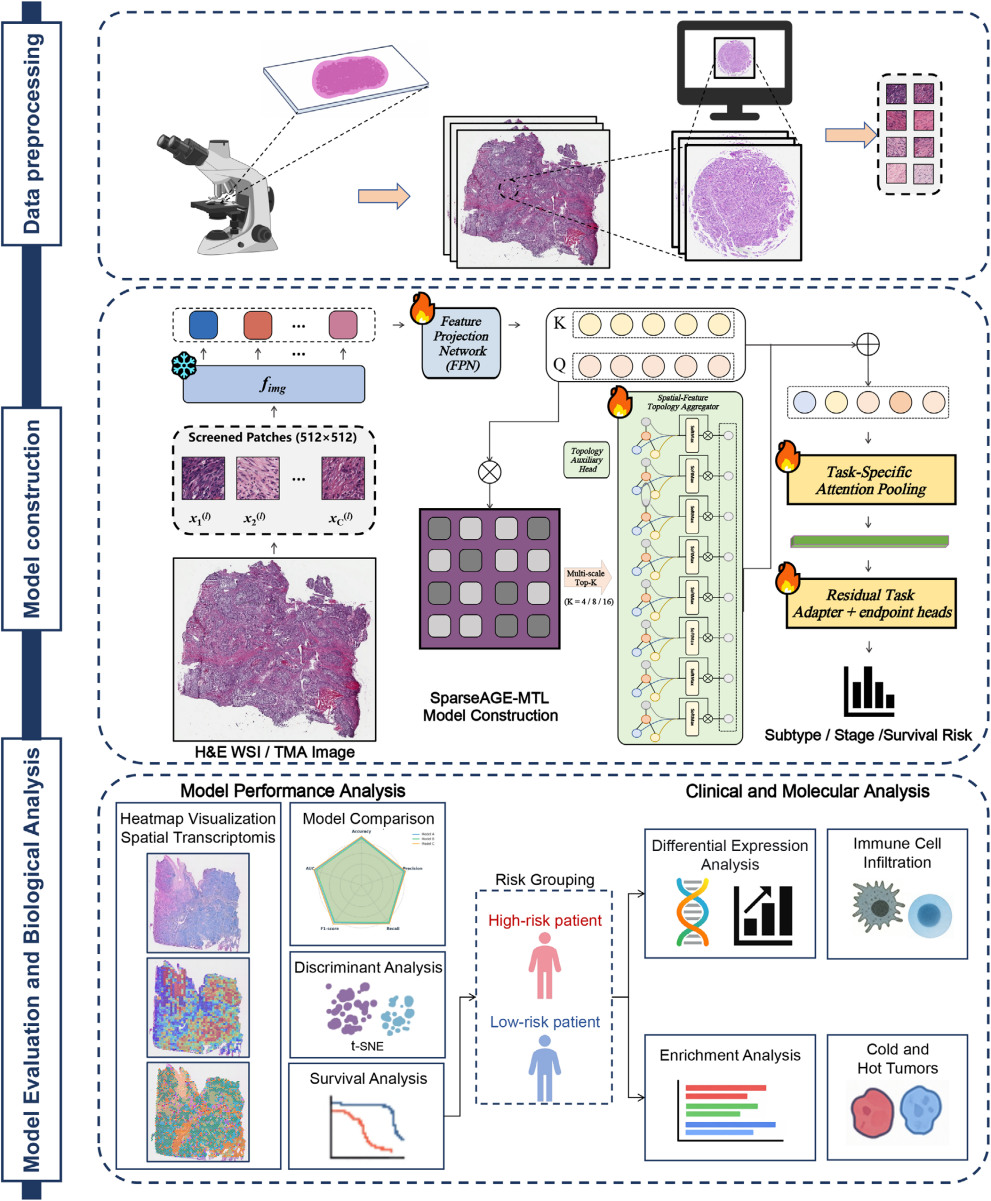
Schematic overview of the multi‐task SparseAGE‐MTL framework and multi‐omics biological interpretation. The study workflow comprises three primary phases: data preprocessing, model development and model evaluation integrated with biological interpretation. (Top) During the data preprocessing phase, H&E‐stained NSCLC WSIs and TMAs from multicentre cohorts were digitally scanned, background‐corrected, and subsequently partitioned into 512 × 512‐pixel non‐overlapping patches at 20× magnification to facilitate subsequent modelling. (Middle) In the model development phase, a pretrained feature extractor first encodes instance‐level features for each image patch, followed by nonlinear projection through the Feature Projection Network. The Spatial‐Feature Topology Aggregator then constructs sparse multi‐scale Top‐*K* neighbourhoods by integrating feature affinity with optional patch coordinates, pseudo‐histology cluster labels and cross‐cluster interface prior. Gated message passing and learnable scale weights generate topology‐regularised patch embeddings, which are subsequently summarised by task‐specific attention pooling and Residual Task Adapter modules. Endpoint‐specific output heads are used for NSCLC subtype classification, LUAD/LUSC stage prediction, and discrete‐time survival prediction, while a lightweight topology auxiliary head provides additional topology regularisation. (Bottom) During model evaluation and biological analysis, SparseAGE‐MTL was systematically benchmarked across seven pretrained feature spaces and 11 endpoint‐level metrics against 18 comparator models, including the non‐MTL comparator SparseAGE‐MIL and 17 representative non‐SparseAGE MIL baselines. Model evaluation further included overall mean rank, paired fold‐level difference, feature‐extractor contribution, feature‐extractor sensitivity, Spatial‐Feature Topology Aggregator diagnostics, attention concentration analysis, and patient‐level statistical comparison. Survival stratification was evaluated using Kaplan–Meier analysis and *C*‐index. Task‐specific attention heatmaps from SparseAGE‐MTL were co‐registered with 10x Visium spatial transcriptomics to analyse cell‐type composition, functional states, and spatial niche features in high‐attention regions. Based on slide‐level risk scores, patients were divided into high‐risk and low‐risk groups and further analysed using bulk transcriptomic differential expression analysis, pathway enrichment analysis, multi‐algorithm immune infiltration profiling, ESTIMATE‐based hot/cold tumour phenotype analysis and clinical utility analysis, thereby establishing an interpretive framework covering morphology, spatial transcriptomics, molecular programmes, TIME and clinical prognostic value.

**FIGURE 2:**
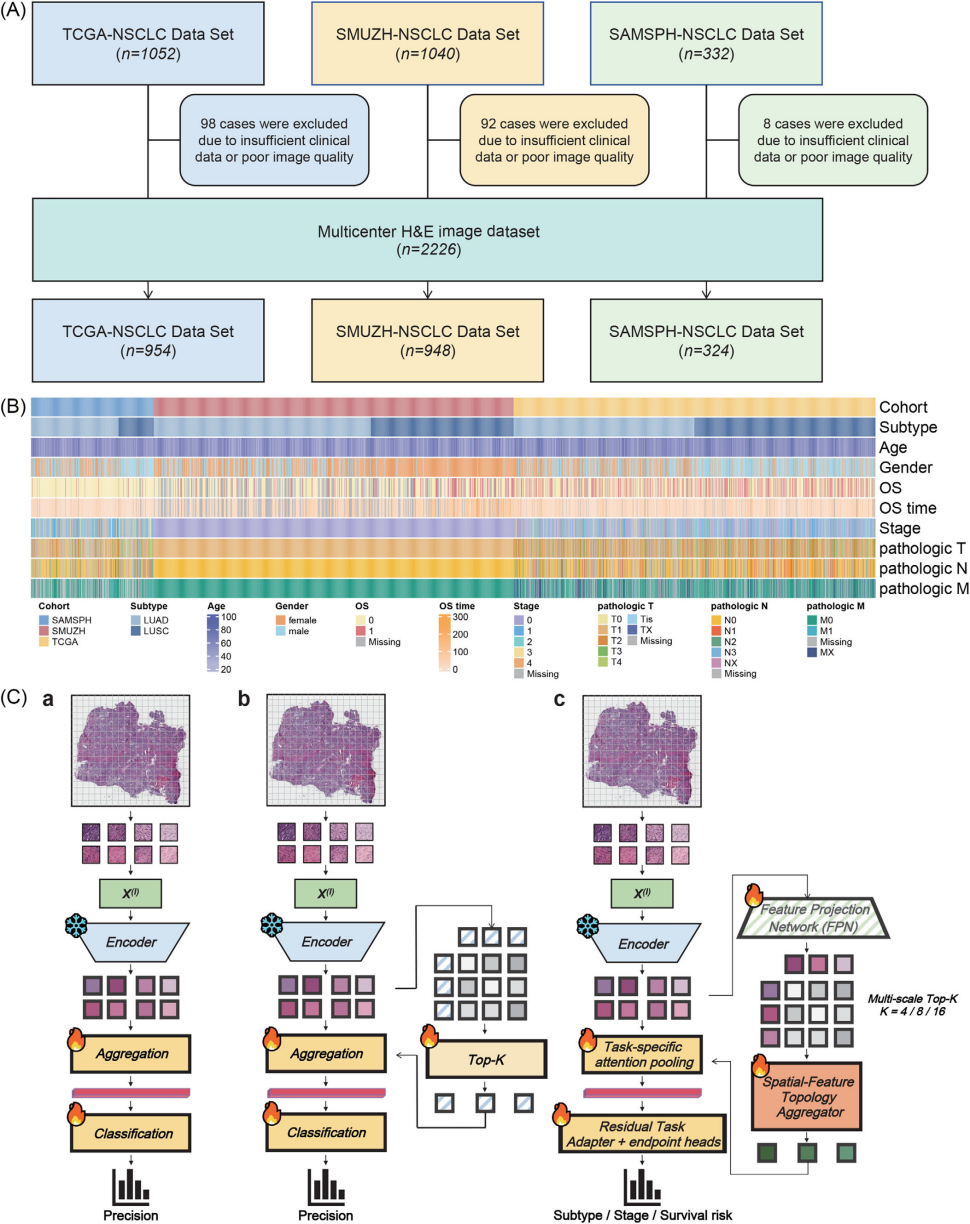
Multicentre NSCLC cohort construction and comparison of SparseAGE‐MTL model architectures. (A) Flowchart of patient inclusion and screening. Three major cohorts—TCGA‐NSCLC (*n* = 1052), SMUZH‐NSCLC (*n* = 1040) and SAMSPH‐NSCLC (*n* = 332)—were collected. After applying exclusion criteria (e.g., incomplete clinical information and poor image quality), a total of 2226 high‐quality whole‐slide images were retained, comprising 954 cases from TCGA‐NSCLC, 948 cases from SMUZH‐NSCLC and 324 cases from SAMSPH‐NSCLC, constituting the multicentre WSI dataset for this study. (B) Distribution of clinical and pathological features of the merged cohorts. The heatmap illustrates the distribution of variables across the cohorts, including subtype (LUAD/LUSC), age, gender, OS, OS duration, stage and pathological T/N/M status. (C) Schematic representation and comparison of model architectures. Panel a illustrates the traditional MIL framework, in which instance features are extracted after WSI patching and directly aggregated for bag‐level prediction. Panel b illustrates an augmented Top‐*K* MIL variant that selects high‐confidence patches, but lacks explicit spatial‐feature topology modelling. Panel c depicts the proposed SparseAGE‐MTL framework: instance embeddings are first projected by the Feature Projection Network, then modelled by the Spatial‐Feature Topology Aggregator through sparse multi‐scale Top‐*K* neighbourhoods, gated message passing and scale‐weighted message fusion. The resulting topology‐regularised patch embeddings are summarised by task‐specific attention pooling and Residual Task Adapter modules before entering endpoint‐specific output heads.

The overall workflow of this study encompassed data preprocessing, model construction and multilevel biological analyses integrating both spatial and bulk transcriptomics, as detailed in Figure 1. This study included publicly available data from TCGA and independent, institutional cases from Southern Medical University Zhujiang Hospital (SMUZH) and Sichuan Academy of Medical Sciences & Sichuan Provincial People's Hospital (SAMSPH). The inclusion criteria were as follows: pathologically confirmed and explicitly subtyped as either LUAD or LUSC; no prior history of radiotherapy, chemotherapy or other systemic antitumour treatments prior to the initial diagnosis; availability of diagnostic‐quality H&E WSIs or TMAs; and availability of essential clinical information (including at least age, sex and pathological subtype, with a subset of cases also providing TNM staging, overall survival and survival status). Patients were excluded if they presented with distant metastasis, concurrent secondary malignancies, suboptimal slide quality, completely missing clinical data, or unconfirmable pathological labels. OS was defined as the interval from the date of surgery to either death or the date of the last follow‐up. The patient screening flowchart is depicted in Figure 2A.

The TCGA cohort comprised the TCGA‐LUAD and TCGA‐LUSC datasets, with resection specimens routinely processed as formalin‐fixed paraffin‐embedded (FFPE) blocks and stained with H&E. These public multi‐institutional WSIs retained the original scanning and image‐acquisition settings from the contributing institutions, and were scanned at either 20× or 40× magnification, thereby introducing institution‐level acquisition heterogeneity. A total of 477 LUAD and 477 LUSC cases were included, yielding one WSI per case.^19^, ^20^ The SMUZH cohort comprised H&E‐stained tissue microarray (TMA) slides, representing 572 LUAD and 376 LUSC cases diagnosed between 2001 and 2013; these slides were digitised at 20× magnification using a 3DHISTECH Pannoramic SCAN II digital slide scanner. The SAMSPH cohort consisted of formalin‐fixed paraffin‐embedded (FFPE) whole‐section slides acquired from 2018 to 2022, encompassing 231 LUAD and 93 LUSC cases; these slides were scanned at 20× magnification using a KFBIO KF‐PRO‐005 digital pathology slide scanner. Collectively, the study data showed acquisition‐domain heterogeneity in source institution, slide format, scanner type, magnification and acquisition protocol. All cases were reviewed by senior thoracic pathologists in accordance with the 2021 WHO Classification of Thoracic Tumors,^21^ with only slide‐level labels being provided. The detailed clinical characteristics are summarised in Figure 2B; the aforementioned H&E images were subsequently utilised for downstream preprocessing and analysis.

### 2.2 SparseAGE‐MTL

A classical MIL workflow typically consists of three steps: patch‐level feature extraction, slide‐level feature aggregation, and bag‐level prediction.^22^ In this study, each WSI or TMA core was treated as a bag composed of multiple image patches. Following previous computational pathology MIL workflows,^11^, ^23^, ^24^ we divided tissue regions into non‐overlapping 512 × 512 patches at 20× magnification and used CLAM‐based tissue filtering to remove non‐informative regions such as blank background.^11^

We then used seven pretrained feature extractors to extract patch‐level embeddings offline, including CONCH, Virchow2, H‐optimus‐1, UNI2‐h, PLIP, UNI and ResNet‐50.^25^, ^26^, ^27^, ^28^, ^29^, ^30^, ^31^, ^32^ ResNet‐50 served as a general natural‐image CNN baseline; PLIP and CONCH represented publicly available pathology vision‐language foundation models; and UNI, UNI2‐h, Virchow2 and H‐optimus‐1 represented large‐scale pathology foundation models from different stages of development. Each pretrained feature extractor was evaluated as an independent feature space; embeddings from different backbones were not concatenated, fused or ensembled within the same forward pass. Because different foundation models produce raw embeddings with different dimensions, SparseAGE‐MTL uses a feature extractor‐agnostic Feature Projection Network to map all patch embeddings to a unified shared latent space, with a default embedding dimension of D=512, before passing them into the Spatial‐Feature Topology Aggregator for topology‐aware sparse aggregation.

SparseAGE‐MTL was designed as a weakly supervised joint multi‐task MIL framework with spatial‐topology regularisation; its architecture schematic is shown in Figure 2C. The model consists of a shared Feature Projection Network and Spatial‐Feature Topology Aggregator, forming a projection‐topology encoder, followed by task‐specific attention pooling, Residual Task Adapters, endpoint‐specific output heads and a lightweight topology auxiliary head. First, the Feature Projection Network performs nonlinear projection of offline patch embeddings in the current feature space. The Spatial‐Feature Topology Aggregator then constructs sparse multi‐scale Top‐*K* neighbourhoods based on feature affinity and, when available, incorporates a spatial‐topology prior jointly formed by patch coordinates, pseudo‐histology cluster labels and a cross‐cluster interface prior. In the default joint multi‐task setting, the Top‐*K* set is K={4,8,16}. At each Top‐*K* scale, the model performs gated message passing over selected neighbour patches, fuses multi‐scale messages through learnable scale weights, and finally obtains topology‐regularised patch embeddings through residual normalisation.

In joint multi‐task training, the shared projection‐topology encoder simultaneously serves three endpoint families: LUAD/LUSC subtype classification, pathological stage prediction and discrete‐time survival prediction.^33^ The target of subtype classification was the slide‐level histological subtype, namely LUAD versus LUSC. The target of stage prediction was pathological Stage I–IV, encoded in the model as an ordinal‐aware classification target from 0 to 3; subsequent evaluation reported stage prediction metrics separately for LUAD and LUSC. The target of survival prediction comprised OS time and censoring status. OS time was discretised into fixed time bins in the training split and used to train the discrete‐time survival head; survival probabilities output by the model were further converted to a slide‐level risk score, and subsequent evaluation was performed separately in LUAD and LUSC. These endpoints were optimised simultaneously in the default joint multi‐task setting. Each endpoint used an independent task‐specific attention pooling module, Residual Task Adapter and endpoint‐specific output head, whereas the Feature Projection Network and Spatial‐Feature Topology Aggregator were shared across endpoints. For samples missing an endpoint label, the model ignored the loss for that endpoint through missing‐label masking, allowing the sample to update the shared encoder based on other valid labels or topology targets. The total loss function consisted of subtype cross‐entropy, ordinal‐aware stage loss, discrete‐time survival negative log‐likelihood and topology auxiliary loss, with homoscedastic uncertainty weighting used by default for multi‐task loss weighting.^34^ SparseAGE‐MIL was retained as the non‐MTL comparator to evaluate performance changes of joint multi‐task learning relative to the non‐MTL setting; single‐task configurations were used only for ablation and task‐specific comparison experiments. The mathematical definition of SparseAGE‐MTL is provided in Supporting Methods Section 1.

### 2.3 Model benchmarking and paired statistical comparison

Using the TCGA cohort, we systematically evaluated the benchmark performance of SparseAGE‐MTL in NSCLC subtype classification, pathological stage prediction and survival risk estimation. Model evaluation included seven pretrained feature extractors, namely CONCH, Virchow2, H‐optimus‐1, UNI2‐h, PLIP, UNI and ResNet‐50.^25^, ^26^, ^27^, ^28^, ^29^, ^30^, ^31^, ^32^ SparseAGE‐MTL was compared with 18 comparator models, including the non‐MTL comparator SparseAGE‐MIL and 17 representative non‐SparseAGE MIL baselines: Mamba‐MIL, TKA‐MIL, WiKG, RRT‐MIL, MHIM‐MIL, HMKGN, ACMIL, Patch‐GCN, Trans‐MIL, DTFD‐MIL, HIGT, CLAM, DS‐MIL, CAMIL, AB‐MIL, SCMIL and IB‐MIL.^11^, ^23^, ^24^, ^35^, ^36^, ^37^, ^38^, ^39^, ^40^, ^41^, ^42^, ^43^, ^44^, ^45^, ^46^, ^47^, ^48^ SparseAGE‐MIL was used as a non‐MTL comparator to evaluate changes associated with joint multi‐task learning relative to the non‐MTL setting; the remaining models were collectively referred to as non‐SparseAGE baselines. All models used the same slide‐level labels, feature files, data folds and evaluation metrics, and were evaluated in the TCGA cohort using matched five‐fold cross‐validation.^11^, ^49^, ^50^ All comparator configurations, fixed/default settings, selection metrics and external validation usage are listed in Table S5.

For cross‐model benchmark comparison, we constructed a task‐wise normalised performance matrix. This matrix covered 19 models and 77 feature‐endpoint‐metric settings, corresponding to combinations of seven pretrained feature extractors and 11 higher‐is‐better endpoint‐level metrics. For each setting, model performance was min–max normalised as follows:

$$\;s_{m,j}^{\mathrm{norm}}=(s_{m,j}-\min_{m}s_{m,j})/(\max_{m}s_{m,j}-\min_{m}s_{m,j})$$

where $s_{m,j}$ denotes the mean cross‐validation performance of model m under setting j. If all models had identical values within a setting, the denominator was set to 1. All models were then ranked in descending order within each setting, and the overall mean rank of each model was calculated:

$$\;{\overset{-}{r}}_{m}=77^{-1}\sum_{j\;=1}^{77}r_{m,j}$$

where a lower ${\overset{-}{r}}_{m}$ indicates a better overall ranking. For each model–feature–endpoint–metric combination, we retained the raw Fold 1–5 results and reported the mean, SD and bootstrap 95% confidence interval (CI).

To evaluate paired fold‐level differences between SparseAGE‐MTL and non‐SparseAGE baselines, we selected the non‐SparseAGE baseline with the highest mean performance within each feature–endpoint–metric setting and computed the paired difference based on matched five‐fold results:

$$\;\Delta_{f,e,k}=s_{\mathrm{target},f,e,k}\;-s_{\mathrm{best}\;\mathrm{baseline},f,e,k}$$

where f denotes the feature extractor, e denotes the endpoint‐level metric, k denotes the cross‐validation fold, and the target model was either SparseAGE‐MTL or SparseAGE‐MIL. Paired differences were summarised using the mean, SD, bootstrap 95% CI, exact sign‐flip test *p‐value*, and Benjamini–Hochberg FDR‐adjusted *Q* value.

### 2.4 Spatial‐Feature Topology Aggregator diagnostics and attention concentration analysis

To evaluate the effect of the Spatial‐Feature Topology Aggregator on patch‐level representations, we selected representative WSIs in the NSCLC subtype classification task and extracted patch‐level embeddings using the ResNet‐50, PLIP, UNI and CONCH feature spaces. For each feature space, we compared raw patch embeddings with topology‐regularised embeddings updated by the Spatial‐Feature Topology Aggregator. All embeddings were standardised and mapped to two dimensions using t‐SNE.^51^ In the visualisation, tumour patches and non‐tumour/normal patches were distinguished according to pathological region annotation or corresponding tissue labels. To quantitatively characterise changes in embedding distribution, we calculated intra‐class distance, inter‐class distance and separation ratio between tumour patches and non‐tumour/normal patches, and drew the 80% density contour of tumour patches using bivariate kernel density estimation.

To evaluate the impact of the hyperparameter K in single‐scale Top‐*K* aggregation on model performance, we additionally constructed a single‐scale diagnostic setting and trained models on the TCGA dataset using CONCH and PLIP feature spaces. We compared accuracy, AUC, F1‐score, precision and recall under different K values. Tested K values included 1, 2, 3, 4, 5, 6, 10, 20, 30, 40, 50 and 100. In this analysis, K=6 was used as the single‐scale diagnostic reference. Except for K, all training settings, data folds, labels, feature files and evaluation metrics were kept consistent. This *K* sensitivity analysis was used only to evaluate sensitivity to Top‐*K* neighbourhood size; the default joint multi‐task setting of the main model still used the multi‐scale Top‐*K* set K={4,8,16}.

To analyse whether the Top‐*K* topology learned in the single‐scale diagnostic setting primarily connected adjacent patches or also included long‐range dependencies, we performed spatial neighbourhood analysis on representative WSIs from the TCGA cohort. For each slide, we recorded the grid coordinates $\left(x_{i},y_{i}\right)$ obtained for each patch during tiling, and extracted the Top‐*K* neighbour indices generated by the Spatial‐Feature Topology Aggregator under the K=6 setting. For source patch i and its Top‐*K* neighbour j, grid‐based L1 distance was defined as $d_{ij}=|x_{i}-x_{j}\left|+\right|y_{i}-y_{j}|$. We then summarised the distance distribution of all (i,j) pairs and calculated the proportions of immediate grid neighbours, local neighbourhoods and long‐range neighbours. Immediate grid neighbours were defined as d=1, local neighbourhoods as d=2−3 and long‐range neighbours as d≥4. Distances were further divided into five bins, d=1, d=2−3, d=4−7, d=8−15 and d≥16, and a detailed histogram for d≤10 was additionally summarised to characterise the spatial footprint of the learned implicit graph.

To evaluate the spatial concentration of attention weights within WSIs, we extracted patch‐level attention scores from the survival head and normalised them as slide‐level attention distributions. We calculated the Gini coefficient, the effective number of attended patches and the top 1% attention mass of the attention distribution. The effective number of attended patches was defined as $N_{\mathrm{eff}\;}=1/\sum_{i}p_{i}^{2}$, where $p_{i}$ denotes the normalised patch‐level attention weight. The top‐1% attention mass was defined as the cumulative attention mass of patches ranked in the top 1% by attention weight. The attention heatmap and the original H&E image were then magnified in the same region to examine the spatial correspondence between high‐attention regions and local histomorphology.

### 2.5 Feature‐extractor contribution and default feature space selection

After completing cross‐model benchmark comparison and Spatial‐Feature Topology Aggregator diagnostics, we further quantified the overall performance of the seven pretrained feature extractors to determine the default feature space for subsequent external validation, attention analysis and biological interpretation. The endpoint‐level metrics included accuracy, AUC and F1‐score for NSCLC subtype classification; accuracy, AUC and F1‐score for LUAD stage prediction; accuracy, AUC and F1‐score for LUSC stage prediction; and C‐index for LUAD and LUSC survival prediction. For each feature extractor, we first summarised its five‐fold mean performance across all models and endpoint‐level metrics and calculated the global mean score: $G_{f}={\left(MN\right)}^{-1}\;\sum_{m=1}^{M}\sum_{e=1}^{N}s_{f,m,e}$, where $s_{f,m,e}$ denotes the mean performance corresponding to feature extractor f, model m and endpoint‐level metric e; M=19 denotes the number of models, and N=11 denotes the number of endpoint‐level metrics. To reduce the influence of absolute scale differences across endpoints on feature comparison, we further performed min–max normalisation across feature extractors within each endpoint to obtain the endpoint‐normalised score: $z_{f,e}=(s_{f,e}-\min_{f}s_{f,e})/(\max_{f}s_{f,e}-\min_{f}s_{f,e})$. If all feature extractors had identical values within an endpoint, the denominator was set to 1. Endpoint‐normalised scores were then summarised separately for SparseAGE‐MTL and SparseAGE‐MIL, and mean values and 95% CIs were estimated from fold‐level normalised scores. To distinguish the relative contributions of MIL architecture and feature extractor to performance differences, we performed two complementary comparisons: a feature‐fixed architecture comparison, in which MIL architectures were compared after fixing the CONCH feature space; and an architecture‐fixed feature comparison, in which feature extractors were compared after fixing SparseAGE‐MIL. Finally, approximate variance contribution analysis based on endpoint‐normalised benchmark scores was used to approximately decompose total variation into four components: MIL architecture, feature extractor, endpoint and residual/interactions. Selection of the default feature space jointly considered the global mean score, endpoint‐normalised feature‐extractor sensitivity, topology diagnostics and *K* sensitivity. External validation was used subsequently to evaluate cross‐cohort generalisation under the selected default feature space and was not involved in feature space selection. Detailed methods are provided in Supporting Methods Section 2.

### 2.6 Multicenter validation of model generalisability

For the NSCLC subtyping and LUAD/LUSC tumour staging tasks, the default feature space selected as described in Section 2.5 was used for instance‐level feature extraction. The TCGA‐NSCLC dataset was randomly partitioned into a training set (80%) and an internal validation set (20%), while the SMUZH‐NSCLC and SAMSPH‐NSCLC cohorts from independent medical centres were introduced as external validation sets to evaluate the model's generalisability across diverse institutions, acquisition conditions and data distributions. In the joint multi‐task setting of SparseAGE‐MTL, labels for different endpoints were encoded separately, and missing labels were handled through missing‐label masking during loss calculation. For each reported endpoint‐specific metric, only samples with complete and valid labels for that endpoint were included in the evaluation.

In the LUAD and LUSC survival prediction tasks, the same default feature space was used. The TCGA‐NSCLC cohort served as the sole source of training samples, while the SMUZH‐NSCLC and SAMSPH‐NSCLC cohorts were treated as independent external validation cohorts. The WSI‐derived risk score was generated from the survival prediction head. For each tumour subtype, the risk cutoff was determined in the TCGA training cohort and then fixed when applied to internal and external validation cohorts. Only patients with complete OS time and censoring status were included in survival model training and evaluation. Survival performance was evaluated using both risk‐group separation and discrimination metrics. Kaplan–Meier curves and two‐sided log‐rank tests were used to compare high‐risk and low‐risk groups. Hazard ratios (HRs) and 95% CIs were estimated using Cox proportional hazards models. Harrell's *C*‐index was used to quantify survival discrimination. Cohort‐specific HRs, HR 95% CIs, log‐rank *p*‐values and *C*‐index values were reported for internal and external cohorts.

### 2.7 Joint spatial analysis of spatial transcriptomics and attention heatmaps

This study utilised the 10x Genomics Visium platform to acquire spatial transcriptomics data from pathologically confirmed LUSC and LUAD samples (https://www.10xgenomics.com/datasets/human‐lung‐cancer‐ffpe‐2‐standard https://www.10xgenomics.com/datasets/ffpe‐human‐lung‐cancer‐data‐with‐human‐immuno‐oncology‐profiling‐panel‐and‐custom‐add‐on‐1‐standard) to annotate cell types and functional regions, verify the reliability of the annotations, and analyse peritumoural tissues. Raw data were processed via Space Ranger for spatial alignment and expression matrix construction, followed by quality control based on gene count and mitochondrial proportion thresholds before normalisation and highly variable gene selection. Based on a curated marker gene set derived from published single‐cell transcriptomic studies of normal human lung and lung cancer tissues,^52^ each spot was scored for Cell Subtype to annotate its predominant cell type, which was further integrated into higher level Cell Lineages. Detailed gene lists and mapping relationships are provided in Table S1. Concurrently, using functional gene sets provided by the CancerSEA database,^53^ with the detailed gene list in Table S2, functional module scores were calculated for each spot to annotate its primary Functional State, thereby characterising the distribution patterns of different cell types and functional states within the tissue microenvironment. Cell Subtypes, Cell Lineages and Functional States were then mapped back to the spatial coordinates of tissue slides and visualised by multicolour labelling to intuitively display the spatial distribution patterns and dynamic transitions of functional states within tissue architecture. The reliability of these annotations was evaluated by selecting typical Cell Subtype‐specific genes for comparative spatial expression pattern analyses and nonlinear dimensionality reduction visualisation. Furthermore, to quantitatively delineate microenvironmental gradients among the tumour core, margin and peripheral tissues from the perspectives of both physical distance and molecular features, we constructed a neighbourhood search strategy based on Visium spatial coordinates to partition peritumoural regions. On this basis, spatial visualisation and quantitative comparisons of key immune‐ and stroma‐related genes were performed across different spatial partitions, such as C1QC,^54^ expressed by the monocyte‐macrophage lineage, especially tumour‐associated macrophages, and COL1A1,^55^ highly expressed by fibroblasts and cancer‐associated fibroblasts (CAFs). In addition, the Mann–Whitney *U* test was used to assess expression differences in peritumoural regions relative to the tumour core and distal tissues to dissect the molecular characteristics of the tumour‐margin microenvironment.

After SparseAGE‐MTL training was completed, we extracted instance‐level attention weights from the task‐specific attention pooling module of the corresponding endpoint and mapped them back to homologous WSI coordinates to generate attention heatmaps. For survival‐oriented spatial interpretation, survival‐head attention was preferentially used; for subtype‐oriented interpretation, subtype‐head attention was used. High‐attention regions were then co‐registered with spatial distributions of cell‐type composition, Cell Lineage and Functional State inferred from spatial transcriptomics to evaluate the consistency between model attention patterns and spatial molecular features. We further focused on LUSC cell‐enriched regions, stratified tumour spots into high‐priority tumour spots and low‐priority tumour spots according to attention intensity, and performed differential expression analysis, GO enrichment analysis, KEGG pathway analysis and GSEA within the LUSC cell subset to characterise invasion, EMT, hypoxia, metabolic reprogramming and immune‐related pathways associated with high‐attention regions. Detailed methods are provided in Supporting Methods Section 3.

To quantitatively evaluate the correspondence between attention hotspots and spatial transcriptomics‐derived tissue features, we mapped patch‐level attention scores to Visium spot coordinates and retained only spots with valid attention assignment, spot‐level ST annotation and spatial region labels. Based on pathologist‐guided review and transcriptomic annotation, spots were classified into four spatial regions: tumour‐dense, tumour–stroma/immune interface, stroma/immune‐rich and other tissue. Attention score distributions among different spatial regions were compared using the Kruskal–Wallis test, followed by FDR‐adjusted pairwise comparisons when needed. Top‐attention spots were defined as the top 10% of spots ranked by attention score. For each spatial region, the enrichment ratio was defined as $F\;E_{r}=O_{r}\;/E_{r}$, where $O_{r}$ is the observed top‐attention spot count in region r, $E_{r}=T(n_{r}/N)$ is the expected count, T is the total number of top‐attention spots, $n_{r}$ is the number of valid spots in region r and N is the total number of valid spots. Top‐attention enrichment was evaluated using Fisher's exact test with Benjamini–Hochberg FDR correction. Correlations between attention scores and ST‐derived cell‐type scores, functional state scores and local neighbourhood composition features were calculated using Spearman correlation, with FDR correction applied to all tested features.

### 2.8 Transcriptomic, immune and clinical utility analyses of model‐derived risk groups

Based on transcriptomic sequencing data from the TCGA‐LUAD and TCGA‐LUSC cohorts, this study systematically profiled the molecular characteristics of patients stratified by different risk levels. According to the established risk scoring model, both types of NSCLC patients were categorised into high‐risk and low‐risk groups. Differential expression analysis was then performed using the edgeR package,^56^ with thresholds set at |log2FC| > 1 and *p* < .05, to ensure the statistical and biological reliability of the identified differentially expressed genes. On this basis, GO enrichment analysis^57^ was conducted on significantly differentially expressed genes to systematically elucidate biological processes, molecular functions and signal transduction pathways closely associated with patient risk stratification.

To evaluate TIME differences between model‐derived high‐risk and low‐risk groups, immune/stromal cell infiltration scores were calculated from TCGA‐LUAD and TCGA‐LUSC bulk transcriptomic data. Six immune infiltration algorithms, including TIMER, CIBERSORT, QUANTISEQ, MCP‐counter, xCell and EPIC, were used to estimate relative infiltration levels of different immune or stromal cell populations.^58^ Analyses were performed separately within each tumour subtype. For each cell‐type × algorithm combination, we compared the original immune‐infiltration scores between high‐risk and low‐risk groups and recorded sample size, mean, median, high‐minus‐low difference, standardised mean difference, raw *p*‐value and FDR *q* value for both groups. Two‐group comparisons used the Mann–Whitney *U* test; multiple testing used Benjamini–Hochberg FDR correction, with correction performed uniformly across all tested cell‐type × algorithm combinations within each tumour subtype.^59^ Heatmap visualisation used row‐wise *Z*‐score‐normalised infiltration scores only to display relative patterns across patient samples and was not used for statistical testing. FDR significance annotations were defined as follows: ∗ indicates FDRq<.05, ∗∗ indicates FDRq<.01, ∗∗∗ indicates FDRq<.001, and NS indicates non‐significance after FDR correction.

To evaluate the incremental value of the WSI‐derived risk score in clinical prognostic modelling, we constructed Cox proportional hazards models using cases with complete clinical variables, survival time, survival status, and WSI‐derived risk score. The clinical model included clinical variables such as age, sex, pathological subtype and pathological stage. The AI model included the standardised SparseAGE‐MTL risk score. The combined model included both clinical variables and the SparseAGE‐MTL risk score. Continuous variables were standardised or converted according to prespecified scales: age was modelled per 10‐year change, and risk score was modelled per 1 SD change. Models were fitted in the TCGA cohort and evaluated in external cohorts for discrimination, calibration and potential clinical utility. Discrimination was evaluated using *C*‐index and time‐dependent AUCs; calibration was evaluated using 1‐, 3‐ and 5‐year calibration curves; and potential clinical utility was evaluated using 3‐year decision curve analysis.^60^

### 2.9 Statistical analysis

All statistical analyses used two‐sided tests, with *p* < .05 as the nominal significance threshold. For classification tasks, including subtype classification and stage prediction, model performance was evaluated using accuracy, precision, recall, F1‐score and ROC‐AUC; multiclass stage prediction used one‐versus‐rest ROC‐AUC. Classification calibration was assessed using reliability diagrams, Brier score and expected calibration error (ECE). For binary subtype classification, calibration was evaluated using the predicted probability of the positive class. For multiclass pathological stage prediction, one‐versus‐rest calibration curves were generated for each stage class, and class‐level Brier score and ECE were summarised across stage classes. The Brier score was calculated as $\mathrm{Brier}=N^{-1}\;\sum_{i\;=1}^{N}{\left({\hat{p}}_{i}-y_{i}\right)}^{2}$. ECE was calculated as $\mathrm{ECE}=\sum_{b=1}^{B}\frac{\left|I_{b}\right|}{N}\;|\mathrm{acc}(I_{b})\;-\;\mathrm{conf}(I_{b})|$, where $I_{b}$ denotes samples assigned to calibration bin b. Because external stage‐specific sample sizes were limited and stage labels were imbalanced, stage calibration was interpreted as a diagnostic analysis rather than as primary evidence of clinical applicability.^61^, ^62^ TCGA benchmark performance was summarised from matched five‐fold cross‐validation results, and mean performance and 95% CI were reported. For fold‐level paired model comparison, the non‐SparseAGE baseline with the highest mean performance was independently selected within each feature‐endpoint‐metric setting, and the paired difference between the target model and the best baseline was calculated using matched folds; the 95% CI of the mean paired difference was estimated using bootstrap resampling.^63^ For patient‐level AUC comparison in classification tasks, paired AUC differences were evaluated using the DeLong test.^64^ For survival prediction, patients were divided into high‐risk and low‐risk groups according to the median risk score defined in the training set, and this cutoff was applied unchanged to validation cohorts. Overall survival differences were evaluated using Kaplan–Meier curves and log‐rank tests, with effect sizes reported as HRs and 95% CIs. Patient‐level *C*‐index differences were estimated using bootstrap resampling to obtain 95% CIs. For spatial transcriptomics regional comparisons, continuous variables were compared using the Mann–Whitney *U* test or Kruskal–Wallis test, and FDR correction was used for multiple‐group or multiple‐feature comparisons. For immune/stromal cell infiltration analysis, original immune‐infiltration scores were compared between high‐risk and low‐risk groups using the Mann–Whitney *U* test, followed by Benjamini–Hochberg FDR correction across all cell‐type × algorithm combinations within each tumour subtype. FDRq<.05 was considered FDR‐adjusted statistical significance. Raw *p*‐value, FDR *q* value, high‐minus‐low mean difference and standardised mean difference were also reported. Attention enrichment analysis used Fisher's exact test and FDR correction. Attention‐feature associations used Spearman correlation with FDR correction across multiple features. For bulk transcriptomics, differential expression analysis between risk groups used edgeR, followed by GO/KEGG enrichment analysis and GSEA; Benjamini–Hochberg FDR correction was used for analyses requiring multiple testing. For clinical utility analysis, Cox proportional hazards models were used to estimate HRs and 95% CIs, and time‐dependent AUCs, C‐index, calibration curves and decision curve analysis were used to compare the prognostic performance and potential clinical utility of the clinical model, AI model and combined model.

## 3 RESULTS

### 3.1 SparseAGE‐MTL shows stable competitive benchmark performance across feature spaces and MIL architectures

**FIGURE 3:**
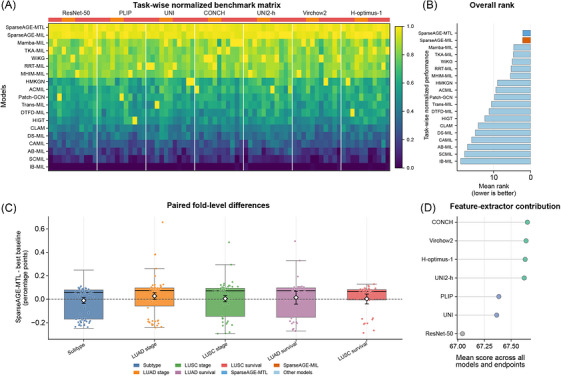
Benchmark‐style performance summary of SparseAGE‐MTL across feature extractors, endpoint‐level metrics and comparator MIL architectures. (A) Task‐wise normalised benchmark matrix showing the relative performance of 19 models across 77 feature‐endpoint‐metric settings. The 77 settings consist of seven pretrained feature extractors and 11 higher‐is‐better endpoint‐level metrics, covering subtype classification, LUAD stage prediction, LUSC stage prediction, LUAD survival prediction and LUSC survival prediction. Model performance within each setting was min–max normalised across all models, with brighter colours indicating higher relative performance. Rows are ordered by overall mean rank, and the top annotation bar indicates endpoint categories. (B) Overall mean rank of each model across the 77 feature‐endpoint‐metric settings. A lower mean rank indicates a better overall ranking. SparseAGE‐MTL and the non‐MTL comparator SparseAGE‐MIL are highlighted with different colours, while the remaining models are grouped as other non‐SparseAGE MIL baselines. (C) Paired fold‐level differences between SparseAGE‐MTL and the best non‐SparseAGE baseline. The best non‐SparseAGE baseline was independently selected within each feature‐endpoint‐metric setting. Each point represents a matched cross‐validation fold‐level difference; boxplots show the distribution of paired differences within each endpoint category; the dashed line indicates zero difference; and white diamonds with error bars indicate the bootstrap mean estimate and its 95% CI. Positive values indicate that SparseAGE‐MTL outperformed the corresponding best non‐SparseAGE baseline. (D) Feature‐extractor contribution analysis summarised by the global mean score of each feature extractor across all models and endpoint‐level metrics. The *x*‐axis indicates the mean score across all models and endpoints, and each point indicates one pretrained feature extractor.

SparseAGE‐MTL showed stable competitive benchmark performance across multiple feature spaces, endpoint‐level metrics and comparator models (Figure 3A,B). In the systematic benchmark covering seven pretrained feature extractors, 11 endpoint‐level metrics and 19 models, we ranked models by performance within each feature‐endpoint‐metric setting and calculated the overall mean rank across 77 settings. SparseAGE‐MTL achieved the lowest overall mean rank (2.11), indicating an average ranking of approximately second across all settings. The non‐MTL comparator SparseAGE‐MIL had an overall mean rank of 2.16; together, the two models constituted the top‐ranked SparseAGE family. In contrast, strong non‐SparseAGE baselines such as Mamba‐MIL, TKA‐MIL, WiKG, RRT‐MIL and MHIM‐MIL had relatively lower mean ranks. In the CONCH feature space, SparseAGE‐MTL achieved 93.73% accuracy, 98.19% AUC and 93.08% F1‐score for LUAD/LUSC subtype classification; 55.68% accuracy, 69.19% AUC and 50.82% F1‐score for LUAD stage prediction; 54.11% accuracy, 62.10% AUC and 48.60% F1‐score for LUSC stage prediction; and *C*‐indices of 64.22% and 59.38% for LUAD and LUSC survival prediction, respectively. CONCH‐based five‐fold mean estimates and 95% CIs are shown in Figure S1. Full fold‐level values, means, SDs, bootstrap 95% CIs and ranks for all model‐feature‐endpoint‐metric combinations are provided in Table S6.

We then evaluated fold‐level paired differences between SparseAGE‐MTL and the best non‐SparseAGE baseline selected within the same feature extractor, endpoint, metric and split setting (Figure 3C). The paired differences were generally close to zero or slightly above zero, with variable effect sizes across endpoint categories. Exact sign‐flip tests and Benjamini–Hochberg FDR correction did not support interpreting these paired differences as universal statistically supported improvements across all settings. Therefore, the benchmark supports cross‐feature and cross‐endpoint ranking stability of SparseAGE‐MTL rather than a large margin over every comparator model. Complete paired differences, SDs, bootstrap 95% CIs, exact sign‐flip *p*‐values and FDR‐adjusted *Q* values for SparseAGE‐MTL and SparseAGE‐MIL are reported in Table S7. SparseAGE‐MIL paired diagnostics are shown in Figure S2 as an architecture‐level ablation rather than as the primary SparseAGE‐MTL result.

### 3.2 Spatial‐Feature Topology Aggregator diagnostics and attention concentration analysis

**FIGURE 4:**
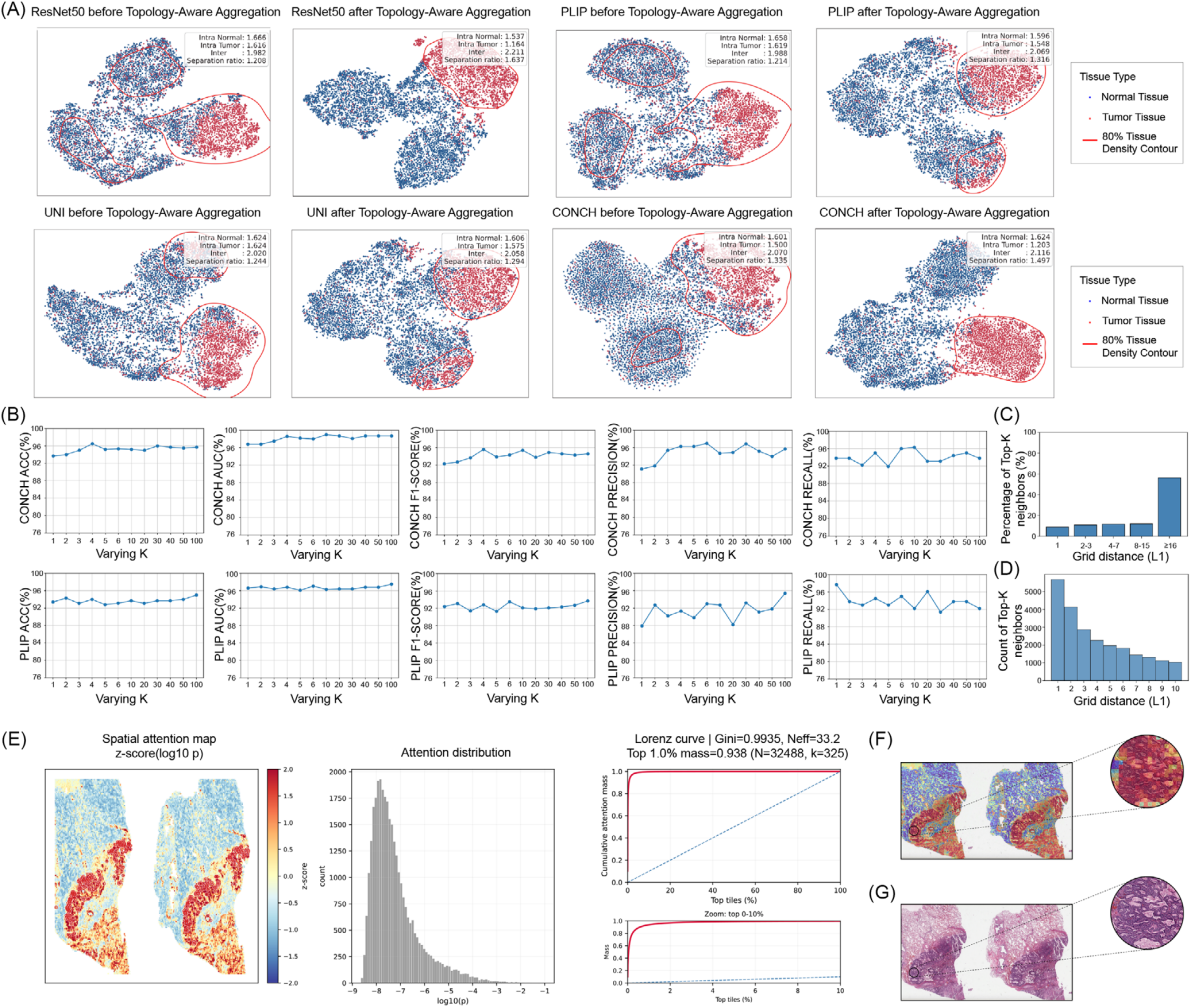
Spatial‐Feature Topology Aggregator diagnostics, *K* sensitivity, learned Top‐*K* topology and attention concentration analysis. (A) t‐SNE visualisation of patch‐level instance embeddings across four feature spaces before and after the Spatial‐Feature Topology Aggregator. ResNet‐50, PLIP, UNI and CONCH feature spaces are shown separately. Red points indicate tumour patches, and blue points indicate non‐tumour/normal patches. Red density contours indicate the 80% high‐density range of tumour patches. Quantitative summaries include intra‐class distance, inter‐class distance and separation ratio. (B) Impact of varying K values on model performance under a single‐scale diagnostic setting using CONCH and PLIP feature spaces. The evaluated metrics include accuracy, AUC, F1‐score, precision and recall. Tested K values include 1, 2, 3, 4, 5, 6, 10, 20, 30, 40, 50 and 100. The K=6 setting was used as the diagnostic reference for panels C and D, whereas the default joint multi‐task model used the multi‐scale Top‐*K* set K={4,8,16}. (C) Binned distribution of grid‐based L1 distances between each patch and its Top six neighbours in a representative TCGA WSI under the K=6 diagnostic setting. Bars show the percentage of Top‐*K* neighbour pairs in each distance bin, including d=1, d=2−3, d=4−7, d=8−15 and d≥16. (D) Histogram of Top‐*K* neighbour distances restricted to d≤10 under the K=6 diagnostic setting, showing the detailed distribution of short‐ and intermediate‐range neighbour connections. (E) Attention concentration analysis in a representative WSI. The panel shows the attention heatmap, normalised attention distribution and Lorenz curve of patch‐level attention weights. Summary statistics include Gini coefficient, effective number of attended patches, top 1% attention mass and total number of valid patches. (F and G) Zoom‐in visualisation of the high‐attention region. Panel F shows the enlarged attention heatmap, and panel G shows the corresponding enlarged original H&E image from the same region.

To evaluate the effect of the Spatial‐Feature Topology Aggregator on patch‐level representations, we compared the t‐SNE distributions of raw patch embeddings and topology‐regularised embeddings across the ResNet‐50, PLIP, UNI and CONCH feature spaces (Figure 4A). In all four feature spaces, tumour patches and non‐tumour/normal patches showed a clearer tendency toward separation after topology aggregation, accompanied by reduced overlap between the tumour 80% density contour and non‐tumour/normal high‐density regions. These results suggest that the Spatial‐Feature Topology Aggregator can alter the patch‐level embedding distribution.

*K* sensitivity analysis showed that accuracy, AUC, F1‐score, precision and recall fluctuated only slightly with K values in the CONCH and PLIP feature spaces (Figure 4B). In the single‐scale diagnostic setting, K=6 achieved optimal or near‐optimal performance in most evaluation metrics and was therefore used as the reference setting for Top‐*K* topology spatial footprint analysis in Figure 4C,D. Smaller K values may limit contextual information aggregation, whereas excessively large K values may introduce redundant or weakly related patches. The subsequent main‐model experiments still used the multi‐scale Top‐*K* set K={4,8,16} rather than a single K=6 setting.

Top‐*K* topology spatial footprint analysis further showed that the learned Top‐*K* neighbours were not restricted to immediate grid adjacency (Figure 4C,D). After binning by grid‐based L1 distance, immediate grid neighbours with d=1 accounted for only a small proportion, local neighbourhoods with d=2−3 were also not the dominant component, and long‐range neighbours with d≥4 constituted a larger proportion (Figure 4C). In the detailed histogram restricted to d≤10, neighbour distances displayed a continuous distribution from local to more distant ranges rather than concentrating at d=1 (Figure 4D). These results indicate that the implicit graph learned by the Spatial‐Feature Topology Aggregator does not merely reproduce tiling grid adjacency but can establish patch‐level connections across local regions.

Attention concentration analysis showed that endpoint‐specific attention weights in SparseAGE‐MTL were highly concentrated within WSIs (Figure 4E). In representative slides, high‐attention regions in the attention heatmap covered only a limited number of patches, and the Lorenz curve together with top 1% attention mass showed that attention weights were mainly concentrated in a small subset of patches. Further zoom‐in visualisation showed spatial correspondence between high‐attention heatmap regions and local tumour morphology and tumour–stroma interface structures in the original H&E image (Figure 4F,G). These results provided a visual basis for subsequent spatial transcriptomics co‐registration and high‐attention niche analysis.

### 3.3 Default feature space selection based on benchmark performance and topology diagnostics

In the feature‐extractor contribution analysis, CONCH had the highest global mean score across all models and endpoint‐level metrics, followed by Virchow2, H‐optimus‐1 and UNI2‐h, whereas PLIP, UNI and ResNet‐50 had relatively lower overall scores (Figure 3D). Because the global mean score summarises raw mean performance and may be affected by differences in absolute numerical ranges across endpoints, we further performed feature‐extractor sensitivity analysis using endpoint‐normalised scores. The results showed that CONCH and Virchow2 were among the leading feature spaces in both SparseAGE‐MTL and the non‐MTL comparator SparseAGE‐MIL, whereas ResNet‐50 had relatively weaker endpoint‐normalised performance (Figure S3). Approximate variance contribution analysis showed that the MIL architecture explained the largest proportion of variation in endpoint‐normalised benchmark performance, followed by the feature extractor, whereas endpoint and residual/interactions contributed relatively little (Figure S4).

Integrating the topology diagnostics in Figure 4, the CONCH feature space showed stable performance in t‐SNE representation visualisation, *K* sensitivity, and subsequent attention‐based interpretation. Considering cross‐model benchmark performance, endpoint‐normalised feature‐extractor sensitivity, topology diagnostics and *K* sensitivity together, we selected CONCH as the default feature space for subsequent generalisation studies, attention analysis and biological interpretation. External validation was then used to evaluate cross‐cohort generalisation under this default feature space and was not involved in feature space selection.

Patient‐level statistical analyses showed that AUC differences and *C*‐index differences between SparseAGE‐MIL and the best non‐SparseAGE baseline were generally small, and most bootstrap 95% CIs crossed zero (Figures S5 and S6). Therefore, these analyses are more suitable for showing that the SparseAGE family is comparable to or leading among strong non‐SparseAGE baselines, rather than supporting the interpretation that differences in a single setting represent universally significant superiority.

### 3.4 Cross‐cohort validation of SparseAGE‐MTL across NSCLC tasks

#### 3.4.1 Subtype classification across institutions and slide types

**FIGURE 5:**
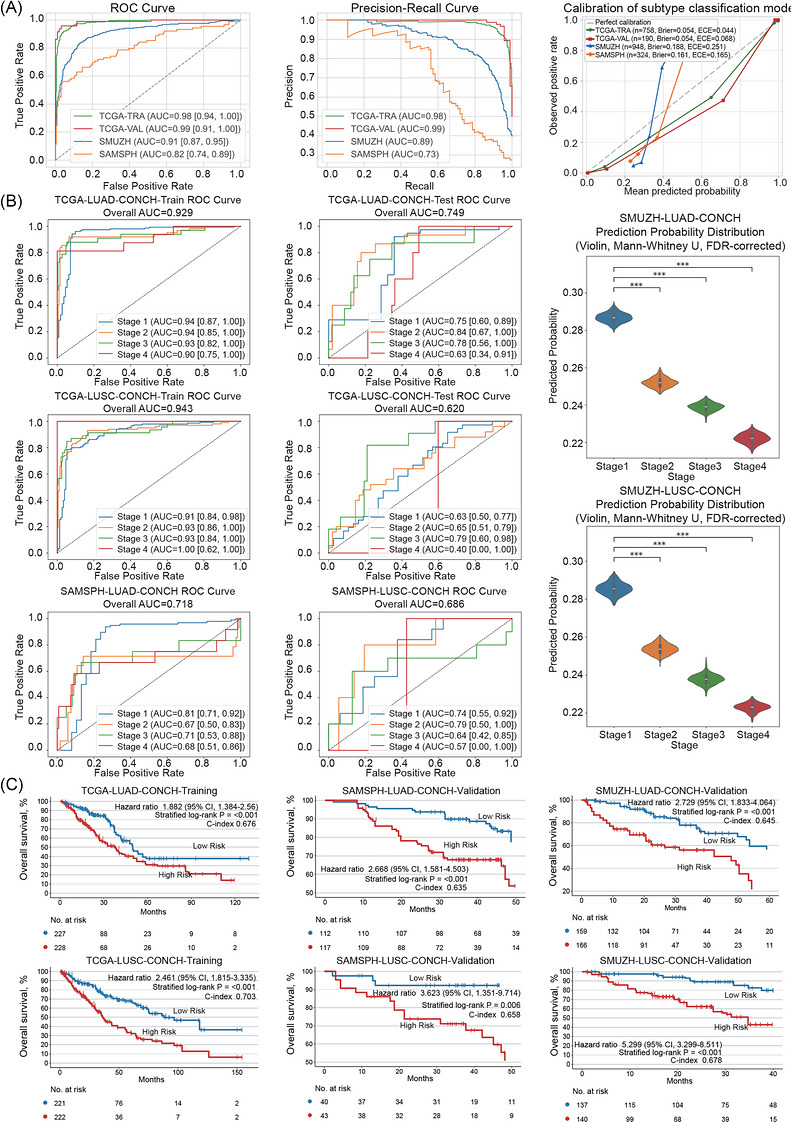
Cross‐cohort validation of SparseAGE‐MTL for subtype classification, stage prediction and survival risk stratification. (A) External validation of LUAD/LUSC subtype classification. Receiver operating characteristic curves, precision‐recall curves and calibration diagnostics are shown for the TCGA training set, TCGA internal validation set, external SMUZH TMA cohort and external SAMSPH WSI cohort. The calibration panel reports Brier score and ECE for each cohort. (B) Cross‐cohort validation of LUAD and LUSC stage prediction. Multiclass ROC curves are shown for TCGA‐LUAD, TCGA‐LUSC and external SAMSPH cohorts. Violin plots summarise predicted stage‐related probabilities in the external SMUZH cohort. (C) Kaplan–Meier survival curves for model‐derived high‐risk and low‐risk groups in LUAD and LUSC. Risk groups were defined using the median risk score from the training set and then applied to internal and external validation cohorts.

Using the TCGA‐NSCLC dataset as the source domain and CONCH as the default feature space, SparseAGE‐MTL maintained high discriminability for LUAD/LUSC subtype classification in both internal and external cohorts (Figure 5A, Figure S7A, B). ROC‐AUCs in the TCGA training set and internal validation set were close to .98 and .99, respectively, while the ROC‐AUCs in the external SMUZH TMA cohort and SAMSPH WSI cohort were approximately .91 and .82, respectively. Calibration diagnostics showed that Brier score and ECE were relatively low in the TCGA training set and internal validation set. In contrast, calibration curves in external cohorts deviated from the perfect calibration line, and ECE values in the SMUZH TMA cohort and SAMSPH WSI cohort were approximately .25 and .17, respectively. Therefore, predicted probabilities from SparseAGE‐MTL should not be interpreted as fully calibrated diagnostic probabilities; however, its subtype discrimination remained acceptable under cross‐centre and cross‐slide‐type domain shifts.

#### 3.4.2 External stage prediction shows limited discrimination in LUAD and LUSC

Training and validation curves for the CONCH‐based LUAD and LUSC stage‐prediction models are shown in Figure S7C. Stage confusion matrices and prediction‐confidence distributions showed that incorrect predictions were usually accompanied by lower or more dispersed maximum class probabilities, suggesting that model output prediction confidence reflected predictive uncertainty to some extent (Figure S7D). For the external SMUZH cohort, we further used violin plots to show predicted probabilities across patients with different pathological stages. The results showed observable probability distribution differences between Stage I and higher stage cases (Figure 5B). To further assess probability calibration of stage prediction, we performed one‐versus‐rest reliability diagrams, Brier score and ECE analyses in the TCGA training set, TCGA test set and external SAMSPH cohort (Figure S7D). Overall, the calibration pattern in the training set was more stable, whereas validation and external cohorts showed some cohort‐dependent variation. Considering that pathological stage prediction is affected by class imbalance, stage‐specific sample size and heterogeneity in morphology‐stage correspondence, these calibration diagnostics are better interpreted as auxiliary evaluations rather than final confirmation of absolute stage probabilities. Overall, SparseAGE‐MTL can provide morphology‐derived stage probability estimates to assist in characterising tumour morphological progression patterns, but its outputs should still be interpreted together with standard pathological TNM staging information.

#### 3.4.3 External validation of survival risk stratification in LUAD and LUSC

In the discrete‐time survival prediction task, SparseAGE‐MTL provided survival risk stratification for LUAD and LUSC based on H&E‐stained slides (Figure 5C). In the TCGA training cohort, high‐risk and low‐risk groups defined by the median risk score showed distinct OS distributions in both subtypes (LUAD HR approximately 1.9, *C*‐index = .676; LUSC HR approximately 2.5, *C*‐index = .703). In the SMUZH‐NSCLC TMA cohort and SAMSPH‐NSCLC WSI cohort, high‐risk groups defined using the same threshold also showed a trend toward poorer survival outcomes. We further evaluated time‐specific survival calibration at representative time points in LUAD and LUSC (Figure S7E). Calibration curves showed that predicted survival probabilities generally reflected the direction of change in observed survival estimates, but cohort‐ and timepoint‐dependent variation was present in some external cohort–timepoint combinations, particularly when event numbers were limited.

### 3.5 Spatial coupling of transcriptomic architecture and model attention in NSCLC

To systematically analyse the coupling characteristics between tumour spatial transcriptomic architecture and SparseAGE‐MTL attention weights in NSCLC, this section uses one LUSC sample as a representative example. This sample showed marked spatial heterogeneity and reflected typical NSCLC histological features. The corresponding LUAD spatial transcriptomics analysis is shown in Figure S9.

#### 3.5.1 Spatial landscapes of cell types and functional states revealed by Visium ST

**FIGURE 6:**
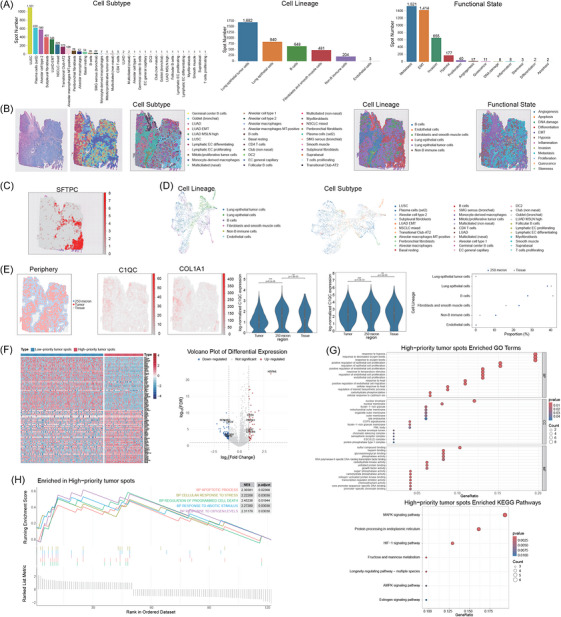
Spatial transcriptomic characterisation of SparseAGE‐MTL attention hotspots in a representative LUSC section. (A) Bar plots summarising spot‐level annotations, including fine‐grained cell subtype, aggregated cell lineage and dominant functional state derived from spatial transcriptomics. (B) Spatial visualisation of the matched H&E image, SparseAGE‐MTL attention heatmap, cell subtype map, cell lineage map and functional state map. (C) Spatial expression pattern of SFTPC in the representative LUSC section. (D) UMAP visualisation of spatial transcriptomics spots coloured by cell lineage and cell subtype annotations. (E) Spatial and quantitative analysis of tumour–stroma/immune interface features. The panel includes periphery annotation, C1QC and COL1A1 expression maps, and lineage‐level composition summaries across spatial regions. (F) Differential expression analysis between high‐attention and low‐attention tumour spots, including heatmap and volcano plot summaries. (G) GO and KEGG enrichment analysis of genes upregulated in high‐priority tumour spots. Dot size indicates gene count or GeneRatio, and colour indicates adjusted enrichment significance according to the figure scale. (H) GSEA visualisation of pathway‐level differences between high‐priority and low‐priority tumour spots. Enrichment curves, ranked gene list positions, NES and adjusted *p*‐values are shown for representative stress‐ and survival‐related gene sets.

Utilising the 10x Visium data, we performed cell‐subtype annotation at the spot resolution via marker‐gene scoring and subsequently mapped these results onto the matched H&E‐stained slide (Figure 6A,B): LUSC tumour cells (1101 spots) formed a large, continuous spatial distribution, constituting the predominant component of the tissue section. Interspersed within this region were various other subtypes—including plasma cells (Set 2), alveolar Type 2 cells, subpleural fibroblasts, LUAD EMT and mixed NSCLC cells—indicating a tumour microenvironment highly infiltrated by abundant immune and stromal elements. Subsequently, these cell subtypes were integrated into six major cell lineages based on predefined mapping relationships. Lung epithelial tumour cells (1682 spots) and non‐malignant lung epithelial cells (840 spots) localised primarily to the central and right regions of the section. B cells (648 spots), along with fibroblasts and smooth muscle cells (481 spots), exhibited a band‐like distribution along the tumour–stroma interface. Non‐B immune cells (204 spots) were relatively scattered, whereas endothelial cells were sparsely detected (three spots), reflecting a weak vascular signal within this specific sample.

Furthermore, we conducted functional state annotations using the CancerSEA gene sets (Figure 6A,B). Signatures for metastasis (1521 spots) and EMT (1414 spots) exhibited substantial spatial overlap within the main tumour body. Invasion‐related signals (655 spots) and hypoxia‐related signals (177 spots) were also observed, with invasion signals more broadly distributed and hypoxia signals showing a more focal spatial pattern. Proliferation, angiogenesis, DNA damage, inflammation and other functional states were detected only in smaller subsets of spots.

To validate the accuracy of the cell annotations derived from marker‐gene scoring, we evaluated the results across three dimensions: spatial expression, low‐dimensional embedding and subpopulation clustering. Taking SFTPC (a marker gene for alveolar Type II cells) as an example, its regions of high spatial expression strongly overlapped with spots annotated as alveolar Type 2 cells; specifically, SFTPC demonstrated strong spatial expression at the bottom of the section and within partial glandular structures, thereby supporting the reliability of the alveolar epithelium‐related labels (Figure 6C). Upon projecting all spots into a UMAP low‐dimensional space (Figure 6D), the six major cell lineages formed relatively distinct and well‐separated clusters. Following further refinement into more than 30 distinct cell subtypes, the annotation strategy maintained discriminative power for both high‐ and low‐abundance cell populations. Collectively, this concordant evidence substantiates the reliability of our cell‐type annotation methodology.

Centring on ‘lung epithelial tumour cells’, we partitioned the samples into the tumour core (Tumour), the 250‐µm peritumoural region (250 µm) and the distal background tissue (Tissue) (Figure 6E). Spatial distribution analysis revealed that the 250‐µm peritumoural region formed a continuous annulus around the tumour islands, enveloping the tumour–stroma interface in a mosaic pattern, whereas the distal Tissue was predominantly located at the slide edges and surrounding the cavities. This suggests that this partitioning provides a geometrically rational representation of the ‘tumour core–proximal margin–distal background’ gradient structure. Within this spatial neighbourhood framework, C1QC and COL1A1 were significantly enriched at the tumour margin with partially overlapping spatial patterns, indicating that the infiltration of C1QC^+^ monocytes/macrophages, collagen deposition and stromal remodelling exhibit heightened activity at this tumour–stroma interface. Cellular composition analysis further demonstrated that, compared with the distal Tissue, the proportions of B cells, non‐B immune cells, fibroblasts and smooth muscle cells were elevated in the 250‐µm region, whereas the proportion of lung epithelial cells was reduced. These findings indicate that the 250‐µm peritumoural region in lung squamous cell carcinoma constitutes an immune‐stromal interface microenvironment distinct from both the tumour core and distal tissues.

#### 3.5.2 Alignment of SparseAGE‐MTL attention hotspots with spatial microenvironment features

After registration between the H&E WSI and matched Visium section, SparseAGE‐MTL attention weights showed a non‐uniform spatial distribution in the LUSC tissue section (Figure 6B). Visual overlay showed that high‐attention regions were not uniformly distributed across all tumour or stromal regions, but appeared more frequently in tumour‐dense areas and in selected tumour–stroma/immune interface regions. Attention hotspots in some marginal regions were spatially close to mixed tumour‐immune‐stromal niches, including regions with relatively prominent macrophage‐related and fibroblast‐associated signals. This suggests that the model may not rely solely on tumour cell density but may also capture tumour‐intrinsic morphology and local microenvironmental context. CancerSEA‐based functional annotation further showed that high‐attention regions spatially corresponded to invasion‐associated or stress‐related states, including EMT, metastasis, hypoxia and inflammation‐related signatures. This observation is consistent with the C1QC/COL1A1 enrichment in the aforementioned 250‐micron peritumoural region,^52^ suggesting that SparseAGE‐MTL attention hotspots may be associated with hypoxia‐immune‐stroma‐associated tumour niches.

To avoid relying only on qualitative visual inspection, we further performed spot‐level quantitative validation. Specifically, patch‐level attention scores were mapped to Visium spots with valid ST annotations and pathologist‐guided spatial region labels, and normalised attention score distributions were compared across spatial regions (Figure S8). Among 3615 valid mapped spots, normalised attention scores differed across tumour‐dense, tumour‐stroma/immune interface, other tissue and stroma/immune‐rich regions (Kruskal–Wallis H=345.8, $p\;=1.2\;\times \;10^{-74}$). Tumour‐dense regions had the highest median normalised attention (median = .341), followed by tumour‐stroma/immune interface regions (median = .302), whereas other tissue (median = .270) and stroma/immune‐rich regions (median = .230) were lower. Further top‐attention enrichment analysis showed that top 10% attention spots were enriched in tumour‐dense regions (FE=1.62, 96/362 top‐attention spots, $\mathrm{FDR}\;q\;=5.4\;\times \;10^{-7}$) and also enriched in tumour‐stroma/immune interface regions (FE=1.21, 205/362 top‐attention spots, $\mathrm{FDR}\;q\;=1.6\;\times \;10^{-4}$). In contrast, top 10% attention spots were not enriched in stroma/immune‐rich regions (FE=.07, 3/362 top‐attention spots, FDRq=1.000). In feature‐level correlation analysis, attention score was positively correlated with cell‐cycle score (Spearman ρ=.49, $\mathrm{FDR}\;q\;=2.5\;\times \;10^{-218}$), DNA‐repair score (ρ=.44, $\mathrm{FDR}\;q\;=5.6\;\times \;10^{-166}$), and proliferative macrophage‐related score (ρ=.38, $\mathrm{FDR}\;q\;=2.0\;\times \;10^{-126}$), whereas B‐cell‐ and plasma‐cell‐related features were negatively correlated with attention score (Figure S8).

Therefore, qualitative spatial overlay and quantitative spot‐level analysis jointly support the association of SparseAGE‐MTL attention hotspots with tumour‐intrinsic activity and selected tumour‐stroma/immune interface features. This analysis was based on a single matched ST section and is therefore more appropriately interpreted as spatially anchored validation of attention patterns rather than causal evidence of the model prediction mechanism.

#### 3.5.3 LUSC subset composition underlies differential attention hotspots in the tumour niche

Within the LUSC tumour region, high‐ and low‐priority tumour spots exhibited systematic transcriptomic differences (Figure 6F). Differential gene expression analysis revealed that high‐attention regions characteristically displayed molecular signatures indicative of enhanced invasion and adaptability. Specifically, genes intrinsically linked to the stress response, cell survival, signal transduction regulation and tumour progression (e.g., HSPA6, PROX1, CITED4, CIART and CD55) were significantly upregulated.^65^, ^66^, ^67^, ^68^, ^69^ Conversely, genes associated with cell adhesion, axon guidance and the maintenance of metabolic homeostasis—including SEMA6C, LMO4, MEGF6 and the HIGD family—were significantly downregulated.^70^, ^71^, ^72^, ^73^ Functional enrichment analysis provided pathway‐level quantitative support for the spatial interpretation of high‐priority tumour spots (Figure 6G,H). For GO Biological Process terms, upregulated genes in high‐priority tumour spots were enriched in response to hypoxia, response to decreased oxygen levels, response to cellular stress, positive regulation of epithelial cell proliferation, and positive regulation of endothelial cell proliferation, with all displayed representative terms passing FDR‐adjusted enrichment thresholds (Figure 6G). At the KEGG pathway level, representative enriched pathways included the MAPK signalling pathway (GeneRatio = .20; Count = 6), protein processing in the endoplasmic reticulum (GeneRatio = .17; Count = 5), HIF‐1 signalling pathway (GeneRatio = .13; Count = 4), AMPK signalling pathway, and estrogen signalling pathway indicating concurrent stress signalling, hypoxia adaptation, protein‐processing stress and metabolic reprogramming (Figure 6G). GSEA further confirmed positive enrichment of stress‐ and survival‐related functional modules in high‐priority tumour spots (Figure 6H), including REGULATION OF PROGRAMMED CELL DEATH (NES = 2.45, adjusted *p* = .019), APOPTOTIC PROCESS (NES = 2.30, adjusted *p* = .024), CELLULAR RESPONSE TO STRESS (NES = 2.22, adjusted *p* = .030), RESPONSE TO ABIOTIC STIMULUS (NES = 2.27, adjusted *p* = .030), and RESPONSE TO OXYGEN LEVELS (NES = 2.31, adjusted *p* = .030). These quantitative enrichment results support the interpretation that high‐attention LUSC regions are associated with hypoxia‐related stress adaptation and survival‐oriented pathway activity. Low‐attention tumour spot analyses are provided in Figure S10.

### 3.6 Molecular and immune microenvironment differences between high‐ and low‐risk groups

In both LUAD and LUSC, the high‐risk groups stratified by SparseAGE‐MTL exhibited hallmarks of malignant progression at the molecular level, including metabolic reprogramming, oxidative stress/ferroptosis regulation, epithelial remodelling and multi‐pathway signalling activation. At the cellular and microenvironmental levels, they demonstrated a ‘cold tumour’ or ‘immune exclusion’ phenotype characterised by decreased lymphocyte infiltration, enrichment of immunosuppressive cells and elevated tumour purity.

#### 3.6.1 Identification of differentially expressed genes between high‐ and low‐risk groups

**FIGURE 7:**
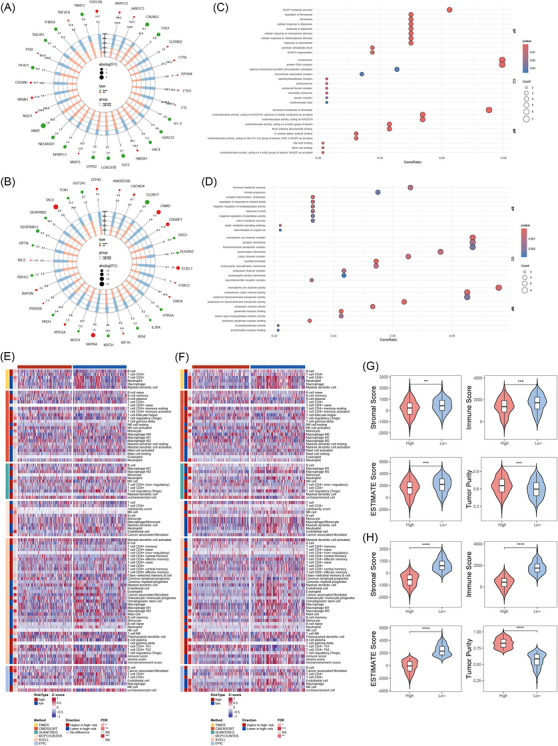
Transcriptomic and immune microenvironment characterisation of model‐derived high‐risk and low‐risk groups in LUAD and LUSC. (A and B) Circular visualisation of differentially expressed genes between high‐risk and low‐risk groups in LUAD (A) and LUSC (B). Genes are arranged by genomic location or annotation category according to the plotting scheme, and colours indicate the direction and magnitude of differential expression. (C and D) GO enrichment analysis of differentially expressed genes in LUAD (C) and LUSC (D). Enriched terms are grouped by biological process, cellular component, or molecular function according to the figure annotation. (E and F) Sample‐level heatmaps of immune/stromal cell infiltration scores in LUAD (E) and LUSC (F). Infiltration scores were estimated using TIMER, CIBERSORT, QUANTISEQ, MCP‐counter, xCell and EPIC. Rows represent immune or stromal cell populations, and columns represent individual patients ordered by model‐derived risk group. Heatmap colours represent row‐wise *Z*‐score‐normalised infiltration scores and are used only for visualisation. Between‐group comparisons used original immune‐infiltration scores with Mann–Whitney *U* tests, followed by Benjamini–Hochberg FDR correction across all tested cell‐type × algorithm combinations within each tumour subtype. Row annotations indicate FDR‐adjusted significance levels and the direction in the high‐risk group relative to the low‐risk group. ∗, ∗∗ and ∗∗∗ indicate FDRq<.05, FDRq<.01 and FDRq<.001, respectively; NS indicates non‐significance after FDR correction. (G and H) ESTIMATE‐based analysis of stromal score, immune score, ESTIMATE score and tumour purity in LUAD (G) and LUSC (H). Violin plots compare score distributions between high‐risk and low‐risk groups.

In both the LUAD and LUSC cohorts, the high‐risk groups exhibited expression profiles distinct from those of the low‐risk groups (Figure 7A,B). In LUAD, genes associated with redox homeostasis, iron metabolism and cellular stress were significantly upregulated in the high‐risk group (e.g., NQO1, OSGIN1, HMOX1, FTH1 and FTL),^74^, ^75^ whereas genes related to neuronal differentiation or relatively benign metabolic states were markedly downregulated (e.g., NEUROD1, NNAT and MYBPC1).^76^ Overall, these molecular features point toward enhanced antioxidant capacity and ferroptosis regulation alongside weakened differentiation programmes. In contrast, high‐risk LUSC tumours were primarily characterised by the upregulation of genes associated with basal‐like epithelial remodelling, barrier function and inflammatory responses (e.g., MATN4, CRABP1, ECEL1 and FOXC2),^77^ accompanied by the downregulation of certain receptors, transporters and metabolic enzymes, suggesting a more prominent epithelial remodelling and inflammation‐driven phenotype.

#### 3.6.2 Gene ontology enrichment analysis

Gene ontology (GO) enrichment analysis further demonstrated (Figure 7C,D) that the upregulated genes in the high‐risk LUAD group were enriched in metabolism‐related biological processes (BPs) such as the NADP metabolic process, ferroptosis and its regulation, and the pentose phosphate pathway. They were also enriched in cellular components (CCs) including chromatin structure and lysosomes/autophagosomes, as well as molecular functions (MFs) such as oxidoreductase activity, iron ion/bile acid binding and G‐protein regulation. These features indicate that high‐risk LUAD achieves a dynamic balance against oxidative damage and ferroptosis by enhancing NADPH supply, regulating iron homeostasis, and mediating reactive oxygen species responses, thereby promoting tolerance to chemotherapy/radiotherapy and driving tumour progression. In contrast, the upregulated genes in high‐risk LUSC were enriched in BPs related to hormone metabolism, nutritional response regulation, cholinergic synaptic transmission and various neural/ion channel‐related processes. Regarding CCs, enrichment was observed in the synaptic membrane, ion channel complexes and neurotransmitter receptor complexes; at the MF level, enrichment was skewed toward glutamate receptor binding, ion channel activity and endopeptidase inhibitor activity. This characteristic of neuro‐epithelial‐like signalling accompanied by barrier remodelling is consistent with recent reports on the involvement of neuromodulatory signals in LUSC invasion and immune evasion,^78^, ^79^, ^80^, ^81^, ^82^ implying that high‐risk LUSC resides within a highly active signal transduction and local inflammatory microenvironment, potentially leading to a poorer prognosis.

#### 3.6.3 Comparison of immune cell infiltration profiles

To systematically evaluate TIME differences between model‐derived high‐risk and low‐risk groups, we used TIMER, CIBERSORT, QUANTISEQ, MCP‐counter, xCell and EPIC to calculate immune/stromal cell infiltration scores and performed Mann–Whitney *U* tests and Benjamini–Hochberg FDR correction within each tumour subtype (Figure 7E,F, Table 1, Table S4). For each tumour subtype, 97 cell‐type × algorithm combinations were tested. After FDR correction, 48 combinations reached FDRq<.05 in LUAD, with 23 higher and 25 lower in the high‐risk group. In LUSC, 51 combinations reached FDRq<.05, with 13 higher and 38 lower in the high‐risk group.

**TABLE 1:** Representative immune and stromal cell infiltration score comparisons between model‐derived high‐risk and low‐risk groups in LUAD and LUSC.

Tumour subtype	Algorithm	Cell population/score	Direction in high‐risk group	Mean high risk	Mean low risk	Difference	SMD	Raw *p*‐value	FDR *q*‐value
LUAD	MCP‐counter	Endothelial cell	Lower	11.652	21.427	−9.775	−.685	4.00 × 10^−5^	4.27 × 10^−4^
LUAD	xCell	Endothelial cell	Lower	.0405	.1011	−.0606	−.709	1.01 × 10^−4^	8.11 × 10^−4^
LUAD	EPIC	Endothelial cell	Lower	.0394	.0785	−.0391	−.648	9.98 × 10^−5^	8.11 × 10^−4^
LUAD	xCell	stroma score	Lower	.0551	.1104	−.0553	−.661	2.30 × 10^−5^	3.15 × 10^−4^
LUAD	TIMER	T‐cell CD8^+^	Lower	.1431	.2116	−.0684	−.439	2.44 × 10^−4^	1.67 × 10^−3^
LUAD	CIBERSORT	NK cell resting	Lower	.0026	.0125	−.0099	−.549	9.85 × 10^−7^	4.73 × 10^−5^
LUAD	xCell	Macrophage M2	Lower	.0241	.0485	−.0243	−.630	2.03 × 10^−7^	1.95 × 10^−5^
LUAD	xCell	Neutrophil	Lower	.0012	.0071	−.0060	−.559	6.84 × 10^−6^	1.64 × 10^−4^
LUAD	xCell	B‐cell plasma	Higher	.0292	.0238	.0053	.202	2.04 × 10^−5^	3.15 × 10^−4^
LUAD	CIBERSORT	B‐cell plasma	Higher	.0772	.0649	.0123	.167	3.70 × 10^−4^	2.37 × 10^−3^
LUAD	CIBERSORT	T‐cell regulatory (Tregs)	Higher	.0222	.0170	.0052	.240	4.12 × 10^−4^	2.47 × 10^−3^
LUAD	EPIC	Cancer‐associated fibroblast	Higher	.0871	.0561	.0310	.339	3.74 × 10^−6^	1.20 × 10^−4^
LUSC	MCP‐counter	Endothelial cell	Lower	7.405	18.459	−11.054	−.734	2.31 × 10^−11^	1.68 × 10^−9^
LUSC	xCell	Endothelial cell	Lower	.0175	.0863	−.0688	−.781	1.35 × 10^−10^	4.38 × 10^−9^
LUSC	EPIC	Endothelial cell	Lower	.0232	.0684	−.0452	−.676	3.17 × 10^−9^	3.31 × 10^−8^
LUSC	xCell	Stroma score	Lower	.0235	.0867	−.0633	−.747	1.06 × 10^−9^	1.46 × 10^−8^
LUSC	xCell	Immune score	Lower	.1714	.2303	−.0589	−.343	1.69 × 10^−6^	6.01 × 10^−6^
LUSC	xCell	Monocyte	Lower	.0337	.0897	−.0560	−.740	3.47 × 10^−11^	1.68 × 10^−9^
LUSC	TIMER	Macrophage	Lower	.0368	.1148	−.0780	−.719	3.84 × 10^−9^	3.39 × 10^−8^
LUSC	EPIC	Macrophage	Lower	.0144	.0242	−.0098	−.588	9.34 × 10^−10^	1.46 × 10^−8^
LUSC	MCP‐counter	Neutrophil	Lower	1.004	14.383	−4.379	−.644	1.86 × 10^−10^	4.51 × 10^−9^
LUSC	TIMER	T‐cell CD8^+^	Lower	.1337	.2275	−.0939	−.581	1.72 × 10^−7^	8.78 × 10^−7^
LUSC	MCP‐counter	NK cell	Lower	.3155	.4965	−.1810	−.405	1.82 × 10^−5^	5.52 × 10^−5^
LUSC	QUANTISEQ	Uncharacterised cell	Higher	.7843	.6860	.0983	.653	5.20 × 10^−9^	4.20 × 10^−8^
LUSC	EPIC	Uncharacterised cell	Higher	.7693	.7142	.0551	.417	5.47 × 10^−6^	1.77 × 10^−5^
LUSC	xCell	T‐cell CD4^+^ Th1	Higher	.0867	.0607	.0260	.491	4.72 × 10^−8^	2.55 × 10^−7^
LUSC	xCell	B‐cell plasma	Higher	.0318	.0244	.0074	.276	2.59 × 10^−4^	7.38 × 10^−4^

*Note*: Mean high‐risk, mean low‐risk and difference were calculated from the original immune‐infiltration scores rather than row‐wise *Z*‐score‐transformed heatmap values. Difference was defined as mean high risk minus mean low risk. Between‐group comparisons were performed using Mann–Whitney *U* tests, followed by Benjamini–Hochberg FDR correction across all tested cell‐type × algorithm combinations within each tumour subtype. Only representative rows are shown; the full statistical results are provided in Table S4.

Abbreviations: CAF, cancer‐associated fibroblast; SMD, standardised mean difference; Tregs, regulatory T cells.

In LUAD, endothelial cell scores in the high‐risk group were lower than those in the low‐risk group across multiple algorithms, including MCP‐counter ($\mathrm{FDR}\;q\;=4.27\times 10^{-4}$), xCell ($\mathrm{FDR}\;q\;=8.11\times 10^{-4}$) and EPIC ($\mathrm{FDR}\;q\;=8.11\times 10^{-4}$). The xCell stroma score was also lower in the high‐risk group ($\mathrm{FDR}\;q\;=3.15\times 10^{-4}$). For immune cell estimates, xCell‐estimated Macrophage M2 ($\mathrm{FDR}\;q\;=1.95\times 10^{-5}$), CIBERSORT‐estimated resting NK cells ($\mathrm{FDR}\;q\;=4.73\times 10^{-5}$), xCell‐estimated neutrophils ($\mathrm{FDR}\;q\;=1.64\times 10^{-4}$) and TIMER‐estimated CD8^+^ T cells ($\mathrm{FDR}\;q\;=1.67\times 10^{-3}$) were lower in the high‐risk group. In contrast, selected B‐lineage/plasma‐cell estimates were higher in the high‐risk group, such as xCell B cell plasma ($\mathrm{FDR}\;q\;=3.15\times 10^{-4}$) and CIBERSORT B cell plasma ($\mathrm{FDR}\;q\;=2.37\times 10^{-3}$). The EPIC‐estimated CAF score was also higher in the high‐risk group ($\mathrm{FDR}\;q\;=1.20\times 10^{-4}$). It should be noted that CAFs, Tregs, NK cells and macrophage‐related estimates showed directional differences across algorithms; therefore, these cell populations should not be generalised as consistently increased or decreased across algorithms.

In LUSC, the high‐risk group was mainly characterised by lower stromal/endothelial, myeloid/macrophage, neutrophil and selected cytotoxic lymphocyte‐related estimates. Endothelial cell scores were lower than those in the low‐risk group in MCP‐counter ($\mathrm{FDR}\;q\;=1.68\times 10^{-9}$), xCell ($\mathrm{FDR}\;q\;=4.38\times 10^{-9}$) and EPIC ($\mathrm{FDR}\;q\;=3.31\times 10^{-8}$). The xCell stroma score ($\mathrm{FDR}\;q\;=1.46\times 10^{-8}$) and xCell immune score ($\mathrm{FDR}\;q\;=6.01\times 10^{-6}$) were also lower in the high‐risk group. Myeloid‐related estimates, including xCell monocytes ($\mathrm{FDR}\;q\;=1.68\times 10^{-9}$), TIMER macrophages ($\mathrm{FDR}\;q\;=3.39\times 10^{-8}$) and EPIC macrophages ($\mathrm{FDR}\;q\;=1.46\times 10^{-8}$), all showed lower values in the high‐risk group. Neutrophil estimates were lower in MCP‐counter ($\mathrm{FDR}\;q\;=4.51\times 10^{-9}$). TIMER‐estimated CD8^+^ T cells ($\mathrm{FDR}\;q\;=8.78\times 10^{-7}$) and MCP‐counter‐estimated NK cells ($\mathrm{FDR}\;q\;=5.52\times 10^{-5}$) were also reduced in the high‐risk group. Conversely, the QUANTISEQ uncharacterised cell fraction ($\mathrm{FDR}\;q\;=4.20\times 10^{-8}$), xCell T cell CD4^+^ Th1 ($\mathrm{FDR}\;q\;=2.55\times 10^{-7}$) and xCell B cell plasma ($\mathrm{FDR}\;q\;=7.38\times 10^{-4}$) were higher in the high‐risk group.

Overall, Figure 7E,F and Table S4 show that SparseAGE‐MTL‐derived risk status was associated with subtype‐specific and algorithm‐dependent immune/stromal infiltration differences. Because some immune cell categories showed non‐identical directions across deconvolution algorithms, we interpret these results as bulk transcriptomics‐derived TIME profiling rather than reducing them to a single pattern in which all suppressive cells are consistently increased, or all cytotoxic cells are consistently decreased.

#### 3.6.4 Distribution of cold and hot tumour phenotypes

Furthermore, based on the ESTIMATE scoring system (Table S3), we compared stromal scores, immune scores, total ESTIMATE scores and tumour purity across different risk groups (Figure 7G,H). In LUAD, the stromal and immune scores in the high‐risk group were significantly lower than those in the low‐risk group (*p* < .01 or *p* < .001), and the total ESTIMATE score was also markedly decreased, whereas tumour purity was significantly elevated. These results indicate that high‐risk LUAD more closely resembles a classical ‘cold tumour’: it is relatively deficient in immune cells and stromal components, possesses a higher proportion of tumour cells, and likely presents a lower potential response rate to immunotherapy. Similar but more pronounced trends were observed in LUSC, where the stromal, immune and ESTIMATE scores of the high‐risk group were generally in the lower range, while tumour purity was in the higher range (all *p* < .0001). In contrast, low‐risk LUSC typically exhibited higher immune and stromal infiltration and lower tumour purity, aligning more closely with the characteristics of a ‘hot tumour’. This finding is mutually corroborated by the aforementioned spatial transcriptomic data indicating that the high‐risk LUSC niche lacks effective antitumour immunity, but is enriched with hypoxia and stress response signals.

### 3.7 Clinical incremental value of the SparseAGE‐MTL risk score

**FIGURE 8:**
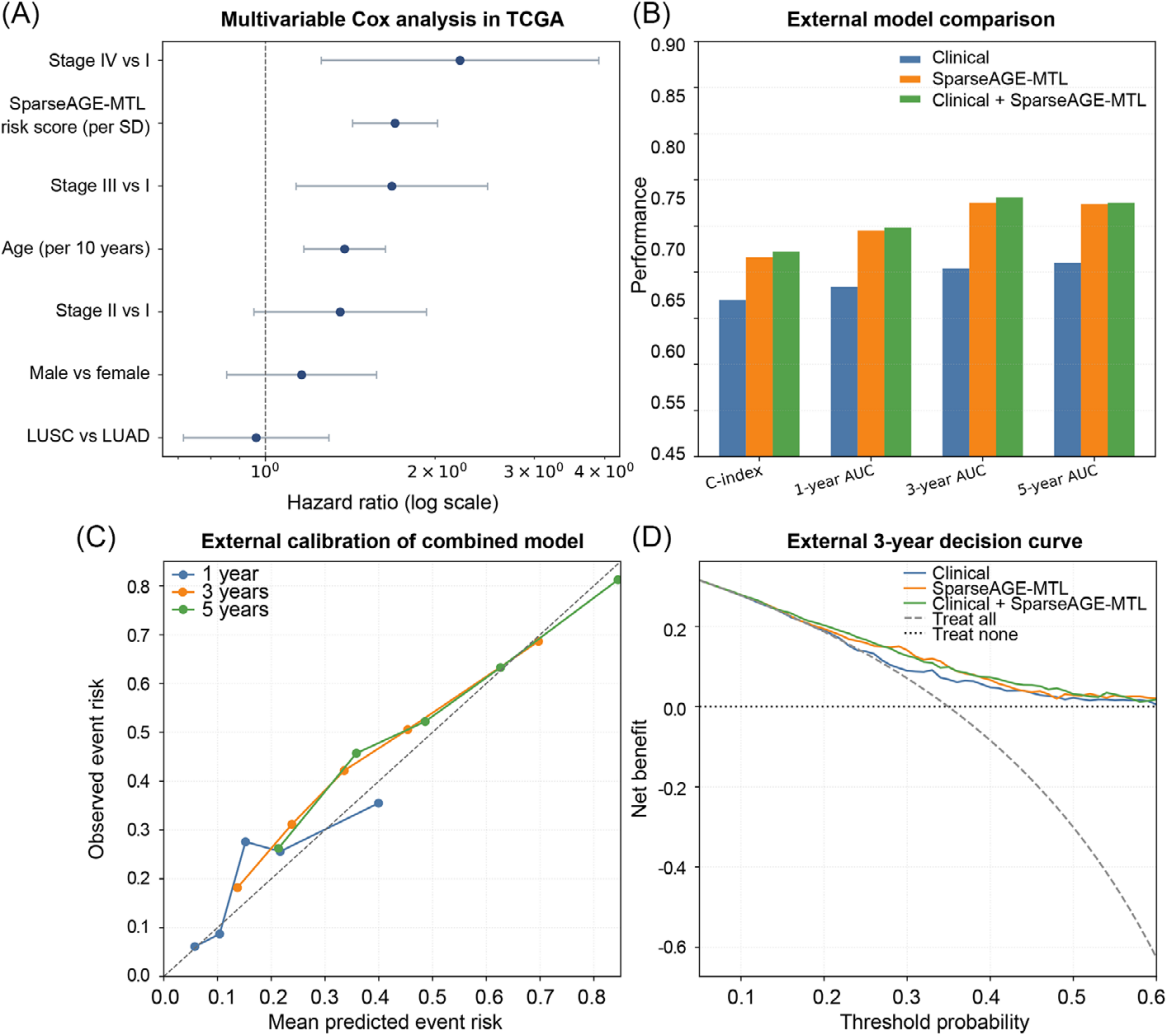
Clinical incremental value of the SparseAGE‐MTL risk score. (A) Multivariable Cox proportional hazards analysis in the TCGA cohort. The model includes the standardised SparseAGE‐MTL risk score and clinical variables, including age, sex, pathological subtype and pathological stage. Points indicate hazard ratios, and horizontal bars indicate 95% confidence intervals. (B) External pooled validation of prognostic model performance. The clinical model, SparseAGE‐MTL risk score model and combined model are compared using *C*‐index and time‐dependent AUCs at 1, 3 and 5 years. (C) Calibration curves of the combined model for 1‐, 3‐ and 5‐year survival probability prediction in the external pooled validation cohort. (D) Three‐year decision curve analysis comparing the clinical model, SparseAGE‐MTL risk score model and combined model. The *x*‐axis indicates threshold probability, and the *y*‐axis indicates net benefit.

To evaluate whether the WSI‐derived risk score provides prognostic information beyond clinical variables, we further constructed a clinical model, a SparseAGE‐MTL risk score model and a combined model. Multivariable Cox analysis showed that after including age, sex, pathological subtype and pathological stage, the standardised SparseAGE‐MTL risk score remained independently associated with OS (per 1 SD increase, HR = 1.70, 95% CI: 1.43–2.02) (Figure 8A). In external pooled validation cohorts, the *C*‐index and time‐dependent AUCs of the combined model were slightly higher than those of the clinical model; the *C*‐indices of the clinical model, SparseAGE‐MTL model and combined model were approximately .620, .666 and .672, respectively (Figure 8B). Calibration curves showed that the combined model had an acceptable calibration pattern for 1‐, 3‐ and 5‐year survival probability prediction, although deviations remained at different time points (Figure 8C). Decision curve analysis showed that the combined model had relatively higher net benefit over selected threshold probability ranges, suggesting that the SparseAGE‐MTL risk score may provide modest incremental value for clinical prognostic modelling (Figure 8D).

## 4 DISCUSSION

Computational pathology has demonstrated promising results in solid tumour research and clinical evaluation; however, limited generalisability across different centres, the predominant focus of existing methods on single tasks, and lack of sufficient biological interpretability remain major challenges for the clinical translation of current digital pathology analyses. This study proposes a multi‐task SparseAGE‐MTL framework that operates under weakly supervised learning conditions without requiring region of interest (ROI) or cell‐level annotations, enabling subtype classification, stage inference and survival risk prediction for NSCLC based on routine H&E whole‐slide images (WSIs). This framework provides a research basis for morphology‐based multi‐task evaluation and spatially grounded biological interpretation in non‐small cell lung cancer.

SparseAGE‐MTL uses a shared projection‐topology encoder combined with task‐specific attention pooling, residual task adapters, endpoint‐specific output heads, and a lightweight topology auxiliary head to achieve joint multi‐task learning for subtype classification, stage prediction and survival risk estimation. Across seven pretrained feature spaces and 77 feature‐endpoint‐metric settings, SparseAGE‐MTL achieved the lowest overall mean rank and, together with the non‐MTL comparator SparseAGE‐MIL, formed the top‐ranked SparseAGE family. Its subtype classification performance was nearly saturated in the source cohort and maintained high AUCs on FFPE WSIs and TMAs from two external hospitals, suggesting that topology‐aware sparse graph embedding may help mitigate domain shifts caused by differences in slide preparation, scanning protocols and patient populations. This cross‐cohort generalisation ability may contribute to addressing external validation gaps in digital pathology studies.^83^ Meanwhile, SparseAGE‐MTL unifies subtyping, staging and survival prediction within a shared feature space and topology aggregation mechanism, which may reduce parameter redundancy and improve the efficiency of limited‐sample use. It should be noted that the benchmark primarily supports the cross‐feature and cross‐endpoint ranking stability of SparseAGE‐MTL rather than proving large improvements in every individual setting. The topology diagnostics in Figure 4 show that the Spatial‐Feature Topology Aggregator can alter the patch‐level embedding distribution and establish Top‐*K* topology not restricted to immediate grid adjacency, providing mechanistic visualisation and quantitative support for topology‐aware MIL design.

By co‐registering with Visium spatial transcriptomics data, this study observed that high‐attention regions of SparseAGE‐MTL in LUSC are not simply equivalent to regions with the highest tumour cell density. Instead, they are more concentrated at the tumour–stroma interface and highly overlap with band‐like enrichment of B cells and fibroblasts, high expression of C1QC/COL1A1, and functional modules such as EMT, metastasis and hypoxia. This spatial pattern is highly consistent with the previously reported invasive front and tumour–immune–stromal tripartite junction, which are considered critical niches driving tumour progression and immune evasion.^84^ Furthermore, within high‐attention LUSC cells, stress responses, HIF‐1/MAPK/AMPK signalling, protein processing and glycolysis pathways were significantly upregulated, whereas genes associated with adhesion and maintenance of metabolic homeostasis were suppressed. This combination of high‐stress and high‐adaptability pathways is typically associated with higher risk of metastasis, therapeutic resistance and poor prognosis.^85^ Therefore, SparseAGE‐MTL attention hotspots point to a highly invasive and highly adaptable high‐risk LUSC niche at both spatial and molecular levels, providing a specific biological explanation for the model's focal areas. Spatially, if hypoxia/HIF‐1 activity is concentrated at the tumour–stroma/immune interface or in tumour‐dense hypoxic spots, it may locally form a hypoxia‐metabolism‐immune coupled microenvironment. Existing studies suggest that HIF‐1α‐related programmes may participate in restricted antitumour immunity through glycolysis, acidosis, abnormal vascular remodelling, adenosine‐related immunosuppression and immune‐evasion signals such as PD‐L1/CD47, and may affect the efficacy of immune checkpoint blockade.^86^, ^87^ Under this spatial architecture, cytotoxic lymphocytes may have difficulty entering tumour cell‐enriched hypoxic niches or maintaining effector function even if they are present in adjacent regions. This mechanistic hypothesis is consistent with the tumour–stroma/immune interface attention enrichment, HIF‐1 pathway enrichment, hypoxia‐related GSEA signals and decreased selected immune/stromal estimates in high‐risk groups observed in this study (Figures 6B,G,H and 7E–H, Figure S8A–D).

At the bulk transcriptomic level, high‐risk subgroups identified by SparseAGE‐MTL showed activation of pathways typically associated with malignant progression in both LUAD and LUSC. In the high‐risk LUAD group, NQO1, OSGIN1, HMOX1 and iron metabolism‐related genes were significantly upregulated and enriched in pathways such as NADP metabolism, ferroptosis regulation and the pentose phosphate pathway, suggesting that tumours may enhance DNA damage tolerance and therapeutic resistance by remodelling redox and iron homeostasis.^88^ Conversely, the high‐risk LUSC group showed significant enrichment in pathways related to hormone metabolism, cholinergic/glutamatergic synaptic transmission, and ion channels, along with upregulation of genes associated with epithelial remodelling and inflammation‐driven processes, such as MATN4, CRABP1 and FOXC2, suggesting that neuro‐epithelial interactions and local inflammation play critical roles in high‐risk LUSC.^78^ Multi‐algorithm immune infiltration analysis showed that SparseAGE‐MTL‐derived high‐risk status was associated with subtype‐specific and algorithm‐dependent TIME differences (Figure 7E,F, Table 1, Table S4). In LUAD, endothelial cell estimates, xCell stroma score, TIMER‐estimated CD8^+^ T cells, CIBERSORT‐estimated resting NK cells, xCell‐estimated neutrophils and xCell‐estimated macrophage M2 were all lower than those in the low‐risk group after FDR correction; in contrast, selected B‐lineage/plasma‐cell estimates and EPIC‐estimated CAF score were higher in the high‐risk group. In LUSC, the high‐risk group showed broader decreases in endothelial, stromal, monocyte/macrophage, neutrophil, CD8^+^ T‐cell and NK‐cell estimates across multiple algorithms, while some algorithms showed higher uncharacterised‐cell fraction, Th1‐related estimates and plasma‐cell estimates in the high‐risk group. These results suggest that model‐derived high‐risk status is associated with altered TIME, mainly characterised by lower stromal/endothelial signals and selected immune‐cell infiltration signals. However, these results should not be interpreted as consistent increases in all suppressive cell populations in the high‐risk group. Because these estimates are derived from bulk transcriptomics‐based deconvolution, cell‐type‐specific directions require further validation using spatial transcriptomics, single‐cell RNA‐seq, multiplex immunofluorescence, or related approaches before being used for treatment selection.^12^, ^89^ ESTIMATE results further support differences in immune/stromal scores and tumour purity between model‐derived risk groups. Previous studies suggest that cold‐like or immune‐excluded tumour microenvironments may be associated with limited immune checkpoint blockade response.^90^, ^91^ However, this study did not include immunotherapy‐treated cohorts and did not directly evaluate treatment response; therefore, the immunotherapy‐related clinical implications of the SparseAGE‐MTL risk score require further validation in independent treatment‐response cohorts.^92^

In addition, clinical utility analysis showed that the SparseAGE‐MTL risk score retained prognostic information after adjustment for age, sex, pathological subtype and pathological stage, and it provided small improvements in *C*‐index and time‐dependent AUC in the external pooled cohorts compared with the clinical model. Decision curve analysis also suggested that the combined model had relatively higher net benefit over selected threshold probability ranges. However, this result should be interpreted cautiously: the magnitude of incremental benefit was limited and still based on retrospective cohorts and pooled external validation. Future prospective cohorts and treatment‐specific cohorts are needed to evaluate whether WSI‐derived risk scores add reproducible prognostic information beyond clinical variables.

Despite these findings, this study has several limitations. First, although multicentre cohorts and two slide formats (WSI, TMA) were included, external validation was mainly based on surgically resected Chinese cases; generalisability to advanced‐stage disease, systemic therapy‐treated cohorts, biopsies and non‐Chinese populations remains unverified. Future work should adopt a pre‐registered, prospective, multinational validation design with locked model, pipeline and endpoints. Centres should span diverse regions with prespecified stratification across key clinical and technical factors. Cohorts should include resection, advanced disease, and patients receiving standard systemic therapies. Primary endpoints include subtype/stage discrimination, survival stratification, calibration and *C*‐index/AUC, while immunotherapy outcomes are exploratory. Analysis should use locked‐model external validation with assessment of domain shift and recalibration. Reporting should follow TRIPOD+AI and PROBAST+AI^93^, ^94^; clinical translation should follow SPIRIT‐AI and CONSORT‐AI.^95^, ^96^ Second, the spatial transcriptomic validation currently relies on a limited number of samples, which is insufficient to systematically cover the full morphological and molecular heterogeneity of NSCLC, and technical discrepancies between platforms may also introduce bias into cell typing and functional annotations. This issue can be mitigated in future studies by incorporating large‐scale, multicentre data combined with cross‐platform alignment methods. Furthermore, although this study identified associations between high‐attention niches and high‐risk transcriptomic profiles in LUSC, the underlying mechanisms require further elucidation. Future studies should employ corresponding functional experiments and animal models to explore the causal mechanisms of hypoxia–metabolism–immune interactions in tumour progression and immune resistance. Additionally, the calibration analyses showed that predicted probabilities were not uniformly calibrated across cohorts and tasks. Subtype classification retained relatively high discrimination in external cohorts, but external calibration was moderate. Stage prediction calibration was affected by class imbalance and limited external stage‐specific sample sizes, and survival calibration showed timepoint‐dependent deviations in external validation. Therefore, SparseAGE‐MTL outputs should currently be interpreted as morphology‐derived risk or probability estimates rather than fully calibrated clinical probabilities. Prospective validation and, if needed, cohort‐specific recalibration will be required before clinical deployment. Future work may evaluate whether integration with radiomics, circulating tumour DNA, or immune biomarkers improves risk assessment in locked, prospective and treatment‐annotated cohorts.

## 5 CONCLUSIONS

This study developed SparseAGE‐MTL, a weakly supervised joint multi‐task MIL framework for NSCLC H&E WSIs that can simultaneously perform subtype classification, pathological stage prediction and survival risk estimation. Benchmark comparisons showed stable competitive performance across feature spaces and endpoints, with partial generalisation to external WSI and TMA cohorts. Topology diagnostics, spatial transcriptomics co‐registration and bulk transcriptomics/TIME profiling jointly suggested that model attention and WSI‐derived risk stratification were associated with the tumour–stroma/immune interface, malignant pathway activation and subtype‐specific immune/stromal infiltration differences. Clinical utility analysis further suggested that the SparseAGE‐MTL risk score may provide modest incremental information for clinical prognostic models. Overall, this framework provides a methodological basis for constructing pathology AI models with spatial and biological interpretability, but clinical deployment still requires further evaluation in prospective validation and treatment‐response cohorts.

## AUTHOR CONTRIBUTIONS

*Conceptualisation*: P.L., A.Q.L., Q.L.L., S.Y.F. and Q.C. *Formal analysis*: R.Y.L., J.Y.S., A.M.J. and Q.C. *Resources*: A.Q.L. and P.L. *Software*: R.Y.L., J.Y.S., A.M.J. and Q.C. *Supervision*: P.L., A.Q.L., Q.L.L., S.Y.F. and Q.C. *Visualisation*: R.Y.L., J.Y.S., A.M.J. and Y.Y.F. *Writing—original draft*: R.Y.L., J.Y.S., A.M.J., Y.Y.F., X.H.F., Y.F.B., S.K.P., J.Z., Q.C., S.Y.F., Q.L.L. and P.L. *Writing—review and editing*: P.L., A.Q.L. and Q.C. All authors have read and approved the final manuscript.

## CONFLICT OF INTEREST STATEMENT

The authors declare that the research was conducted in the absence of any commercial or financial relationships that could be construed as a potential conflict of interest.

## ETHICS STATEMENT

This multicenter retrospective study received approval from the Ethics Committee of the Sichuan Academy of Medical Sciences & Sichuan Provincial People's Hospital (Approval No.: KY‐2022‐11) and the Ethics Committee of Zhujiang Hospital, Southern Medical University (Approval No.: 2018‐ZLK‐003). The use of public datasets adhered to the TCGA data access and sharing policies, in accordance with all relevant ethical guidelines.

## Supporting information



Supporting Information

Supporting Information

Supporting Information

Supporting Information

Supporting Information

Supporting Information

Supporting Information

Supporting Information

Supporting Information

Supporting Information

Supporting Information

Supporting Information

Supporting Information

Supporting Information

Supporting Information

Supporting Information

Supporting Information

Supporting Information

## Data Availability

The public TCGA‐LUAD and TCGA‐LUSC datasets used in this study, including H&E‐stained whole‐slide images, clinicopathological annotations, survival information and bulk transcriptomic data, are available through the Genomic Data Commons Data Portal (https://portal.gdc.cancer.gov/) in accordance with TCGA data access and sharing policies. The public 10x Genomics Visium spatial transcriptomics datasets used for spatial transcriptomic interpretation are available from the 10x Genomics public dataset portal. The external SMUZH‐NSCLC and SAMSPH‐NSCLC cohorts were collected under institutional ethical approval and are not publicly available because of patient privacy protection and institutional restrictions on the sharing of clinical and digital pathology data. Access to de‐identified institutional data may be considered by the corresponding author upon reasonable request and subject to approval by the relevant ethics committees, institutional data‐sharing policies and applicable regulations. The source code for SparseAGE‐MTL, including model implementation, training pipelines and evaluation scripts, is publicly available at https://github.com/Kinajus/SparseAGE‐MTL. Detailed documentation, installation instructions and example scripts for reproducing the main experiments are provided in the repository.
