# Supplementary material for: An interpretable multi‐task whole‐slide histopathology AI model for non‐small cell lung cancer: Cross‐cohort generalisation, spatial attention–transcriptomic integration, and molecular–immune profiling
Source: Clin Transl Med. 2026 Jul 23;16(7):e70744. doi: 10.1002/ctm2.70744 (PMC13396892; doi:10.1002/ctm2.70744)
Supplement: Supplementary file 1 — Supporting Information [file CTM2-16-e70744-s010.docx]

**Supporting Information**

**An Interpretable Multi‑Task Whole‑Slide Histopathology AI Model for Non‑Small Cell Lung Cancer: Cross‑Cohort Generalization, Spatial Attention–Transcriptomic Integration, and Molecular–Immune Profiling**

Supplementary Methods

**1 SparseAGE-MTL: Sparse Adaptive Graph Encoding with Spatial-Topology Regularization for Joint Multi-Task Multiple Instance Learning**

**1.1 Multiple instance learning**

In the Multiple Instance Learning (MIL) framework, each Whole Slide Image (WSI) is treated as a bag $\text{X}\text{=\{}\text{x}_{\text{1}}\text{,…,}\text{x}_{\text{I}}\text{\}}$ composed of local image patches, where $\text{x}_{\text{i}}$ represents the $\text{i}$-th instance and $\text{I}$ denotes the total number of patches, which is determined by the slide dimensions and the cropping strategy. Given only the bag-level label $\text{Y}$, the individual instance labels $\text{y}_{\text{i}}$ remain latent; thus, the mapping from the instance set to the global prediction must be performed under weakly supervised conditions:

$$\begin{matrix} Y=\left\{ \begin{matrix} 1, & if \exists y_{i}=1, i\in\{1,2,\ldots,I\}, \\ 0, & otherwise. \end{matrix} \right. \end{matrix}$$

The classical three-stage MIL framework can be formulated as follows [1]:

$$\text{H}\text{=}\text{F}\text{(}\text{X}\text{)}$$

$$\text{M}\text{=}\text{A}\text{(}\text{H}\text{)}$$

$$\hat{\text{Y}}\text{=}\text{C}\text{(}\text{M}\text{)}$$

where $\text{F}$ is the instance-level feature extractor, $\text{A}$ is the instance feature aggregator, and $\text{C}$ is the bag-level classifier. The feature representations at each stage are as follows: $\text{H}\text{=\{}\text{h}_{\text{1}}\text{,…,}\text{h}_{\text{I}}\text{\}∈}\text{R}^{\text{I}\text{×}\text{d}}$ represents the instance feature matrix; $\text{M}$ represents the bag-level feature vector; and $\hat{\text{Y}}$ is the predicted value for $\text{Y}$.

In this study, we employ SparseAGE-MTL, namely Sparse Adaptive Graph Encoding with Spatial-Topology Regularization for Joint Multi-Task Multiple Instance Learning. SparseAGE-MTL extends the classical MIL workflow into a shared projection-topology encoder followed by task-specific pooling, task adapters, endpoint-specific prediction heads, and a lightweight topology auxiliary branch:

$$H=\mathcal{\mathcal{F}}(X)$$

$$H'=\mathcal{P}(H)$$

$$Z=\mathcal{T}(H',R,L)$$

$$M_{m}=\mathcal{A}_{m}(Z)$$

$$\tilde{M}_{m}=M_{m}+{Adapter}_{m}(M_{m})$$

$$\hat{Y}_{m}=\mathcal{C}_{m}(\tilde{M}_{m})$$

$$\bar{M}=|\mathcal{\mathcal{M}}|^{-1}\sum_{m\in\mathcal{\mathcal{M}}} \tilde{M}_{m}$$

$$\hat{q}=\mathcal{Q}(\bar{M})$$

Here, $\text{F}$ denotes the offline instance-level feature extractor, $\text{P}$ denotes the shared Feature Projection Network, and $\text{T}$ denotes the Spatial-Feature Topology Aggregator. $\text{R}$ denotes optional patch coordinates, $\text{L}$ denotes optional pseudo-histology cluster labels, and $\text{Z}\text{=\{}\text{z}_{\text{i}}{\text{\}}}_{\text{i}\text{=1}}^{\text{I}}\text{∈}\text{R}^{\text{I}\text{×}\text{D}}$ denotes the topology-regularized patch embedding matrix. The task index $\text{m}$ belongs to the endpoint set $\text{M}\text{=\{}\text{subtype}\text{,}\text{stage}\text{,}\text{survival}\text{\}}$. Each endpoint has an independent attention pooling module $\text{A}_{\text{m}}$, Residual Task Adapter, and output head $\text{C}_{\text{m}}$, while $\text{P}$ and $\text{T}$ are shared across endpoints. $\text{Q}$ denotes the lightweight topology auxiliary head, and $\hat{\text{q}}$ denotes the predicted topology descriptor. SparseAGE-MIL denotes the corresponding non-MTL comparator and is used only as an internal control, a single-task setting, or an ablation model.

**1.2 Image patching and Instance-level Feature Extractor**

The instance-level feature extractor $\text{F}$ maps the set of image patches $\text{X}\text{=\{}\text{x}_{\text{1}}\text{,}\text{x}_{\text{2}}\text{,…,}\text{x}_{\text{I}}\text{\}}$ to an instance feature matrix:

$$\text{H}\text{=}\text{F}\text{(}\text{X}\text{)=\{}\text{h}_{\text{1}}\text{,}\text{h}_{\text{2}}\text{,…,}\text{h}_{\text{I}}\text{\}∈}\text{R}^{\text{I}\text{×}\text{d}}$$

where $\text{d}$ is the feature dimensionality, which depends on the specific feature extractor utilized.

**1.3 Feature Projection Network**

The Feature Projection Network maps offline patch embeddings generated by a pretrained feature extractor into a unified shared latent space, thereby improving the comparability of different feature spaces for subsequent topology aggregation and multi-task prediction. This study does not concatenate embeddings from multiple pretrained backbones at the patch level and does not fuse multiple backbones within a single forward pass. Each experiment loads only the feature space corresponding to one pretrained feature extractor and uses the same Feature Projection Network architecture to project embeddings from that feature space into a latent space with a consistent dimensionality. Differences among feature extractors are evaluated through matched benchmark comparison, feature-extractor sensitivity analysis, and default feature space selection.

For a patch embedding $\text{h}_{\text{i}}^{\text{(}\text{f}\text{)}}\text{∈}\text{R}^{\text{d}_{\text{f}}}$ from feature extractor $\text{f}$, projection consists of a linear transformation, ReLU activation, dropout, and LayerNorm:

$$\text{u}_{\text{i}}^{\text{(}\text{f}\text{)}}\text{=}\text{h}_{\text{i}}^{\text{(}\text{f}\text{)}}\text{W}_{\text{P}}^{\text{(}\text{f}\text{)}}\text{+}\text{b}_{\text{P}}^{\text{(}\text{f}\text{)}}$$

$$\text{v}_{\text{i}}^{\text{(}\text{f}\text{)}}\text{=}\text{ReLU}\text{(}\text{u}_{\text{i}}^{\text{(}\text{f}\text{)}}\text{)}$$

$$\text{h}_{\text{i}}\text{'=}\text{LayerNorm}\text{(}\text{Dropout}\text{(}\text{v}_{\text{i}}^{\text{(}\text{f}\text{)}}\text{,}\text{p}\text{))}$$

In the default setting, the projection dimension is $\text{D}\text{=512}$ and the dropout rate is $\text{p}\text{=0.25}$. The projected patch embedding matrix is:

$$\text{H}\text{'=\{}\text{h}_{\text{1}}\text{',}\text{h}_{\text{2}}\text{',…,}\text{h}_{\text{I}}\text{'\}∈}\text{R}^{\text{I}\text{×}\text{D}}$$

Here, $\text{d}_{\text{f}}$ denotes the original embedding dimension of feature extractor $\text{f}$, and $\text{D}$ denotes the unified latent dimension output by the Feature Projection Network. The subsequent Spatial-Feature Topology Aggregator, task-specific attention pooling, and endpoint-specific output heads all receive $\text{H}\text{'}$ rather than a concatenated embedding from multiple feature extractors.

**1.4 Spatial-Feature Topology Aggregator**

The Spatial-Feature Topology Aggregator constructs sparse patch neighborhoods by integrating feature affinity and a spatial-topology prior. Given the projected embedding matrix $\text{H}\text{'∈}\text{R}^{\text{I}\text{×}\text{D}}$, the module first computes query, key, and value projections:

$$\text{Q}\text{=}\text{H}\text{'}\text{W}_{\text{Q}}$$

$$\text{K}\text{=}\text{H}\text{'}\text{W}_{\text{K}}$$

$$\text{V}\text{=}\text{H}\text{'}\text{W}_{\text{V}}$$

The feature-affinity score between patch $\text{i}$ and patch $\text{j}$ is defined as scaled dot-product similarity:

$$\text{S}_{\text{ij}}^{\text{feat}}\text{=}\frac{\text{Q}_{\text{i}}\text{K}_{\text{j}}^{\text{T}}}{\sqrt{\text{D}}}$$

When patch coordinates are available, a spatial proximity prior is introduced:

$$\text{S}_{\text{ij}}^{\text{spatial}}\text{=-}\text{λ}_{\text{s}}\log\text{(}\text{1+}\text{d}_{\text{ij}}\text{/}\text{σ}_{\text{s}}\text{)}$$

Here, $\text{d}_{\text{ij}}$ denotes the Euclidean distance between two patch coordinates, $\text{σ}_{\text{s}}$ denotes the spatial scale parameter, and $\text{λ}_{\text{s}}$ denotes the spatial prior weight.

When pseudo-histology cluster labels are available, same-cluster coherence and cross-cluster interface priors are introduced:

$$\text{S}_{\text{ij}}^{\text{cluster}}\text{=}\text{λ}_{\text{c}}\text{1}\text{(}\text{l}_{\text{i}}\text{=}\text{l}_{\text{j}}\text{)}$$

$$\text{S}_{\text{ij}}^{\text{interface}}\text{=}\text{λ}_{\text{b}}\text{1}\text{(}\text{l}_{\text{i}}\text{≠}\text{l}_{\text{j}}\text{)}\text{exp}\text{(-}\text{d}_{\text{ij}}\text{/}\text{σ}_{\text{s}}\text{)}$$

The final score used for neighbor selection is:

$$\text{S}_{\text{ij}}\text{=}\text{S}_{\text{ij}}^{\text{feat}}\text{+}\text{S}_{\text{ij}}^{\text{spatial}}\text{+}\text{S}_{\text{ij}}^{\text{cluster}}\text{+}\text{S}_{\text{ij}}^{\text{interface}}$$

When a certain type of metadata is unavailable, the corresponding term is set to 0. In the default joint multi-task setting, $\text{λ}_{\text{s}}\text{=0.20}$, $\text{λ}_{\text{c}}\text{=0.15}$, $\text{λ}_{\text{b}}\text{=0.10}$, and $\text{σ}_{\text{s}}\text{=256.0}$. Self-neighborhood is retained by default, meaning that include_self is true.

For multi-scale sparse aggregation, the model uses the Top-K set $\text{K}\text{=\{4,8,16\}}$. For each $\text{k}\text{∈}\text{K}$, the sparse neighborhood of patch $\text{i}$ is defined as:

$$\text{N}_{\text{k}}\text{(}\text{i}\text{)=}\text{TopK}_{\text{j}}\text{(}\text{S}_{\text{ij}}\text{,}\text{k}\text{)}$$

The first-stage affinity weights are defined as:

$$\text{α}_{\text{ij}}^{\text{(}\text{k}\text{)}}\text{=}\text{softmax}_{\text{j}\text{∈}\text{N}_{\text{k}}\text{(}\text{i}\text{)}}\text{(}\text{S}_{\text{ij}}\text{)}$$

The model then fuses the source query and the neighbor value through a message gate:

$$\text{G}_{\text{ij}}^{\text{(}\text{k}\text{)}}\text{=}\text{σ}\text{(}\text{W}_{\text{G}}\text{[}\text{Q}_{\text{i}}\text{,}\text{V}_{\text{j}}\text{]+}\text{b}_{\text{G}}\text{)}$$

$${\tilde{\text{V}}}_{\text{ij}}^{\text{(}\text{k}\text{)}}\text{=}\text{G}_{\text{ij}}^{\text{(}\text{k}\text{)}}\text{⊙}\text{V}_{\text{j}}\text{+(1-}\text{G}_{\text{ij}}^{\text{(}\text{k}\text{)}}\text{)⊙}\text{Q}_{\text{i}}$$

The second-stage gated compatibility score is defined as:

$$\text{E}_{\text{ij}}^{\text{(}\text{k}\text{)}}\text{=}\frac{{\tilde{\text{V}}}_{\text{ij}}^{\text{(}\text{k}\text{)}}\text{Q}_{\text{i}}^{\text{T}}}{\sqrt{\text{D}}}$$

The final neighbor weights integrate the gated compatibility score with the first-stage affinity weights:

$$\text{β}_{\text{ij}}^{\text{(}\text{k}\text{)}}\text{=}\text{softmax}_{\text{j}\text{∈}\text{N}_{\text{k}}\text{(}\text{i}\text{)}}\text{(}\text{E}_{\text{ij}}^{\text{(}\text{k}\text{)}}\text{+}\log\text{(}\text{α}_{\text{ij}}^{\text{(}\text{k}\text{)}}\text{+ϵ))}$$

The message at scale $\text{k}$ is:

$$\text{m}_{\text{i}}^{\text{(}\text{k}\text{)}}\text{=}\sum_{\text{j}\text{∈}\text{N}_{\text{k}}\text{(}\text{i}\text{)}} \text{β}_{\text{ij}}^{\text{(}\text{k}\text{)}}{\tilde{\text{V}}}_{\text{ij}}^{\text{(}\text{k}\text{)}}$$

Messages from multiple Top-K scales are fused through learnable scale weights:

$$\text{ρ}_{\text{i}}\text{=}\text{softmax}\text{(}\text{W}_{\text{ρ}}\text{Q}_{\text{i}}\text{+}\text{b}_{\text{ρ}}\text{)}$$

$$\text{m}_{\text{i}}\text{=}\sum_{\text{k}\text{∈}\text{K}} \text{ρ}_{\text{i}\text{,}\text{k}}\text{m}_{\text{i}}^{\text{(}\text{k}\text{)}}$$

The final topology-regularized patch embedding is obtained by residual normalization:

$$\text{z}_{\text{i}}\text{=}\text{LayerNorm}\text{(}\text{Q}_{\text{i}}\text{+}\text{Dropout}\text{(}\text{m}_{\text{i}}\text{))}$$

The output topology-regularized patch embedding matrix is:

$$\text{Z}\text{=\{}\text{z}_{\text{1}}\text{,}\text{z}_{\text{2}}\text{,…,}\text{z}_{\text{I}}\text{\}∈}\text{R}^{\text{I}\text{×}\text{D}}$$

**1.5 Task-specific Attention Pooling and Residual Task Adapter**

In the joint multi-task setting, SparseAGE-MTL applies task-specific attention pooling to the shared topology-regularized patch embeddings. For each endpoint $\text{m}\text{∈}\text{M}$, the attention score is defined as:

$$\text{e}_{\text{i}}^{\text{(}\text{m}\text{)}}\text{=}\text{w}_{\text{2,}\text{m}}^{\text{T}}\tanh\text{(}\text{W}_{\text{1,}\text{m}}\text{z}_{\text{i}}\text{+}\text{b}_{\text{1,}\text{m}}\text{)+}\text{b}_{\text{2,}\text{m}}$$

The patch attention weights for task $\text{m}$ are:

$$\text{a}_{\text{i}}^{\text{(}\text{m}\text{)}}\text{=}\text{softmax}_{\text{i}}\text{(}\text{e}_{\text{i}}^{\text{(}\text{m}\text{)}}\text{)}$$

The corresponding task-specific slide embedding is:

$$\text{M}_{\text{m}}\text{=}\sum_{\text{i}\text{=1}}^{\text{I}} \text{a}_{\text{i}}^{\text{(}\text{m}\text{)}}\text{z}_{\text{i}}$$

To reduce negative transfer across endpoints, each task embedding is further passed into a Residual Task Adapter:

$${\tilde{\text{M}}}_{\text{m}}\text{=}\text{M}_{\text{m}}\text{+}\text{Adapter}_{\text{m}}\text{(}\text{M}_{\text{m}}\text{)}$$

The Residual Task Adapter consists of LayerNorm, a linear layer, ReLU activation, dropout, and a second linear layer. The adapted embedding ${\tilde{\text{M}}}_{\text{m}}$ is then passed into the corresponding endpoint-specific output head.

**1.6 Endpoint-specific Output Heads and Loss Functions**

SparseAGE-MTL uses endpoint-specific output heads for subtype classification, stage prediction, and survival risk estimation.

Endpoint targets were defined as follows. For subtype classification, the target was the slide-level histological subtype, encoded as LUAD versus LUSC. For stage prediction, the target was pathological stage I-IV, encoded as $\text{y}_{\text{stage}}\text{∈\{0,1,2,3\}}$ and corresponding to stages I, II, III, and IV. Stage prediction metrics were reported separately for LUAD and LUSC to avoid mixing subtype-specific stage distributions in interpretation. For survival risk estimation, the target consisted of OS time and censoring status. OS time was discretized into $\text{T}$ bins using the training split only, and the resulting discrete-time labels were applied unchanged to validation and external cohorts. Survival metrics and risk stratification were reported separately for LUAD and LUSC.

For LUAD/LUSC subtype classification, the model computes logits based on the adapted subtype embedding:

$$\text{o}^{\text{sub}}\text{=}{\tilde{\text{M}}}_{\text{sub}}\text{W}_{\text{sub}}\text{+}\text{b}_{\text{sub}}$$

Class probabilities are obtained by softmax:

$$\text{p}^{\text{sub}}\text{=}\text{softmax}\text{(}\text{o}^{\text{sub}}\text{)}$$

The subtype loss uses cross-entropy:

$$\text{L}_{\text{sub}}\text{=}\text{CE}\text{(}\text{y}_{\text{sub}}\text{,}\text{p}^{\text{sub}}\text{)}$$

For pathological stage prediction, the model outputs four-class logits corresponding to stages I-IV:

$$\text{o}^{\text{stage}}\text{=}{\tilde{\text{M}}}_{\text{stage}}\text{W}_{\text{stage}}\text{+}\text{b}_{\text{stage}}$$

$$\text{p}^{\text{stage}}\text{=}\text{softmax}\text{(}\text{o}^{\text{stage}}\text{)}$$

The stage loss consists of cross-entropy and an ordinal-distance penalty. Given the encoded stage label $\text{y}_{\text{stage}}\text{∈\{0,1,2,3\}}$, the predicted expected stage is:

$$\bar{\text{s}}\text{=}\sum_{\text{c}\text{=0}}^{\text{3}} \text{c}\text{ }\text{p}_{\text{c}}^{\text{stage}}$$

The ordinal-aware stage loss is defined as:

$$\text{L}_{\text{stage}}\text{=}\text{CE}\text{(}\text{y}_{\text{stage}}\text{,}\text{p}^{\text{stage}}\text{)+}\text{λ}_{\text{ord}}\text{Smoot}\text{h}\text{L}\text{1(}\bar{\text{s}}\text{/3,}\text{y}_{\text{stage}}\text{/3)}$$

The default ordinal weight is $\text{λ}_{\text{ord}}\text{=0.15}$.

For discrete-time survival prediction, the survival head outputs conditional hazards for $\text{T}$ discrete time intervals [2]. In the code, the survival-bin label uses 0-based indexing; therefore, $\text{t}\text{=0,…,}\text{T}\text{-1}$:

$$\text{o}^{\text{surv}}\text{=}{\tilde{\text{M}}}_{\text{surv}}\text{W}_{\text{surv}}\text{+}\text{b}_{\text{surv}}$$

$$\text{h}_{\text{t}}\text{=}\text{σ}\text{(}\text{o}_{\text{t}}^{\text{surv}}\text{), }\text{t}\text{=0,…,}\text{T}\text{-1}$$

Define $\text{S}_{\text{0}}\text{=1}$. The survival probability before the start of interval $\text{t}$ is:

$$\text{S}_{\text{t}}\text{=}\prod_{\text{u}\text{=0}}^{\text{t}\text{-1}} \text{(}\text{1-}\text{h}_{\text{u}}\text{)}$$

The survival probability after the end of interval $\text{t}$ is:

$$\text{S}_{\text{t}\text{+1}}\text{=}\text{S}_{\text{t}}\text{(1-}\text{h}_{\text{t}}\text{)}$$

The slide-level risk score is defined as the negative sum of predicted survival probabilities:

$$\text{r}\text{=-}\sum_{\text{t}\text{=1}}^{\text{T}} \text{S}_{\text{t}}$$

Thus, a larger $\text{r}$ indicates a higher predicted risk. For survival-bin label $\text{y}\text{∈\{0,…,}\text{T}\text{-1\}}$ and censoring indicator $\text{c}$, where $\text{c}\text{=0}$ indicates that the event occurred and $\text{c}\text{=1}$ indicates censoring, the discrete-time survival negative log-likelihood is defined as:

$$\text{L}_{\text{surv}}\text{=-(1-}\text{c}\text{)[}\log\text{(}\text{S}_{\text{y}}\text{)+}\log\text{(}\text{h}_{\text{y}}\text{)]-}\text{c}\log\text{(}\text{S}_{\text{y}\text{+1}}\text{)}$$

In addition to endpoint losses, the reconstructed model contains a lightweight topology auxiliary head. When patch coordinates and pseudo-histology cluster labels are available, the model computes a six-dimensional topology descriptor summarizing cluster entropy, dominant-cluster fraction, normalized same-cluster connected-component count, cross-cluster boundary ratio, spatial dispersion, and spatial coverage. The topology descriptor loss is defined as:

$$\text{L}_{\text{topo}}^{\text{raw}}\text{=}\text{Smoot}\text{h}\text{L}\text{1(}\hat{\text{q}}\text{,}\text{q}\text{)+}\text{λ}_{\text{cum}}\text{Smoot}\text{h}\text{L}\text{1(}\text{Cumsum}\text{(}\hat{\text{q}}\text{),}\text{Cumsum}\text{(}\text{q}\text{))}$$

The topology term actually added to the multi-task loss during training is:

$$\text{L}_{\text{topo}}\text{=}\text{λ}_{\text{topo}}\text{L}_{\text{topo}}^{\text{raw}}$$

The default topology loss weight is $\text{λ}_{\text{topo}}\text{=0.10}$, and the default cumulative-shape matching weight is $\text{λ}_{\text{cum}}\text{=0.25}$.

The total joint multi-task loss uses homoscedastic uncertainty weighting by default [3]. To align this formulation with missing-label masking, let $\text{M}_{\text{valid}}\text{⊆\{}\text{sub}\text{,}\text{stage}\text{,}\text{surv}\text{,}\text{topo}\text{\}}$ denote the loss terms with valid endpoint labels or valid topology targets for the current slide. The total loss is written as:

$$\text{L}_{\text{total}}\text{=}\sum_{\text{m}\text{∈}\text{M}_{\text{valid}}} \text{\{}\text{exp}\text{(-}\text{s}_{\text{m}}\text{)}\text{L}_{\text{m}}\text{+}\text{s}_{\text{m}}\text{\}}$$

Here, $\text{s}_{\text{m}}$ is the learnable log-variance parameter for task $\text{m}$. For an endpoint with a missing label, the corresponding loss term is excluded from $\text{M}_{\text{valid}}$ and therefore does not contribute to the loss calculation for that slide. As long as at least one valid endpoint label or topology target exists in a batch, the batch can be used for optimization. This formulation describes only loss aggregation and missing-label masking; the endpoint-specific losses are defined above as subtype loss, stage loss, survival loss, and topology auxiliary loss.

**1.7 Experimental setting**

Model training used the Adam optimizer [4], with an initial learning rate of $\text{2×1}\text{0}^{\text{-4}}$ and weight decay of $\text{1×1}\text{0}^{\text{-5}}$. The learning-rate schedule used cosine annealing. All models were trained for 200 epochs, the batch size was fixed at 1, and the default random seed was 2021. SparseAGE-MTL was evaluated using seven pretrained feature extractors, including CONCH, Virchow2, H-optimus-1, UNI2-h, PLIP, UNI, and ResNet-50 [5–11]. This feature extractor set covered a general natural-image CNN baseline, pathology vision-language foundation models, and large-scale pathology foundation models. All backbones were used as frozen feature extractors and compared under the same patching, tissue filtering, data folds, endpoint labels, and evaluation metrics.

In the default joint multi-task configuration, the model used the Top-K set $\text{K}\text{=\{4,8,16\}}$, embedding dimension $\text{D}\text{=512}$, attention dimension 128, dropout rate 0.25, and standard gradient aggregation. PCGrad was implemented as an optional ablation method [12], but it was not used in the default configuration. Survival time bins were fitted only on the training split and then applied unchanged to the validation split and external cohorts to avoid survival discretization leakage. Mixed precision was not enabled in the default configuration. Training outputs included the resolved configuration, train/validation manifests, metrics, the best checkpoint, and slide-level prediction files. Because the MIL framework needs to load and process all valid instances from a complete WSI within one forward pass, GPU memory consumption is high; therefore, the batch size was fixed at 1. All deep learning experiments were performed on NVIDIA GPUs.

**2 Comprehensive Benchmarking, Paired Statistical Comparison, and Feature-Extractor Contribution Analysis**

Based on the benchmark settings defined in the main-text Methods, we systematically compared seven pretrained feature extractors and 19 models. The included feature extractors were CONCH, Virchow2, H-optimus-1, UNI2-h, PLIP, UNI, and ResNet-50 [5–11]. The 11 endpoint-level metrics included accuracy, AUC, and F1-score for NSCLC subtype classification; accuracy, AUC, and F1-score for LUAD stage prediction; accuracy, AUC, and F1-score for LUSC stage prediction; and the C-index for LUAD and LUSC survival prediction. All benchmark summaries were based on matched five-fold cross-validation results, using the same folds, labels, feature files, and evaluation metrics across models.

For cross-model benchmark comparison, we first constructed a performance matrix with 19 models and 77 feature-endpoint-metric settings. Model performance within each setting was processed by min-max normalization:

$$\text{s}_{\text{m}\text{,}\text{j}}^{\text{norm}}\text{=(}\text{s}_{\text{m}\text{,}\text{j}}\text{-}\min_{\text{m}} \text{s}_{\text{m}\text{,}\text{j}}\text{)/(}\max_{\text{m}} \text{s}_{\text{m}\text{,}\text{j}}\text{-}\min_{\text{m}} \text{s}_{\text{m}\text{,}\text{j}}\text{)}$$

When all model performance values within a setting were identical, the denominator was set to 1. Model rank was then calculated within each setting, and the mean rank across all settings was used to represent the overall model ordering:

$${\bar{\text{r}}}_{\text{m}}\text{=7}\text{7}^{\text{-1}}\sum_{\text{j}\text{=1}}^{\text{77}} \text{r}_{\text{m}\text{,}\text{j}}$$

For CONCH-based benchmark uncertainty analysis, five-fold mean performance and 95% CI were used to summarize the performance of each model across the 11 endpoint-level metrics. For paired comparison, we selected the non-SparseAGE baseline with the highest mean performance within each feature-endpoint-metric setting and calculated the matched fold-level paired difference between the target model and this best baseline. The paired difference was defined as:

$$\text{Δ}_{\text{f}\text{,}\text{e}\text{,}\text{k}}=\text{s}_{\text{target}\text{,}\text{f}\text{,}\text{e}\text{,}\text{k}}-\text{s}_{\text{bestbaseline}\text{,}\text{f}\text{,}\text{e}\text{,}\text{k}}$$

Here, $\text{k}$ denotes the cross-validation fold. Bootstrap resampling was used to estimate the 95% CI of the mean paired difference [13]. In fold-level sensitivity analysis, the exact sign-flip test was used to assess whether paired differences deviated from 0, and Benjamini-Hochberg FDR correction was applied for multiple testing.

For feature-extractor contribution analysis, we first calculated the global mean score of each feature extractor across all models and endpoint-level metrics:

$$G_{f}=\left( MN \right)^{-1}\sum_{m=1}^{M} \sum_{e=1}^{N} s_{f,m,e}$$

Because the absolute numerical ranges differed across endpoint-level metrics, we further performed endpoint-normalized feature sensitivity analysis. Specifically, within each endpoint, the mean performance of the seven feature extractors was min-max normalized to obtain the endpoint-normalized score:

$$\text{z}_{\text{f}\text{,}\text{e}}\text{=(}\text{s}_{\text{f}\text{,}\text{e}}\text{-}\min_{\text{f}} \text{s}_{\text{f}\text{,}\text{e}}\text{)/(}\max_{\text{f}} \text{s}_{\text{f}\text{,}\text{e}}\text{-}\min_{\text{f}} \text{s}_{\text{f}\text{,}\text{e}}\text{)}$$

When all feature extractors had identical performance values within an endpoint, the denominator was set to 1. This analysis was performed separately for SparseAGE-MTL and SparseAGE-MIL, and the mean value and 95% CI of each feature extractor were calculated based on fold-level normalized scores.

To distinguish the relative effects of MIL architecture and feature extractor on benchmark performance, we further performed two complementary comparisons. The first was feature-fixed architecture comparison, in which the CONCH feature space was fixed and different MIL architectures were compared using endpoint-normalized scores across endpoint-level metrics. The second was architecture-fixed feature comparison, in which the SparseAGE-MIL architecture was fixed and different feature extractors were compared using endpoint-normalized scores across endpoint-level metrics. Finally, approximate variance contribution analysis was performed based on endpoint-normalized benchmark scores. For each factor, an approximate sum of squares was calculated as:

$$\text{S}\text{S}_{\text{factor}}\text{=}\sum_{\text{g}} \text{n}_{\text{g}}\text{(}{\bar{\text{z}}}_{\text{g}}\text{-}\bar{\text{z}}\text{)}^{\text{2}}$$

Here, $\text{g}$ denotes the levels of the corresponding factor, $\text{n}_{\text{g}}$ denotes the sample size of level $\text{g}$, ${\bar{\text{z}}}_{\text{g}}$ denotes the mean endpoint-normalized score of that level, and $\bar{\text{z}}$ denotes the grand mean. Relative contribution was defined as:

$$\text{Contributio}\text{n}_{\text{factor}}\text{=}\text{S}\text{S}_{\text{factor}}\text{/(}\text{S}\text{S}_{\text{model}}\text{+}\text{S}\text{S}_{\text{feature}}\text{+}\text{S}\text{S}_{\text{endpoint}}\text{+}\text{S}\text{S}_{\text{residual}}\text{)}$$

This analysis approximately decomposed variation in benchmark performance into four sources: MIL architecture, feature extractor, endpoint, and residual/interactions.

For patient-level statistical comparison, AUC differences in classification tasks were evaluated using the DeLong test [14], and the 95% CI of AUC difference was estimated through bootstrap resampling. For survival prediction, C-index differences were assessed using patient-level bootstrap resampling and summarized with bootstrap 95% CIs.

**3 Integrated Spatial Analysis of Spatial Transcriptomics and Attention Heatmaps**

**3.1 Spatial transcriptomics**

**3.1.1 Cell Type and Functional Zone Annotation**

Raw sequencing data underwent spatial coordinate positioning and tissue section alignment via Space Ranger to generate spatial gene expression matrices. During quality control (QC), we excluded spots with fewer than 200 detected genes or a mitochondrial gene proportion exceeding 10% to filter out low-quality data and potential artifacts. Qualified data were standardized, including gene name unification, total expression normalization, log transformation, and highly variable gene (HVG) selection.

For the Visium data, gene symbols were first unified to uppercase and matched against a marker list, retaining only marker genes with detectable signals in the current dataset. We then calculated marker gene scores for each spot and candidate Cell Subtype. Let $s$ be a Visium spot, $c$ a candidate Cell Subtype, ${\mathcal{\mathcal{M}}}_{c}^{*}$ the set of marker genes for that subtype actually detected in the dataset, ${\mathcal{\mathcal{B}}}_{c}$ a set of background genes matched to the expression levels of ${\mathcal{\mathcal{M}}}_{c}^{*}$, and $x_{s,g}$ the expression of gene $g$ in spot $s$; the marker score for the subtype in that spot is defined as:

$$\mathrm{Score}(s,c)=\frac{1}{|{\mathcal{\mathcal{M}}}_{c}^{*}|}\sum_{g\in{\mathcal{\mathcal{M}}}_{c}^{*}} x_{s,g} - \frac{1}{|{\mathcal{\mathcal{B}}}_{c}|}\sum_{g\in{\mathcal{\mathcal{B}}}_{c}} x_{s,g}.$$

For each spot $s$, the Cell Subtype with the highest score among all candidate subtypes $\mathcal{C}$ was selected as the primary Cell Subtype:

$$c^{*}(s)=\arg\max_{c\in\mathcal{C}}\mathrm{Score}(s,c).$$

We further merged these Cell Subtypes into higher-level Cell Lineages according to pre-defined mapping relationships. Specifically, all lung epithelial-related clusters were categorized as "Lung epithelial cells," lung epithelial tumor-related clusters as "Lung epithelial tumor cells," B-cell clusters as "B cells," other immune cell clusters as "Non-B immune cells," endothelial cell clusters as "Endothelial cells," and fibroblast and smooth muscle-related clusters as "Fibroblasts and smooth muscle cells".

To reveal the Functional State of various cells across different spatial regions, we performed functional annotation on the Visium data using functional gene sets from the CancerSEA database. The covered functional modules included Proliferation, Stemness, Invasion, Inflammatory response, Cell cycle, Apoptosis, and DNA repair. Similar to the Cell Subtype annotation process, we calculated expression scores for each functional module per spot, labeling the module with the highest score as the primary Functional State to resolve the spatial distribution and differences in functional states within the tumor microenvironment.

Finally, Cell Subtype, Cell Lineage, and Functional State were mapped back to the spatial coordinates of the tissue sections and visualized using multi-color labeling to intuitively present spatial trends in functional states.

**3.1.2 Reliability of Cell Type Annotation**

To validate the Cell Subtype annotation results derived from marker gene scoring, this study further selected typical subtype-specific genes for spatial expression pattern comparison. Using the Alveolar cell type 2 specific marker gene *SFTPC* as an example, we visualized its spatial distribution and performed a consistency check against the Alveolar cell type 2 spatial annotations. We compared whether the enrichment regions, boundary contours, and local clustering features of both were mutually consistent, providing evidence for the reliability of the subtype labeling.

To comprehensively present the overall structure and interrelationships of different cell populations in the transcriptomic feature space, we performed non-linear dimensionality reduction and visualization. Specifically, a low-dimensional embedding was constructed from the high-dimensional gene expression matrix using UMAP. Cell Subtype and Cell Lineage annotations were then projected onto this embedding to demonstrate the distribution patterns and clustering structures of major cell categories and their internal sub-clusters within a unified 2D coordinate system.

**3.1.3 Peritumoral Tissue Analysis**

To quantitatively characterize the microenvironmental gradient between the tumor core, edge, and peripheral tissue at both the physical distance and molecular feature levels, this study developed a neighborhood search strategy based on Visium spatial coordinates to define and analyze peritumoral regions. First, spots annotated as "Lung epithelial tumor cells" were defined as the tumor core. A spatial index was established using their 2D coordinates, and a neighborhood radius of 250 $\mu m$ was set. Using the chip’s spatial calibration parameters, this physical distance was converted into pixel scales to retrieve all neighboring spots within 250 $\mu m$ for every tumor spot. Furthermore, a threshold for the number of neighboring tumor cells was set to retain only those regions where the local tumor cell count met a minimum requirement, ensuring spatial continuity and reducing noise from isolated points.

Based on these spatial neighborhood determinations, we constructed simplified spatial partition labels within the sample metadata: spots enriched with tumor cells meeting the continuity criteria were defined as "Tumor" regions; non-tumor spots within a 250 $\mu m$ radius of these regions were defined as "250 micron" peritumoral zones; and all other tissue spots were categorized as "Tissue" background zones. This partitioning strategy enabled a stratified comparative analysis of the tumor core, the proximal periphery, and more distal tissues within a unified framework.

In the spatial gene expression analysis of the tumor and its surrounding areas, we focused on the expression patterns of *C1QC* and *COL1A1* to reflect the co-localization features of immune cells and stromal components at the tumor margin. First, we visualized the expression of *C1QC* and *COL1A1* across the three spatial zones (Tumor / 250 micron / Tissue) for qualitative comparison. Subsequently, we extracted expression levels for these genes within each zone and utilized the Mann–Whitney U test to compare expression differences between the 250 $\mu m$ peritumoral zone, the tumor core, and the distal tissue, thereby quantitatively evaluating their enrichment at the tumor edge.

**3.2 Attention Heatmap Visualization**

Following the completion of NSCLC subtype classification, pathological stage prediction, and survival risk estimation tasks, we performed interpretability analysis on the trained SparseAGE-MTL model. In the joint multi-task setting, attention weights are endpoint-specific because each endpoint has an independent task-specific attention pooling module. Therefore, attention heatmaps were generated from the corresponding endpoint attention weights. For survival-oriented spatial interpretation, survival head attention was preferentially used; for subtype-oriented interpretation, subtype head attention was used.

For each WSI and endpoint $\text{m}$, the attention module outputs an importance score for each valid instance patch:

$$\text{a}_{\text{i}}^{\text{(}\text{m}\text{)}}\text{∈[0,1], }\text{i}\text{=1,2,…,}\text{I}$$

where $\text{I}$ is the number of valid patches in the slide and $\text{m}\text{∈}\text{M}$ denotes the endpoint. These scores were mapped back to the original WSI coordinates and normalized to obtain saliency values for each patch in tissue space. Finally, patch-level scores were converted into a continuous distribution across pixel space and color-coded according to attention intensity to generate the full-slide attention heatmap.

For spatial transcriptomics-based quantitative validation of attention hotspots, patch-level attention scores were projected onto Visium spot coordinates, and spots were retained only if they had attention assignment, spatial region annotation, and ST-derived features simultaneously. Spatial regions included tumor-dense, tumor-stroma/immune interface, stroma/immune-rich, and other tissue regions. Attention score distributions across spatial regions were compared using the Kruskal-Wallis test, with FDR-adjusted pairwise comparisons performed when needed. Top-attention spots were defined as the top 10% of spots ranked by attention score. For each spatial region, the enrichment ratio was defined as the ratio between the observed top-attention spot count and the expected count, where the expected count was calculated based on the proportion of that region among all valid spots. Top-attention enrichment was evaluated using Fisher's exact test with FDR correction. Correlations between attention scores and ST-derived cell-type scores, functional state scores, and local neighborhood composition features were calculated using Spearman correlation, with FDR correction applied across all tested features.

**3.3 Analysis of Attention Hotspots Driven by Spatial LUSC Cell Subpopulation Composition**

During spatial comparison of the model-generated attention heatmaps, we observed that high-attention hotspots generally overlapped with spatial regions containing high LUSC cell density; however, the model assigned varying attention weights even among different regions similarly enriched with LUSC cells. To systematically analyze this phenomenon, we returned to the cell subtype level to provide a biological explanation for the model’s spatial attention patterns, focusing on LUSC subpopulation composition and molecular functional characteristics.

First, we extracted all spots labeled as LUSC cells from the spatial transcriptomics data to construct an LUSC cell subset based on previous annotations. We then integrated the model's attention weight information to stratify the LUSC-enriched regions: tumor regions with high attention weights were defined as "High-priority tumor spots," while those with relatively lower attention weights were defined as "Low-priority tumor spots". Spatial visualization revealed distinct distribution patterns for these two categories, suggesting the model prioritizes specific spatial sub-regions within the same tumor cell class.

To quantitatively compare the molecular differences between High-priority and Low-priority tumor spots, we performed differential expression analysis within the LUSC cell subset based on these spatial priority labels. Subsequently, we conducted GO-Biological Process (BP), KEGG pathway, and GSEA analyses on the differentially expressed genes to characterize functional enrichment in areas such as cell proliferation, invasion and migration, epithelial-mesenchymal transition (EMT), metabolic reprogramming, and immune-related pathways. This process revealed the specific LUSC cell functional states corresponding to high-attention regions in the model.

**References**

[1] Kanavati F, Toyokawa G, Momosaki S, et al. Weakly-supervised learning for lung carcinoma classification using deep learning. Sci Rep 2020;10:9297. https://doi.org/10.1038/s41598-020-66333-x.

[2] Kvamme H, Borgan Ø, Scheel I. Time-to-Event Prediction with Neural Networks and Cox Regression. Journal of Machine Learning Research 2019;20:1–30.

[3] Kendall A, Gal Y, Cipolla R. Multi-Task Learning Using Uncertainty to Weigh Losses for Scene Geometry and Semantics 2018. https://doi.org/10.48550/arXiv.1705.07115.

[4] Kinga D, Adam JB. A method for stochastic optimization. vol. 5, California; 2015.

[5] Lu MY, Chen B, Williamson DF, et al. A visual-language foundation model for computational pathology. Nature Medicine 2024;30:863–74.

[6] Zimmermann E, Vorontsov E, Viret J, et al. Virchow2: Scaling Self-Supervised Mixed Magnification Models in Pathology 2024. https://doi.org/10.48550/arXiv.2408.00738.

[7] Scalbert M, Saillard C, Peeters T, et al. Abstract LB174: H-optimus-1: A foundation model for computational histopathology. Cancer Research 2026;86:LB174–LB174. https://doi.org/10.1158/1538-7445.AM2026-LB174.

[8] mahmoodlab/UNI 2026.

[9] Huang Z, Bianchi F, Yuksekgonul M, et al. A visual–language foundation model for pathology image analysis using medical twitter. Nature Medicine 2023;29:2307–16. https://doi.org/10.1038/s41591-023-02504-3.

[10] Chen RJ, Ding T, Lu MY, et al. Towards a general-purpose foundation model for computational pathology. Nature Medicine 2024;30:850–62.

[11] He K, Zhang X, Ren S, et al. Deep residual learning for image recognition. Proceedings of the IEEE conference on computer vision and pattern recognition, 2016, p. 770–8.

[12] Yu T, Kumar S, Gupta A, et al. Gradient Surgery for Multi-Task Learning 2020. https://doi.org/10.48550/arXiv.2001.06782.

[13] Efron B. Bootstrap Methods: Another Look at the Jackknife. In: Kotz S, Johnson NL, editors. Breakthroughs in Statistics: Methodology and Distribution, New York, NY: Springer; 1992, p. 569–93. https://doi.org/10.1007/978-1-4612-4380-9_41.

[14] Molodianovitch K, Faraggi D, Reiser B. Comparing the Areas Under Two Correlated ROC Curves: Parametric and Non-Parametric Approaches. Biometrical Journal 2006;48:745–57. https://doi.org/10.1002/bimj.200610223.

Supplementary figures

**Supplementary Figure 1. Five-fold cross-validation uncertainty of CONCH-based benchmark performance.**

This figure summarizes CONCH feature space-based model comparison results across 11 endpoint-level metrics, including subtype accuracy, subtype AUC, subtype F1-score, LUAD stage accuracy, LUAD stage AUC, LUAD stage F1-score, LUSC stage accuracy, LUSC stage AUC, LUSC stage F1-score, LUAD C-index, and LUSC C-index. Each point represents the five-fold mean performance of the corresponding model, and horizontal error bars represent 95% CIs. SparseAGE-MTL, the non-MTL comparator SparseAGE-MIL, and other non-SparseAGE MIL baselines are distinguished by different colors.

**Supplementary Figure 2. Fold-level paired differences between SparseAGE-MIL and the best non-SparseAGE baseline.**

The forest plot shows matched five-fold paired differences between the non-MTL comparator SparseAGE-MIL and the best non-SparseAGE baseline. The best non-SparseAGE baseline was selected independently within each feature-endpoint-metric setting according to mean performance. Each row corresponds to one endpoint, feature extractor, and best-baseline comparison. Points indicate the five-fold mean paired difference, horizontal bars indicate bootstrap 95% CIs, and the vertical dashed line indicates zero difference. Colors indicate endpoint categories, including subtype classification, LUAD stage prediction, LUSC stage prediction, LUAD survival prediction, and LUSC survival prediction.

**Supplementary Figure 3. Feature-extractor sensitivity analysis in SparseAGE-MTL and SparseAGE-MIL.**

(A) Endpoint-normalized feature sensitivity heatmap and mean endpoint-normalized score summary for SparseAGE-MTL. The heatmap shows endpoint-normalized scores of seven pretrained feature extractors across 11 endpoint-level metrics; the dot-and-error-bar plot on the right summarizes the mean value and 95% CI of each feature extractor based on fold-level normalized scores.

(B) Endpoint-normalized feature sensitivity heatmap and mean endpoint-normalized score summary for the non-MTL comparator SparseAGE-MIL. The definitions of the heatmap and dot-and-error-bar plot are the same as in panel A and are used to compare the relative contribution and stability of different feature extractors under a fixed model setting.

**Supplementary Figure 4. Architecture-level and feature-level contribution analysis.**

(A) Feature-fixed architecture comparison. After fixing the CONCH feature space, SparseAGE-MTL, SparseAGE-MIL, and non-SparseAGE MIL baselines were compared across 11 endpoint-level metrics using endpoint-normalized scores. Heatmap colors indicate endpoint-normalized performance, with brighter colors indicating higher relative performance.

(B) Architecture-fixed feature comparison. After fixing the SparseAGE-MIL architecture, seven pretrained feature extractors were compared across 11 endpoint-level metrics using endpoint-normalized scores. Heatmap colors are defined as in panel A.

(C) Approximate variance contribution analysis. Based on endpoint-normalized benchmark scores, performance variation was approximately decomposed into four sources: MIL architecture, feature extractor, endpoint, and residual/interactions. Horizontal bars indicate the approximate variance contribution percentage of each source.

**Supplementary Figure 5. Patient-level classification AUC comparison based on DeLong analysis.**

The forest plot shows patient-level AUC differences between SparseAGE-MIL and the best non-SparseAGE baseline in classification tasks, including subtype classification, LUAD stage prediction, and LUSC stage prediction. Each row corresponds to one task, feature extractor, and best-baseline comparison. Points indicate AUC difference, horizontal bars indicate bootstrap 95% CIs, and the vertical dashed line indicates zero difference. DeLong P values were used for paired patient-level AUC comparison. This analysis included 862 patients for subtype classification, 430 patients for LUAD stage prediction, and 432 patients for LUSC stage prediction.

**Supplementary Figure 6. Patient-level survival C-index comparison based on bootstrap analysis.**

The forest plot shows patient-level C-index differences between SparseAGE-MIL and the best non-SparseAGE baseline in LUAD and LUSC survival prediction. Each row corresponds to one tumor subtype, feature extractor, and best-baseline comparison. Points indicate C-index difference, horizontal bars indicate bootstrap 95% CIs, and the vertical dashed line indicates zero difference. This analysis included 430 LUAD patients and 432 LUSC patients, all with available survival labels.

**Supplementary Figure 7. Additional diagnostics for CONCH-based subtype classification and stage prediction.**

(A) Confusion matrices and prediction-probability analyses for LUAD/LUSC subtype classification in the TCGA training set, TCGA internal validation set, and external SMUZH and SAMSPH cohorts. In each cohort, the left panel shows the confusion matrix, the middle panel shows prediction-probability density distributions stratified by true subtype label, and the right panel shows predicted probability boxplots grouped by true subtype label.

(B) Training and validation curves for the CONCH-based subtype classification model, including loss, accuracy, AUC, and F1-score across training epochs, showing convergence behavior and the train-validation performance gap.

(C) Training and validation curves for CONCH-based LUAD and LUSC stage prediction models, including loss, accuracy, AUC, and F1-score across training epochs.

(D) CONCH-based LUAD and LUSC stage prediction diagnostics in the TCGA training set, TCGA test set, and external SAMSPH cohort. For each tumor subtype and cohort, the panel shows the confusion matrix, prediction-confidence distribution, class-wise sensitivity, and one-vs-rest stage calibration curves. Stage labels 0-3 correspond to pathological stages I-IV. Calibration curves report mean Brier score and mean ECE across stage classes and should be interpreted cautiously because class distribution and external class-specific sample sizes were imbalanced.

(E) Survival probability calibration for LUAD and LUSC in the TCGA training, external SMUZH validation, and external SAMSPH validation cohorts. Calibration was evaluated at representative time points using predicted overall survival probability and observed survival estimates.

**Supplementary Figure 8. Quantitative validation of attention hotspots using spatial transcriptomics-derived features.**

(A) Spatial distribution of attention scores and annotated tissue regions after mapping patch-level attention weights to Visium spots. Only spots with valid attention assignment and spatial transcriptomics annotations were included in the quantitative analysis.

(B) Comparison of normalized attention score distributions across pathologist-guided spatial regions, including tumor-dense, tumor-stroma/immune interface, stroma/immune-rich, and other tissue regions. Only spots with valid attention assignment and ST-derived region annotation were included. Boxplots summarize spot-level attention scores. Group-level differences were evaluated using the Kruskal-Wallis test, followed by FDR-adjusted pairwise comparisons.

(C) Enrichment of the top 10% attention spots across spatial regions. Bars show observed-to-expected enrichment ratios. Labels indicate observed top-attention spot counts, enrichment ratios, and FDR-adjusted Fisher exact test statistics.

(D) Spearman correlation analysis between spot-level attention scores and ST-derived features, including cell-type scores, functional state scores, and local neighborhood composition features. Positive and negative correlations are shown separately, and all displayed q values were adjusted across tested features using Benjamini-Hochberg FDR correction.

(E) Enrichment of the top 10% attention spots in high-priority and low-priority tumor regions. Bars show enrichment ratios, and labels indicate observed spot counts and FDR-adjusted enrichment statistics.

**Supplementary Figure 9. Spot-level composition statistics and spatial maps of Visium-derived annotations in a representative LUAD spatial transcriptomics section.**

(A) Bar plots summarizing the number of Visium spots assigned to each cell subtype, aggregated cell lineage, and dominant functional state derived from CancerSEA gene-set scoring.

(B) Spatial visualization on the matched H&E image. From left to right: original H&E image, model attention heatmap, cell lineage spatial map, functional state spatial map, and fine-grained cell subtype spatial map.

**Supplementary Figure 10. Functional characterization of low-attention tumor spots in spatial transcriptomics.**

(A) Term-gene association heatmap for enriched GO terms, with tiles colored by differential expression direction and the magnitude of representative genes contributing to each term.

(B) Gene-concept network connecting enriched GO terms with their leading-edge genes. Node size reflects gene set size, and node color denotes fold-change direction according to the figure scale.

(C) GO enrichment dot plot for low-attention tumor spots, separated into BP, CC, and MF categories. Dot size indicates the number of genes mapped to each term, and color represents enrichment significance according to the figure scale.

Supplementary table captions

**Supplementary Table 1. Marker gene sets for cell subtype annotation and lineage mapping in Visium spatial transcriptomics**

**Supplementary Table 2. CancerSEA Function GeneSets**

**Supplementary Table 3. Immune cell–specific gene signatures used to characterize the tumor immune microenvironment**

**Supplementary Table 4. Full statistical comparison of immune and stromal cell infiltration scores between model-derived high-risk and low-risk groups.**

For each tumor subtype, immune-infiltration algorithm and cell population, the table reports the high-risk and low-risk group sample sizes, means, medians, high-minus-low differences, standardized mean differences, raw P values and FDR-adjusted q values. Mann-Whitney U tests were used for two-group comparisons based on the original immune-infiltration scores, followed by Benjamini-Hochberg FDR correction across all tested cell-type × algorithm combinations within each tumor subtype.

**Supplementary Table 5. Hyperparameter search spaces and selected configurations for SparseAGE-MTL and comparator models**

This table summarizes the model family, task setting, feature extractor, hyperparameter search space, selection metric, selection procedure, selected configuration, and whether external validation participated in tuning for SparseAGE-MTL, SparseAGE-MIL, and comparator models.

**Supplementary Table 6. Matched five-fold cross-validation benchmark across feature extractors, endpoints, models, and metrics**

This table reports matched five-fold cross-validation performance across seven pretrained feature extractors, 19 models, five task settings, and 11 endpoint-metric settings. Each row corresponds to a feature extractor-model-endpoint-metric combination and includes Fold 1-5, mean, SD, bootstrap 95% CI, and rank within the same setting. Rank within setting was calculated by descending mean score within the same feature extractor, endpoint/task, and metric, with rank = 1 indicating the highest performance in that setting.

**Supplementary Table 7. Fold-level paired comparisons between SparseAGE-MTL, SparseAGE-MIL, and comparator models**

This table reports paired comparisons between SparseAGE-MTL or SparseAGE-MIL and the non-SparseAGE baseline with the highest mean score under the same feature extractor, endpoint, metric, and fold setting. The table includes target mean, comparator mean, mean paired difference, SD of paired difference, bootstrap 95% CI, exact sign-flip P value, Benjamini-Hochberg FDR-adjusted Q value, and Fold 1-5 paired differences.
