# Supplementary figures and images for: An interpretable multi‐task whole‐slide histopathology AI model for non‐small cell lung cancer: Cross‐cohort generalisation, spatial attention–transcriptomic integration, and molecular–immune profiling
Source: Clin Transl Med. 2026 Jul 23;16(7):e70744. doi: 10.1002/ctm2.70744 (PMC13396892; doi:10.1002/ctm2.70744)

# Five-fold mean and 95% CI using CONCH

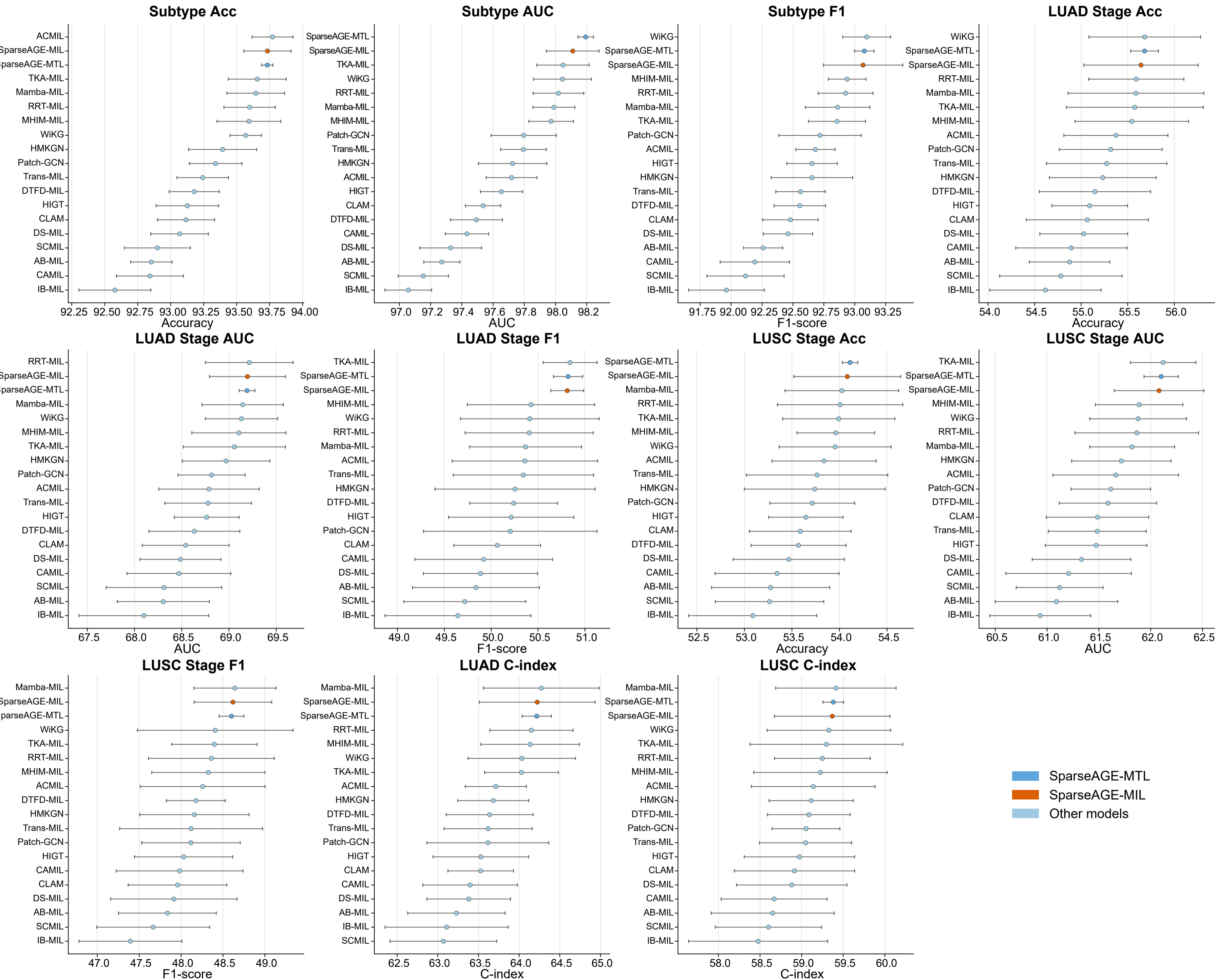

Supplement: Supplementary file 2 — Supporting Information [file CTM2-16-e70744-s006.pdf]

# Fold-level paired differences with bootstrap CI

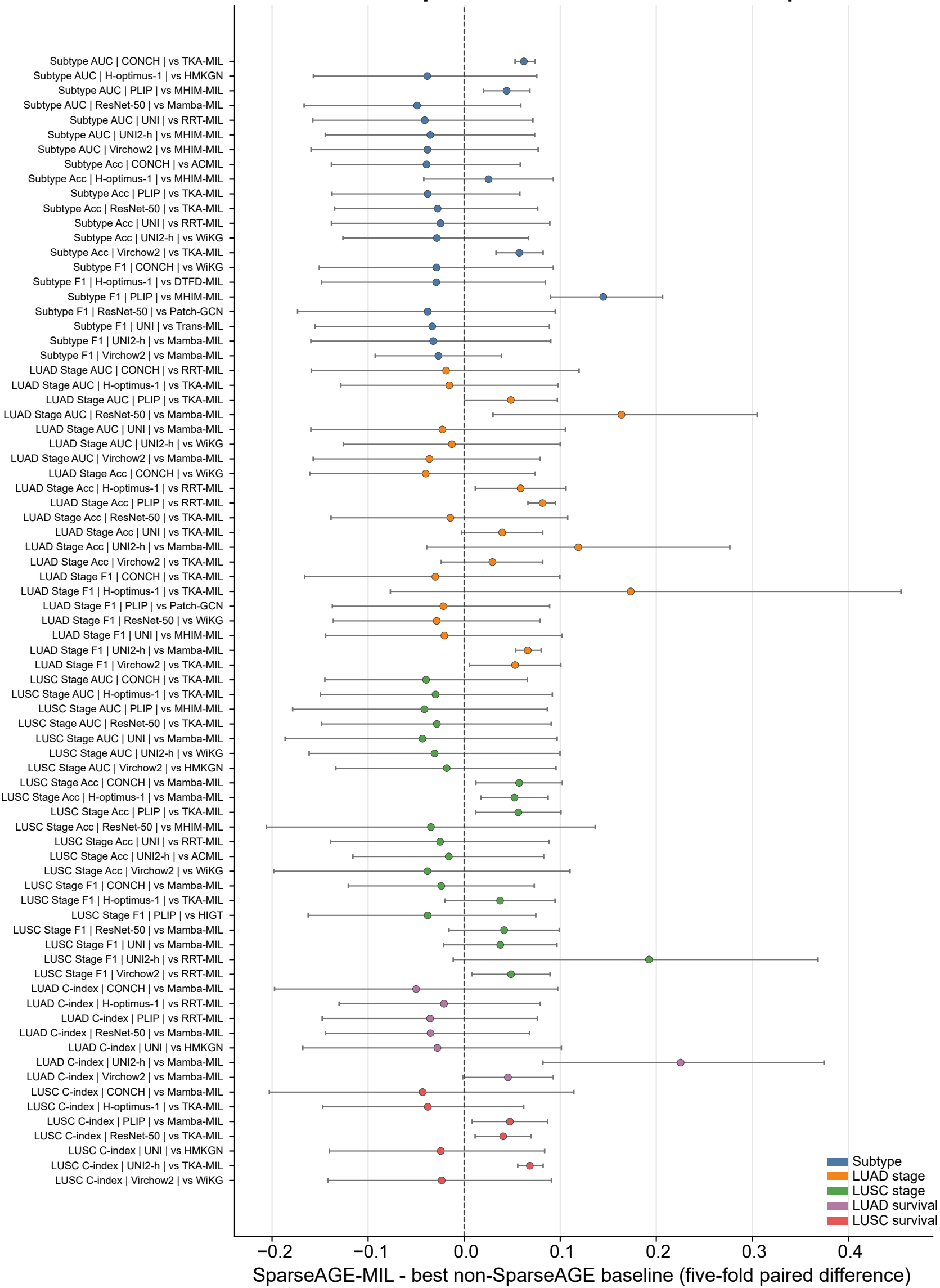

Supplement: Supplementary file 3 — Supporting Information [file CTM2-16-e70744-s009.pdf]

A

Feature sensitivity in SparseAGE-MTL

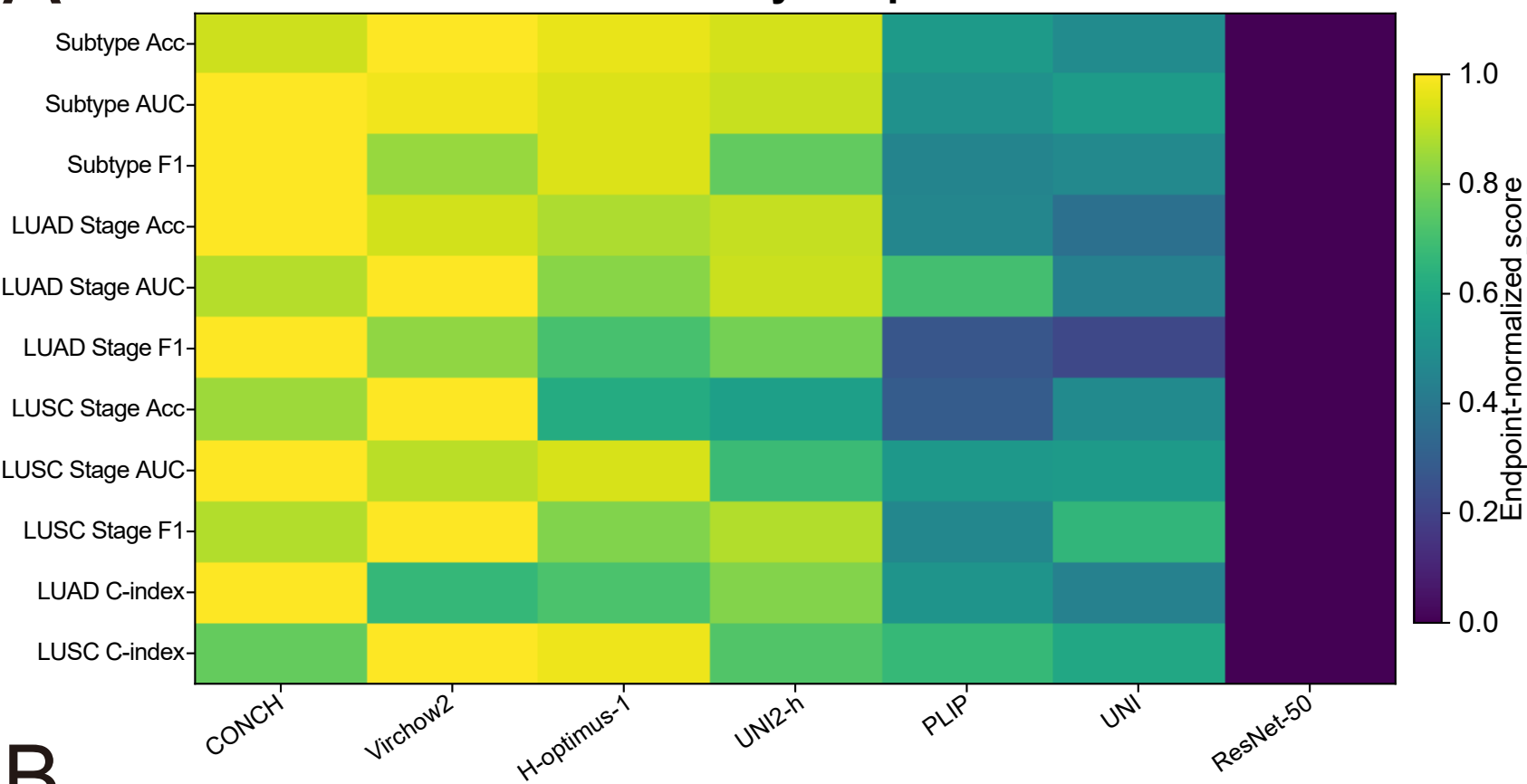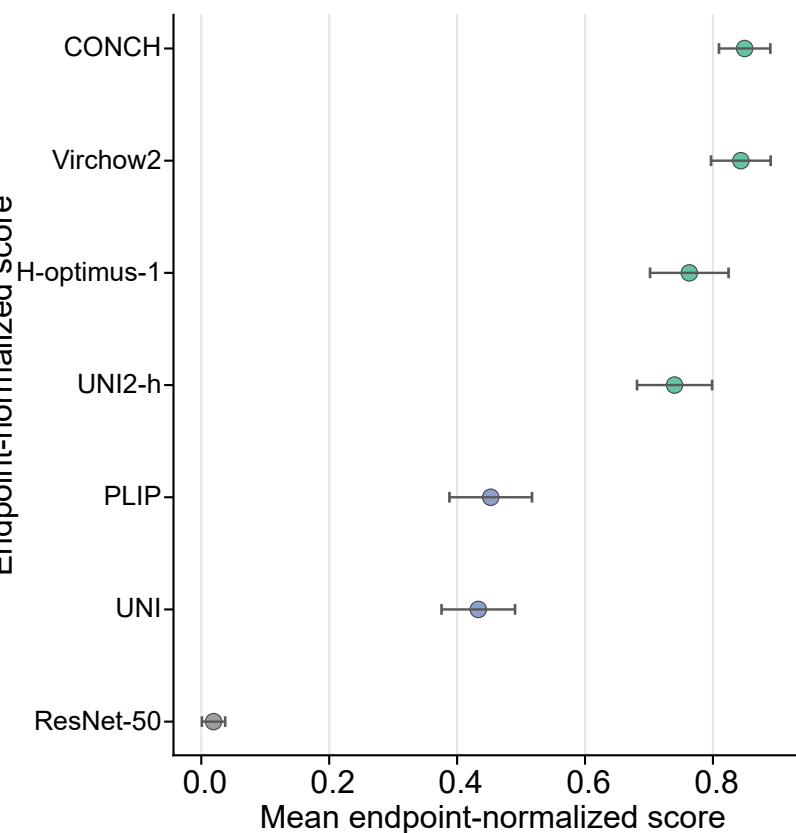

B

Feature sensitivity in SparseAGE-MIL

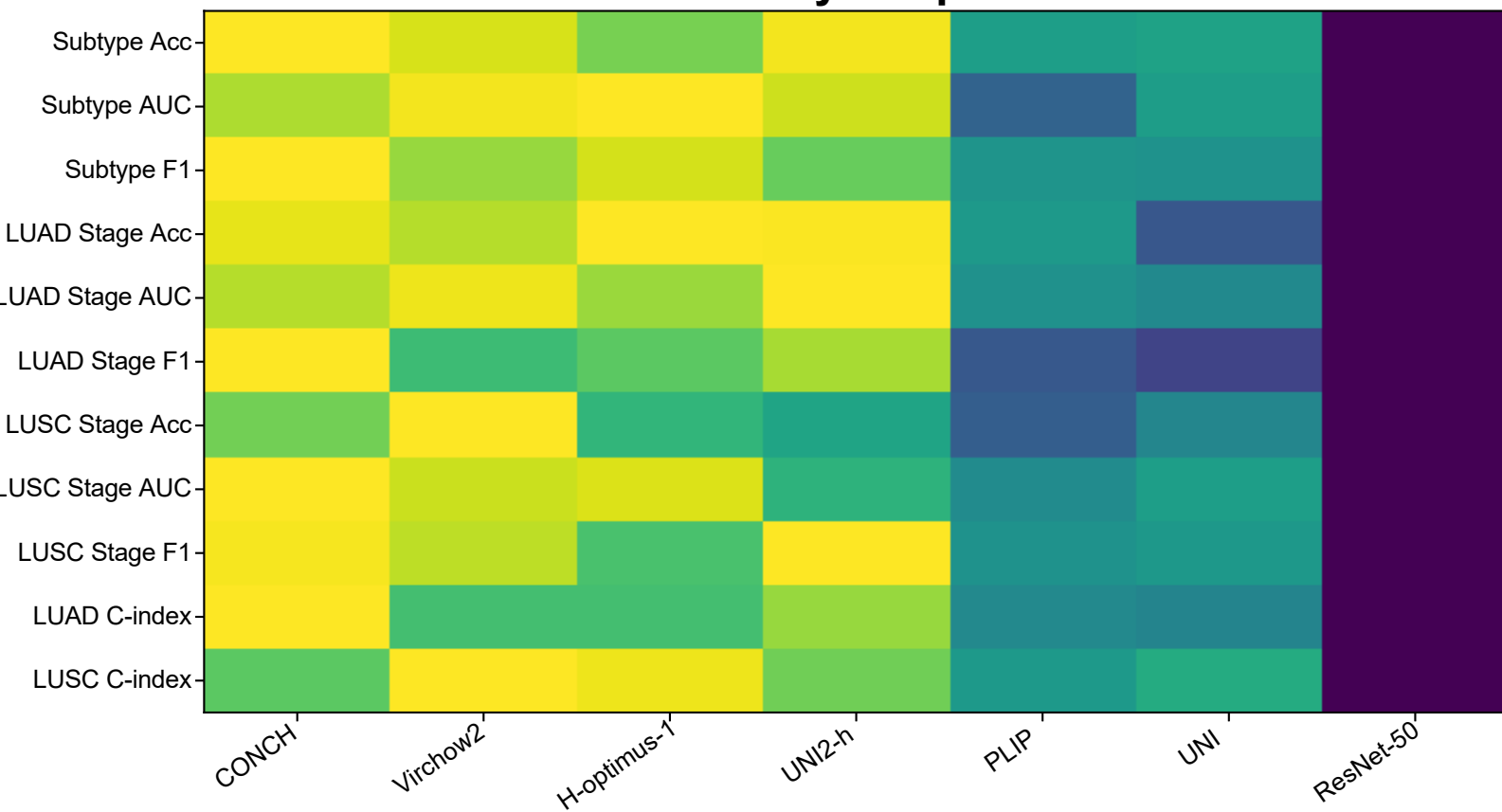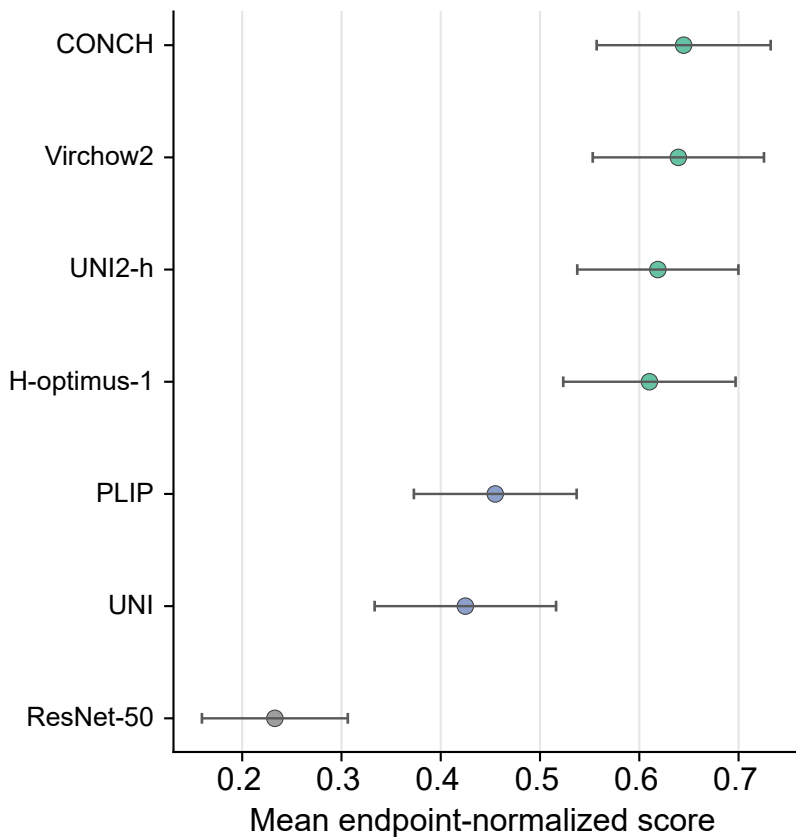

Supplement: Supplementary file 4 — Supporting Information [file CTM2-16-e70744-s014.pdf]

# Classification AUC DeLong analysis

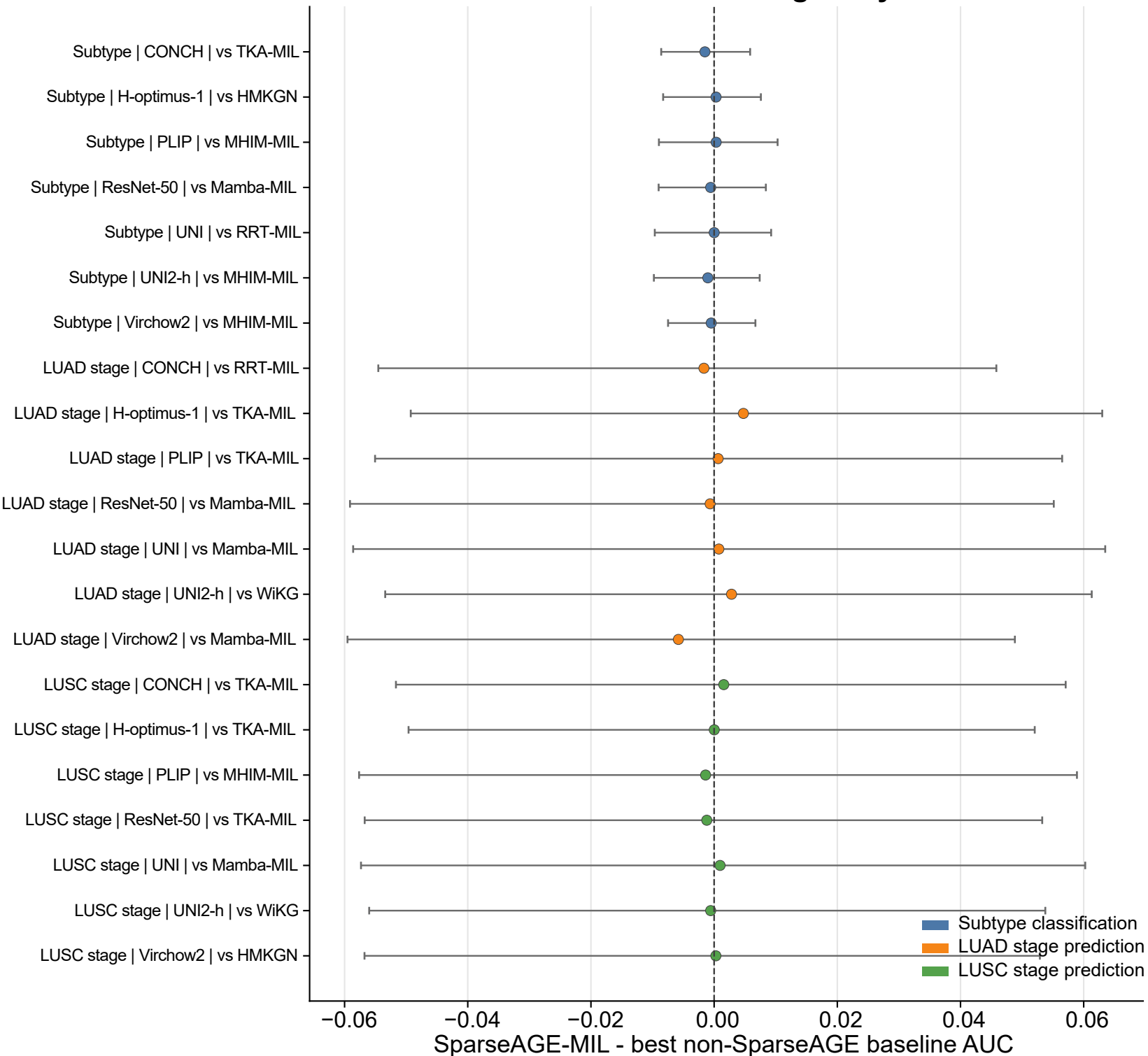

Supplement: Supplementary file 6 — Supporting Information [file CTM2-16-e70744-s008.pdf]

# Survival C-index bootstrap analysis

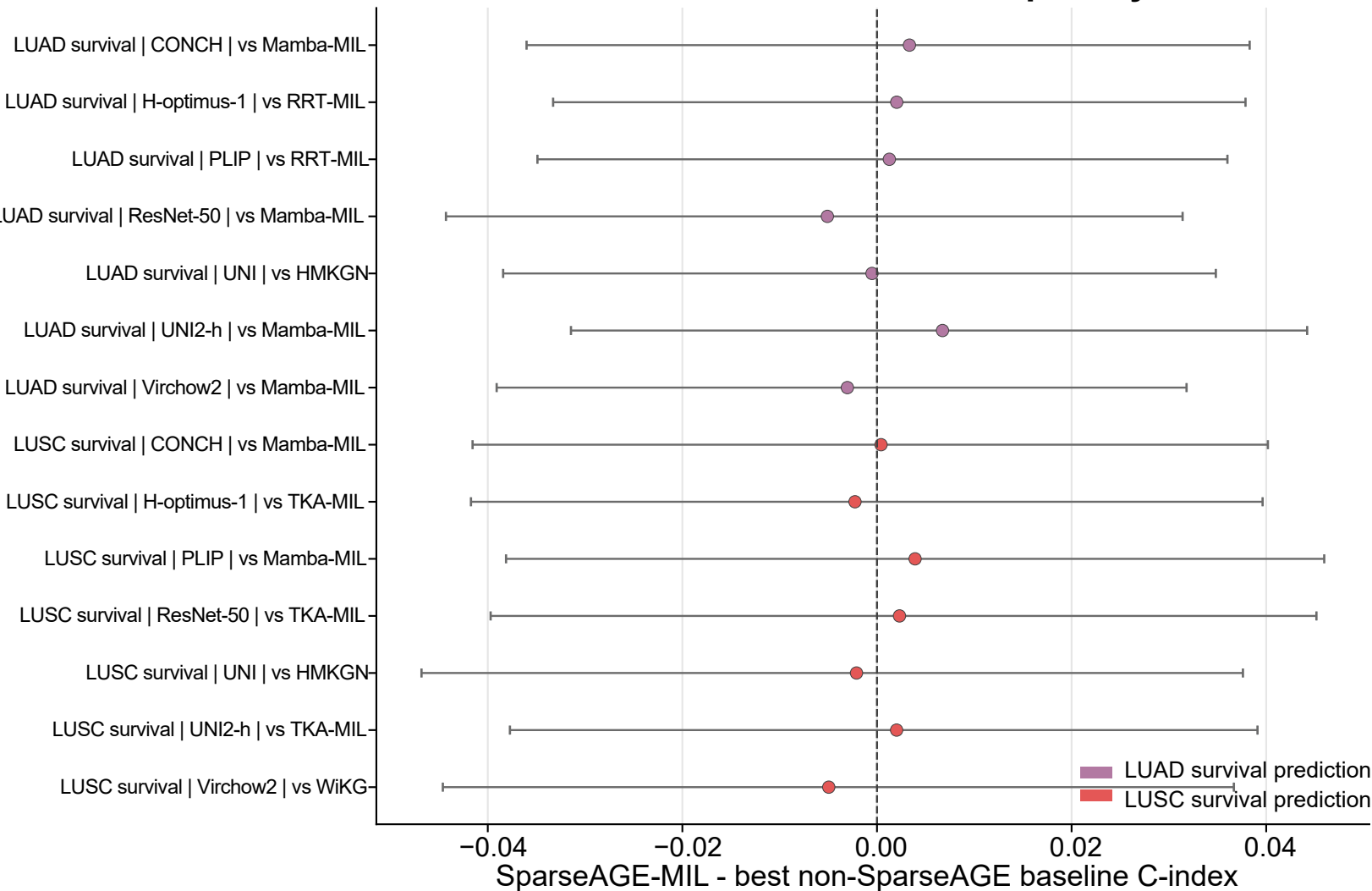

Supplement: Supplementary file 7 — Supporting Information [file CTM2-16-e70744-s001.pdf]

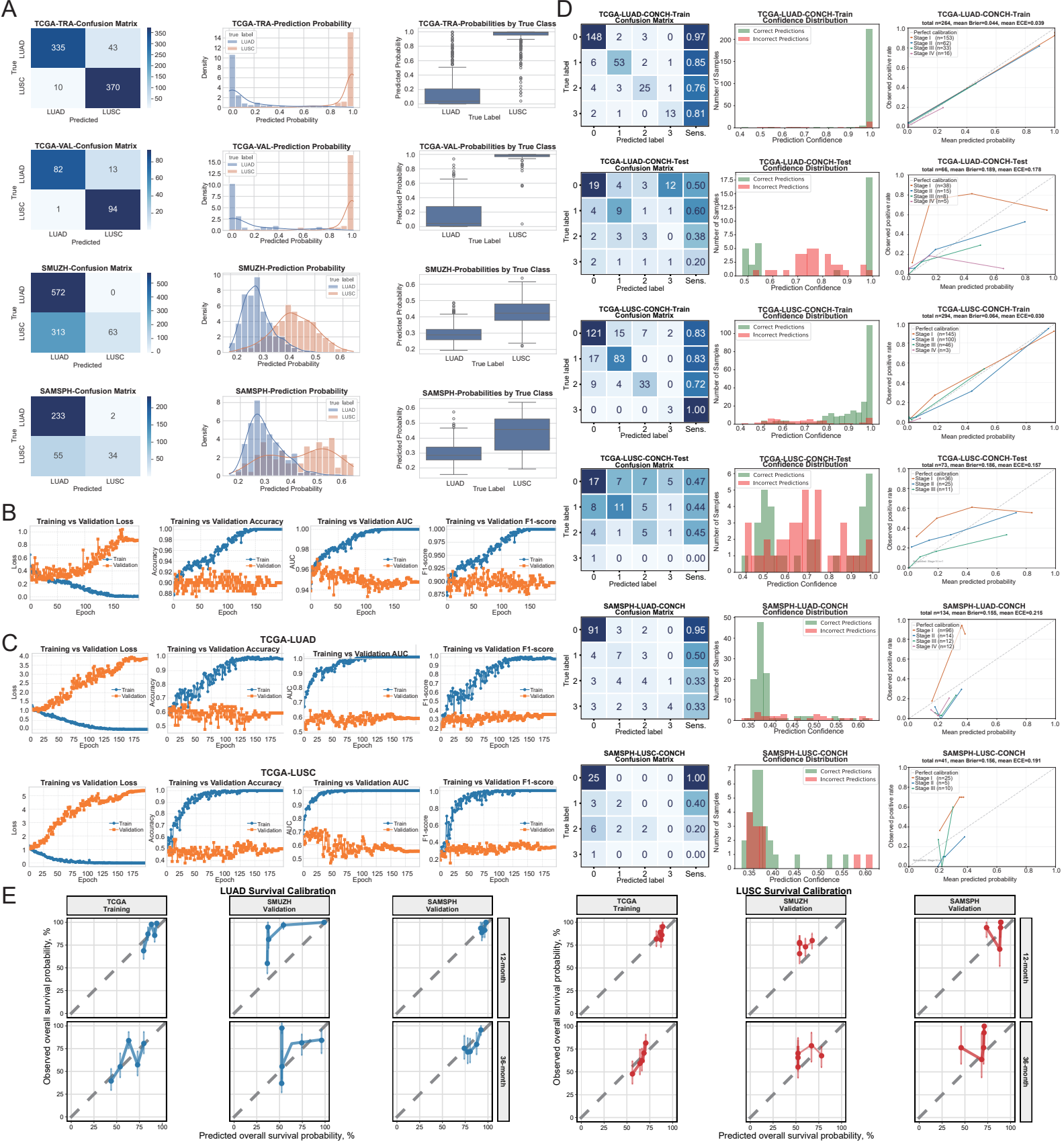

Supplement: Supplementary file 8 — Supporting Information [file CTM2-16-e70744-s016.pdf]
