# Supplementary material for: An interpretable multi‐task whole‐slide histopathology AI model for non‐small cell lung cancer: Cross‐cohort generalisation, spatial attention–transcriptomic integration, and molecular–immune profiling
Source: Clin Transl Med. 2026 Jul 23;16(7):e70744. doi: 10.1002/ctm2.70744 (PMC13396892; doi:10.1002/ctm2.70744)
Supplement: Supplementary file 5 — Supporting Information [file CTM2-16-e70744-s011.pdf]

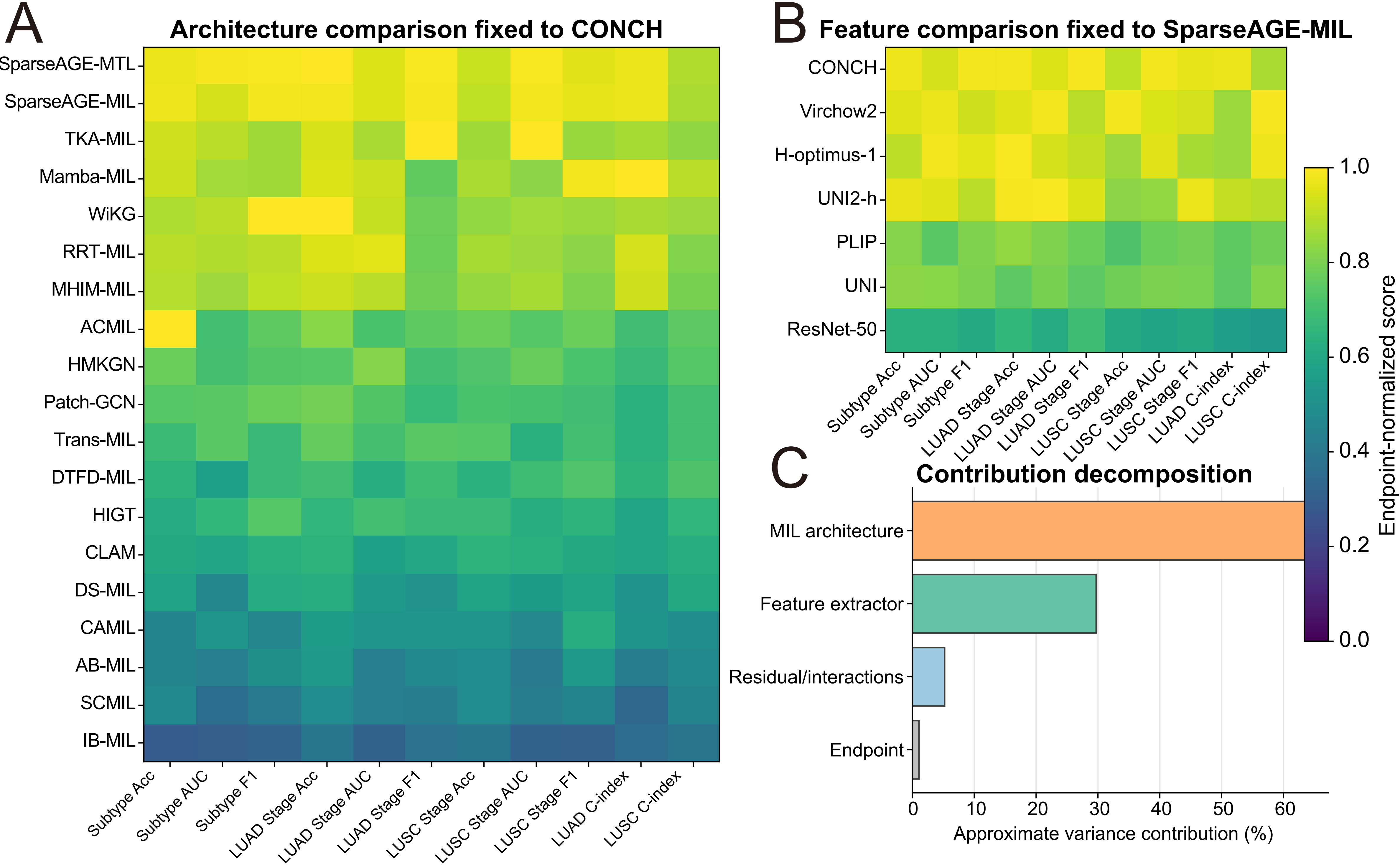
A
Architecture comparison fixed to CONCH
SparseAGE-MTL
SparseAGE-MIL
TKA-MIL
Mamba-MIL
WiKG
RRT-MIL
MHIM-MIL
ACMIL
HMKGN
Patch-GCN
Trans-MIL
DTFD-MIL
HIGT
CLAM
DS-MIL
CAMIL
AB-MIL
SCMIL
IB-MIL
Subtype Acc
Subtype AUC
Subtype F1
LUAD Stage Acc
LUAD Stage AUC
LUAD Stage F1
LUSC Stage Acc
LUSC Stage AUC
LUSC Stage F1
LUAD C-index
LUSC C-index
B
Feature comparison fixed to SparseAGE-MIL
CONCH
Virchow2
H-optimus-1
UNI2-h
PLIP
UNI
ResNet-50
Subtype Acc
Subtype AUC
Subtype F1
LUAD Stage Acc
LUAD Stage AUC
LUAD Stage F1
LUSC Stage Acc
LUSC Stage AUC
LUSC Stage F1
LUAD C-index
LUSC C-index
Endpoint-normalized score
1.0
0.8
0.6
0.4
0.2
0.0
C
Contribution decomposition
MIL architecture
Feature extractor
Residual/interactions
Endpoint
0
10
20
30
40
50
60
Approximate variance contribution (%)
