# Supplementary material for: An interpretable multi‐task whole‐slide histopathology AI model for non‐small cell lung cancer: Cross‐cohort generalisation, spatial attention–transcriptomic integration, and molecular–immune profiling
Source: Clin Transl Med. 2026 Jul 23;16(7):e70744. doi: 10.1002/ctm2.70744 (PMC13396892; doi:10.1002/ctm2.70744)
Supplement: Supplementary file 9 — Supporting Information [file CTM2-16-e70744-s018.pdf]

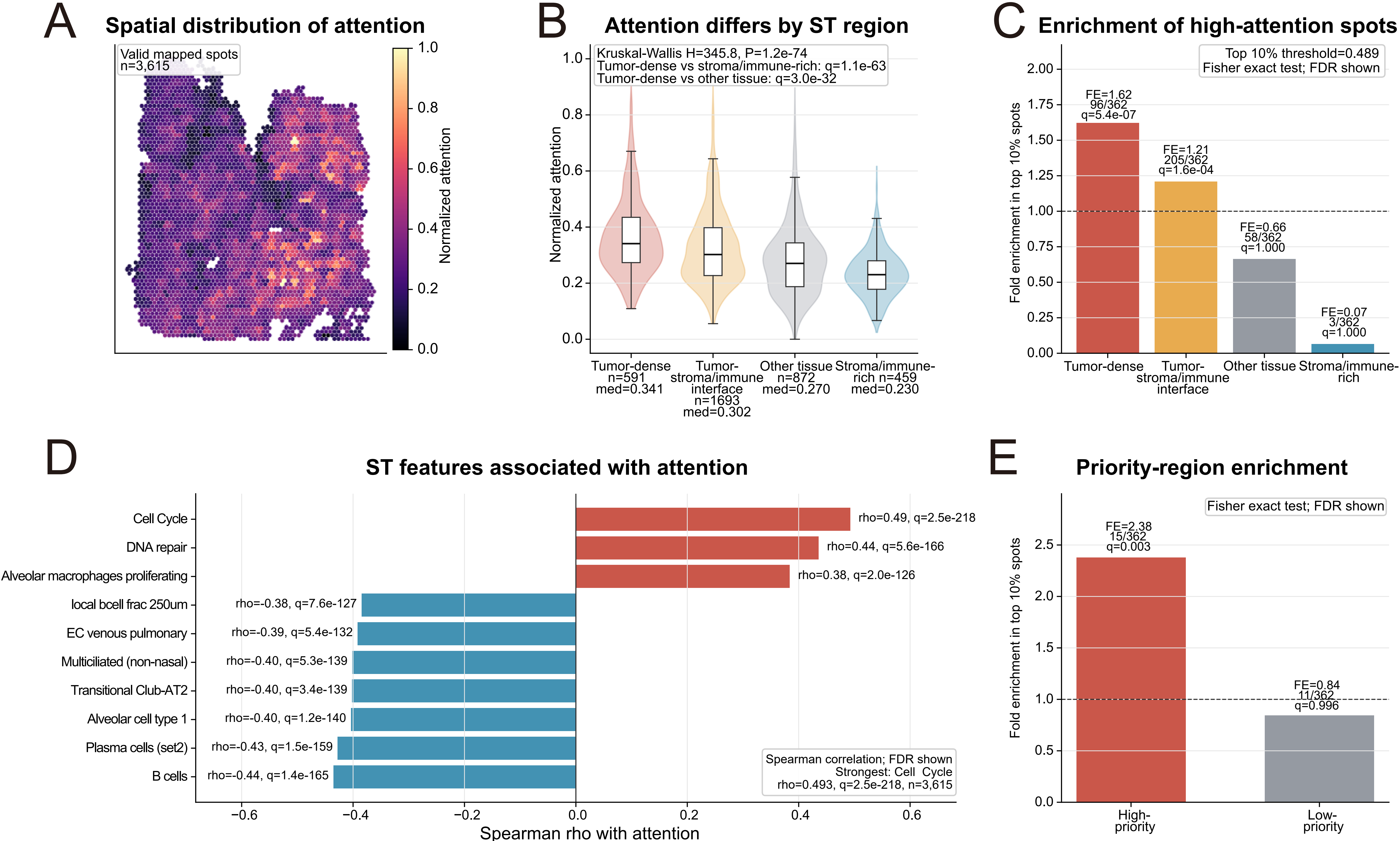

A
Spatial distribution of attention
Valid mapped spots
n=3,615
Normalized attention
B
Attention differs by ST region
Kruskal-Wallis H=345.8, P=1.2e-74
Tumor-dense vs stroma/immune-rich: q=1.1e-63
Tumor-dense vs other tissue: q=3.0e-32
Normalized attention
Tumor-dense n=591 med=0.341
Tumor-stroma/immune interface n=1693 med=0.302
Other tissue n=872 med=0.270
Stroma/immune-rich n=459 med=0.230
C
Enrichment of high-attention spots
Top 10% threshold=0.489
Fisher exact test; FDR shown
Fold enrichment in top 10% spots
FE=1.62 96/362 q=5.4e-07
FE=1.21 205/362 q=1.6e-04
FE=0.66 58/362 q=1.000
FE=0.07 3/362 q=1.000
Tumor-dense
Tumor-stroma/immune interface
Other tissue
Stroma/immune-rich
D
ST features associated with attention
Cell Cycle
DNA repair
Alveolar macrophages proliferating
local bcell frac 250um
EC venous pulmonary
Multiciliated (non-nasal)
Transitional Club-AT2
Alveolar cell type 1
Plasma cells (set2)
B cells
rho=0.49, q=2.5e-218
rho=0.44, q=5.6e-166
rho=0.38, q=2.0e-126
rho=-0.38, q=7.6e-127
rho=-0.39, q=5.4e-132
rho=-0.40, q=5.3e-139
rho=-0.40, q=3.4e-139
rho=-0.40, q=1.2e-140
rho=-0.43, q=1.5e-159
rho=-0.44, q=1.4e-165
Spearman correlation; FDR shown
Strongest: Cell Cycle
rho=0.493, q=2.5e-218, n=3,615
Spearman rho with attention
E
Priority-region enrichment
Fisher exact test; FDR shown
Fold enrichment in top 10% spots
FE=2.38 15/362 q=0.003
FE=0.84 11/362 q=0.996
High-priority
Low-priority
