# Supplementary material for: An interpretable multi‐task whole‐slide histopathology AI model for non‐small cell lung cancer: Cross‐cohort generalisation, spatial attention–transcriptomic integration, and molecular–immune profiling
Source: Clin Transl Med. 2026 Jul 23;16(7):e70744. doi: 10.1002/ctm2.70744 (PMC13396892; doi:10.1002/ctm2.70744)
Supplement: Supplementary file 10 — Supporting Information [file CTM2-16-e70744-s017.pdf]

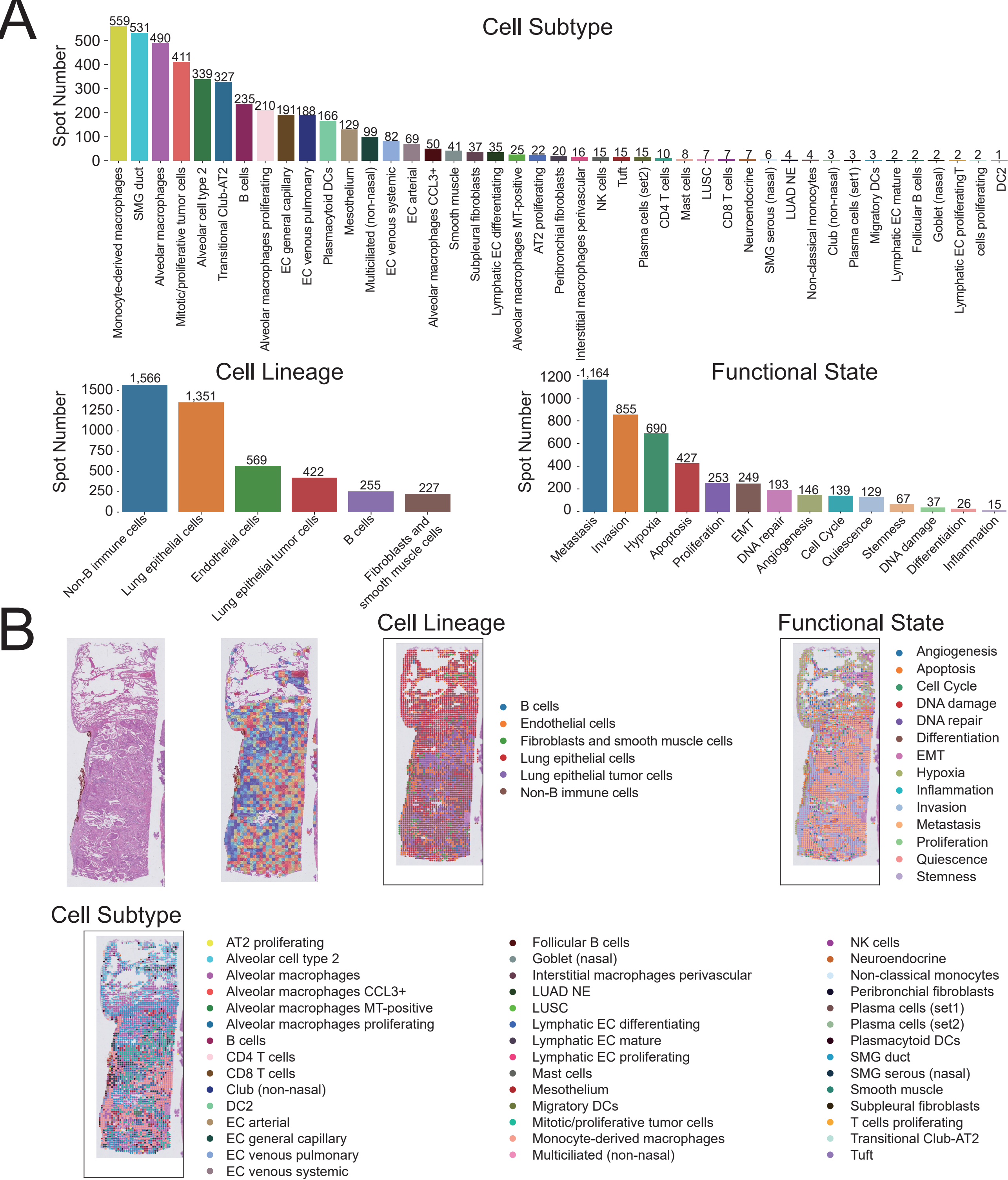

A
Cell Subtype
Spot Number
0
100
200
300
400
500
559
531
490
411
339
327
235
210
191
188
166
129
99
82
69
50
41
37
35
25
22
20
16
15
15
15
10
8
7
7
7
6
4
4
3
3
3
2
2
2
2
2
1
Monocyte-derived macrophages
SMG duct
Alveolar macrophages
Mitotic/proliferative tumor cells
Alveolar cell type 2
Transitional Club-AT2
B cells
Alveolar macrophages proliferating
EC general capillary
EC venous pulmonary
Plasmacytoid DCs
Mesothelium
Multiciliated (non-nasal)
EC venous systemic
EC arterial
Alveolar macrophages CCL3+
Smooth muscle
Subpleural fibroblasts
Lymphatic EC differentiating
Alveolar macrophages MT-positive
AT2 proliferating
Peribronchial fibroblasts
Interstitial macrophages perivascular
NK cells
Tuft
Plasma cells (set2)
CD4 T cells
Mast cells
LUSC
CD8 T cells
Neuroendocrine
SMG serous (nasal)
LUAD NE
Non-classical monocytes
Club (non-nasal)
Plasma cells (set1)
Migratory DCs
Lymphatic EC mature
Follicular B cells
Goblet (nasal)
Lymphatic EC proliferatingT
cells proliferating
DC2
Cell Lineage
Spot Number
0
250
500
750
1000
1250
1500
1,566
1,351
569
422
255
227
Non-B immune cells
Lung epithelial cells
Endothelial cells
Lung epithelial tumor cells
B cells
Fibroblasts and smooth muscle cells
Functional State
Spot Number
0
200
400
600
800
1000
1200
1,164
855
690
427
253
249
193
146
139
129
67
37
26
15
Metastasis
Invasion
Hypoxia
Apoptosis
Proliferation
EMT
DNA repair
Angiogenesis
Cell Cycle
Quiescence
Stemness
DNA damage
Differentiation
Inflammation
B
Cell Lineage
B cells
Endothelial cells
Fibroblasts and smooth muscle cells
Lung epithelial cells
Lung epithelial tumor cells
Non-B immune cells
Functional State
Angiogenesis
Apoptosis
Cell Cycle
DNA damage
DNA repair
Differentiation
EMT
Hypoxia
Inflammation
Invasion
Metastasis
Proliferation
Quiescence
Stemness
Cell Subtype
AT2 proliferating
Alveolar cell type 2
Alveolar macrophages
Alveolar macrophages CCL3+
Alveolar macrophages MT-positive
Alveolar macrophages proliferating
B cells
CD4 T cells
CD8 T cells
Club (non-nasal)
DC2
EC arterial
EC general capillary
EC venous pulmonary
EC venous systemic
Follicular B cells
Goblet (nasal)
Interstitial macrophages perivascular
LUAD NE
LUSC
Lymphatic EC differentiating
Lymphatic EC mature
Lymphatic EC proliferating
Mast cells
Mesothelium
Migratory DCs
Mitotic/proliferative tumor cells
Monocyte-derived macrophages
Multiciliated (non-nasal)
NK cells
Neuroendocrine
Non-classical monocytes
Peribronchial fibroblasts
Plasma cells (set1)
Plasma cells (set2)
Plasmacytoid DCs
SMG duct
SMG serous (nasal)
Smooth muscle
Subpleural fibroblasts
T cells proliferating
Transitional Club-AT2
Tuft
