# Supplementary material for: An interpretable multi‐task whole‐slide histopathology AI model for non‐small cell lung cancer: Cross‐cohort generalisation, spatial attention–transcriptomic integration, and molecular–immune profiling
Source: Clin Transl Med. 2026 Jul 23;16(7):e70744. doi: 10.1002/ctm2.70744 (PMC13396892; doi:10.1002/ctm2.70744)
Supplement: Supplementary file 11 — Supporting Information [file CTM2-16-e70744-s007.pdf]

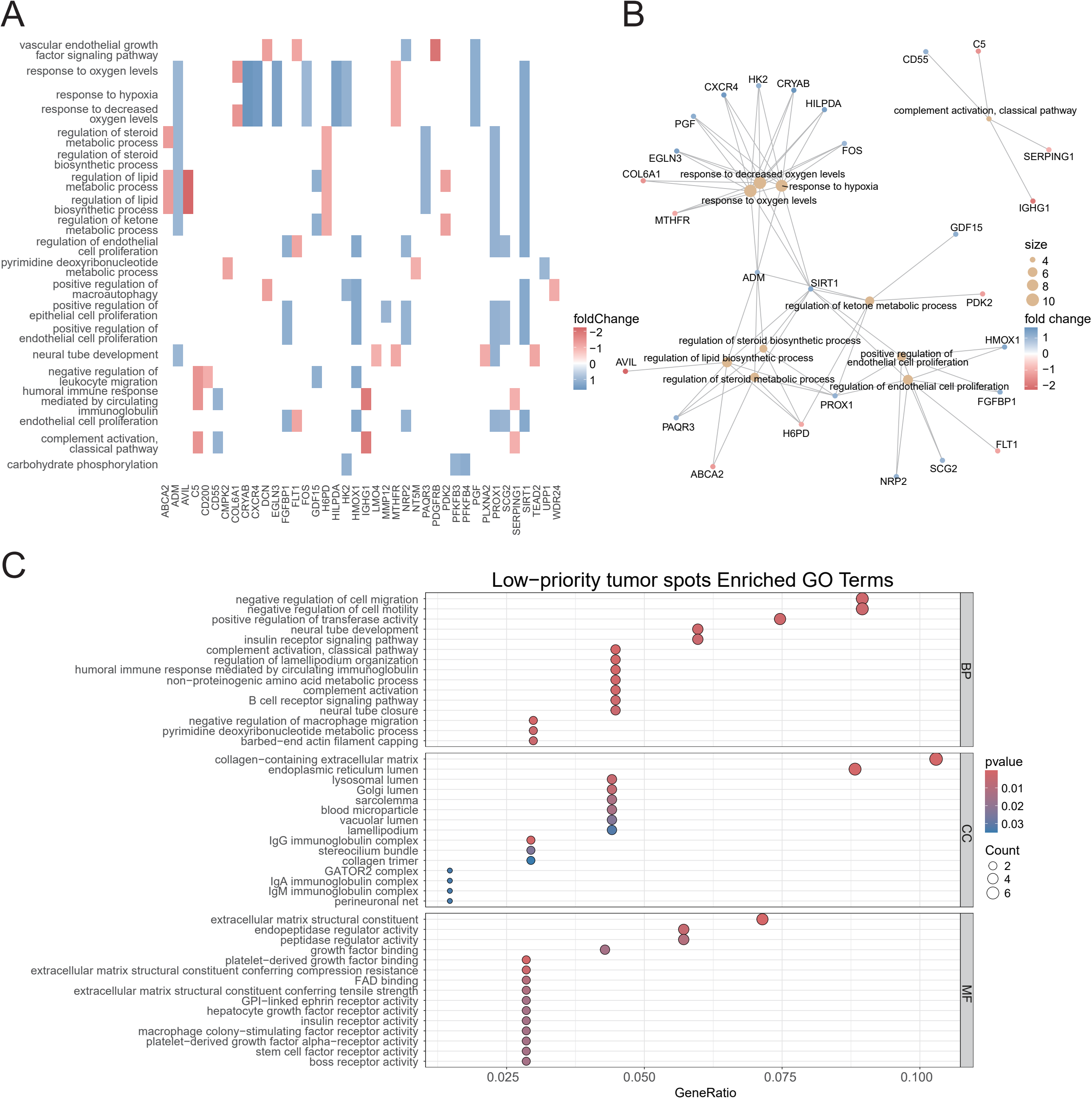
A
vascular endothelial growth factor signaling pathway
response to oxygen levels
response to hypoxia
response to decreased oxygen levels
regulation of steroid metabolic process
regulation of steroid biosynthetic process
regulation of lipid metabolic process
regulation of lipid biosynthetic process
regulation of ketone metabolic process
regulation of endothelial cell proliferation
pyrimidine deoxyribonucleotide metabolic process
positive regulation of macroautophagy
positive regulation of epithelial cell proliferation
positive regulation of endothelial cell proliferation
neural tube development
negative regulation of leukocyte migration
humoral immune response mediated by circulating immunoglobulin
endothelial cell proliferation
complement activation, classical pathway
carbohydrate phosphorylation
ABCA2 ADM AVIL C5 CD200 CD55 CMPK2 COL6A1 CRYAB CXCR4 DCN EGLN3 FGFBP1 FLT1 FOS GDF15 H6PD HILPDA HK2 HMOX1 IGHG1 LMO4 MMP12 MTHFR NRP2 NT5M PAQR3 PDGFRB PDK2 PFKFB3 PFKFB4 PGF PLXNA2 PROX1 SCG2 SERPING1 SIRT1 TEAD2 UPP1 WDR24
foldChange
−2
−1
0
1
B
CD55
C5
complement activation, classical pathway
SERPING1
IGHG1
CXCR4
HK2
CRYAB
HILPDA
PGF
FOS
EGLN3
COL6A1
response to decreased oxygen levels
response to hypoxia
response to oxygen levels
MTHFR
GDF15
ADM
SIRT1
regulation of ketone metabolic process
PDK2
regulation of steroid biosynthetic process
AVIL
regulation of lipid biosynthetic process
positive regulation of endothelial cell proliferation
HMOX1
regulation of steroid metabolic process
regulation of endothelial cell proliferation
PROX1
FGFBP1
PAQR3
H6PD
ABCA2
FLT1
SCG2
NRP2
size
4
6
8
10
fold change
1
0
−1
−2
C
Low−priority tumor spots Enriched GO Terms
negative regulation of cell migration
negative regulation of cell motility
positive regulation of transferase activity
neural tube development
insulin receptor signaling pathway
complement activation, classical pathway
regulation of lamellipodium organization
humoral immune response mediated by circulating immunoglobulin
non−proteinogenic amino acid metabolic process
complement activation
B cell receptor signaling pathway
neural tube closure
negative regulation of macrophage migration
pyrimidine deoxyribonucleotide metabolic process
barbed−end actin filament capping
BP
collagen−containing extracellular matrix
endoplasmic reticulum lumen
lysosomal lumen
Golgi lumen
sarcolemma
blood microparticle
vacuolar lumen
lamellipodium
IgG immunoglobulin complex
stereocilium bundle
collagen trimer
GATOR2 complex
IgA immunoglobulin complex
IgM immunoglobulin complex
perineuronal net
CC
extracellular matrix structural constituent
endopeptidase regulator activity
peptidase regulator activity
growth factor binding
platelet−derived growth factor binding
extracellular matrix structural constituent conferring compression resistance
FAD binding
extracellular matrix structural constituent conferring tensile strength
GPI−linked ephrin receptor activity
hepatocyte growth factor receptor activity
insulin receptor activity
macrophage colony−stimulating factor receptor activity
platelet−derived growth factor alpha−receptor activity
stem cell factor receptor activity
boss receptor activity
MF
0.025
0.050
0.075
0.100
GeneRatio
pvalue
0.01
0.02
0.03
Count
2
4
6
